# Effectiveness and safety of opioid-free anesthesia compared to opioid-based anesthesia: a systematic review and network meta-analysis

**DOI:** 10.1186/s44158-025-00272-9

**Published:** 2025-08-13

**Authors:** Vincenzo Francesco Tripodi, Salvatore Sardo, Mariachiara Ippolito, Andrea Cortegiani

**Affiliations:** 1 Anesthesia and Intensive Care, Human Pathology Department, University Hospital “Gaetano Martino”, Via Consolare Valeria 1, Messina, 98124 Italy; 2 Department of Medical Sciences and Public Health, University of Cagliari, Strada Provinciale 8, Monserrato, 09042 Italy; 3 Department of Anesthesia, Analgesia, Intensive Care and Emergency, University Hospital Policlinico Paolo Giaccone, Via del Vespro 129, Palermo, 90127 Italy; 4 Department of Precision Medicine in Medical, Surgical and Critical Care (Me.Pre.C.C.), University of Palermo, Via del Vespro 129, Palermo, 90127 Italy

**Keywords:** OFA, Opioid-free anesthesia, Postoperative pain, Postoperative nausea and vomiting (PONV), Opioid, Recovery, Opioid consumption, Analgesia, Safety

## Abstract

**Background:**

Opioid-free anesthesia (OFA) is an innovative approach to anesthesia management aimed at enhancing both the safety and the quality of perioperative outcomes. The efficacy and safety of these approaches are uncertain. The aim of our work was to compare the effectiveness and safety of different OFA regimens to opioid-based anesthesia (OBA).

**Study design and methods:**

We conducted a systematic review and frequentist random-effects network meta-analysis of randomized controlled trials (RCTs). The primary outcome measure was the intensity of postoperative pain at 24 h, expressed in terms of numerical rating scale (NRS), visual analogue scale (VAS), or verbal rating scale (VRS) scores. The SUCRA was used to determine the likelihood that an intervention was ranked as the best. The certainty of the evidence was assessed according to the GRADE methodology for Network Meta-analysis (NMA).

**Results:**

A total of 42 RCTs were included, for a total of 4666 patients. We have addressed the variety of available interventions. The random-effects network meta-analysis comparing OBA and different OFA regimens showed no difference in the pain intensity at 24 h. We performed the GRADE assessment for each comparison between each OFA regimen and OBA as a comparator. The certainty of evidence for the primary outcome ranges from moderate to very low among the different comparisons.

**Conclusions:**

We have identified a significant heterogeneity in OFA regimens evaluated and a moderate to high risk of bias in over 70% of studies reporting the primary outcome. No OFA regimens showed a statistically significant effect over OBA in reducing postoperative pain within the first 24 h following surgery. Current evidence does not support the superiority of the analgesic efficacy of OFA in the immediate postoperative period compared to the use of opioids.

**Trial registration:**

This study is registered in PROSPERO with the registration number CRD42024529236 (May 3, 2024).

**Supplementary Information:**

The online version contains supplementary material available at 10.1186/s44158-025-00272-9.

## Introduction

Opioid-free anesthesia (OFA) represents a multimodal approach to pain management and a transformative approach to anesthesia, addressing the growing concerns surrounding opioid use. Traditionally, opioids have been a cornerstone of anesthetic protocols, valued for their potent analgesic properties and their ability to modulate pain during and after surgical procedures. However, the significant potential adverse effects, including respiratory depression, nausea, constipation and, most notably, the risk of addiction and opioid-induced hyperalgesia (a paradoxical increase in pain sensitivity) have  prompted the medical community to look for innovative strategies to minimize or eliminate the use of opioids in anesthetic practice, giving rise to the concept of OFA [1].

OFA relies on the use of a combination of non-opioid medications and techniques to achieve effective analgesia and anesthesia. These alternatives include agents such as local anesthetics, alpha-2 adrenergic agonists (e.g. dexmedetomidine), N-methyl-D-aspartate (NMDA) receptor antagonists (e.g. ketamine), magnesium sulfate and nonsteroidal anti-inflammatory drugs (NSAIDs) [2, 3]. Additionally, neuraxial blocks and regional anesthesia techniques, such as ultrasound-guided nerve blocks, play a crucial role in targeting specific areas of pain without affecting the entire body. This tailored approach not only reduces the use of opioids but also enhances patient recovery by minimizing opioid-related side effects, improving functional outcomes, and reducing hospital stays [4].

The benefits of OFA may extend beyond individual patient care to address broader public health challenges. By reducing opioid exposure in perioperative settings, OFA could contribute to mitigating the risk of long-term opioid dependency as a critical factor in combating the opioid epidemic [5]. Clinical studies suggest that patients receiving OFA experience faster recovery times, less postoperative nausea and vomiting (PONV) and improved overall satisfaction, making it a valuable option for elective and emergency procedures alike [6, 7]. These advantages have sparked growing interest in OFA across various surgical specialties, including orthopedics, gynecology and bariatrics, where effective pain management is essential for optimal outcomes [8].

Despite worldwide scientific interest, the association of OFA with clinical outcomes is still unclear. Several heterogeneous pharmacological strategies have been tested under the term “OFA”. This systematic review and network meta-analysis aimed to summarize the current evidence from randomized controlled trials (RCTs) and to evaluate the effectiveness and safety of the different OFA strategies [9, 10].

## Methods

The protocol for this systematic review was prospectively registered in the PROSPERO international database (CRD42024529236). The reporting adheres to the Preferred Reporting Items for Systematic Reviews and Meta-Analyses (PRISMA) extension for network meta-analysis. We reported the “PRISMA extension for network meta-analysis checklist” in Supplementary Material [11].

We conducted a systematic search across the PubMed-MEDLINE, Embase and Cochrane Central Register of Controlled Trials databases, covering the period from inception to November 27, 2024. The search focused on RCTs having opioid-free anesthesia (OFA) as the intervention and opioid-based anesthesia as the comparator, in populations of adult patients undergoing noncardiac surgery. OFA was defined as any multimodal strategy that does not involve the administration of intraoperative systemic, neuraxial, or intracavitary opioids alongside anesthetic agents. The population of interest was adult patients undergoing noncardiac surgery. The primary outcome was postoperative pain intensity at 24 h. The search strategy employed is detailed in Supplementary Material.

After removing duplicates, the records were screened by two investigators (M.I. and V.F.T.) based on titles and abstracts in a blinded manner. A third investigator (S.S.) conducted periodic surveys of additional sources employing the snowballing method. During the full-text review process, two investigators (M.I. and V.F.T.) selected relevant records, which were included based on a consensus agreement regarding eligibility. In cases of disagreement, eligibility was determined through consensus involving a third investigator (A.C.). Abstracts and conference proceedings were excluded from consideration. Studies focusing exclusively on pediatric populations were also excluded, along with those with unclear reporting of all inclusion criteria or those whose authors did not respond to requests for clarification or integrations about missing patients, outcome data or study methods. We also excluded studies whose intervention or comparator included locoregional anesthesia techniques in order to reduce clinical heterogeneity. The web-based software Rayyan facilitated the duplicate removal and the screening processes [12].

The primary outcome measure was the intensity of postoperative pain at 24 h, expressed in terms of numerical rating scale (NRS), visual analogue scale (VAS) or verbal rating scale (VRS) scores. Additional outcomes of interest included pain intensity within  two hours following emergence from anesthesia, postoperative opioid consumption within 48 h expressed as oral morphine equivalents, the number of postoperative rescue analgesia requests, the incidence of postoperative nausea or vomiting (PONV), duration of hospital stay and the occurrence of adverse events as reported by the authors.

Data extraction from the included studies was conducted in duplicate by three authors (M.I., S.S., and V.F.T.), utilizing a standardized electronic data extraction form through the RedCap platform [13, 14]. The extracted data encompassed study design, surgical setting, country, inclusion and exclusion criteria, patient characteristics, intervention characteristics, comparisons and outcome measures. In instances of uncertainty regarding data interpretation, we asked the corresponding authors via email for clarification about methodology or outcome data. In two instances, the data were extracted or confirmed by extracting the numerical values from published plots using the WebPlotDigitizer software [15].

Two authors independently assessed the risk of bias for the primary outcome (postoperative pain at 24 h) using Cochrane revised tool for assessing risk of bias in randomized trials Rob2 tool [16]. Discrepancies were resolved through consensus among four authors (M.I., S.S., V.F.T. and A.C.). Visualization of the risk of bias assessment was executed using the Robvis tool [17].

The certainty of the evidence was evaluated utilizing the Grading of Recommendations, Assessment, Development, and Evaluation (GRADE) approach for network meta-analysis [18]. For the primary outcome, we classified the certainty of each comparison as high, moderate, low or very low, considering factors such as risk of bias, reporting bias, indirectness, imprecision, heterogeneity and incoherence (the difference between direct and indirect effect). We established the null effect as the threshold for assessing imprecision. The CINeMA tool was employed for the analysis and reporting of confidence under the GRADE for network meta-analysis (NMA) framework, as recommended by Cochrane [19]. We referred to the publication by Laigaard et al. to set the threshold for the minimal clinically important difference at 1.5 points [20]. This assessment was conducted by one author (A.C.) and validated through consensus among the other authors (M.I., S.S., V.F.T. and A.C.).

We have performed a frequentist random-effects network meta-analysis using the netmeta R package [21]. The network geometry was graphically summarized using the BUGSnet R package [22]. Effect sizes for dichotomous outcomes were estimated as risk ratios (RRs), while effect sizes for continuous outcomes were expressed as standardized mean differences for pain intensity scores and mean differences for the other continuous outcomes. The precision of these effect sizes was presented using 95% confidence intervals (CIs). In cases of multi-arm trials, each arm was treated as a standalone intervention within the network meta-analysis framework. Dexmedetomidine and clonidine were merged as alpha agonists, while nitrous oxide and halogenated anesthetics were clustered under inhalation anesthesia. Interventions related to opioid-based anesthesia were clustered into a single comparator node for the purposes of this analysis.

Based on the desirability of each outcome and effect size estimates obtained from the network meta-analysis, the distribution of treatment ranks was established. The surface under the cumulative ranking curve (SUCRA), which indicates the degree of confidence in a treatment surpassing all other treatments, was then used to describe the hierarchy of treatments [23].

Heterogeneity was assessed using the I^2^ statistic, and publication bias was assessed visually with a funnel plot. In the case of arms with zero events, a 0.5 continuity correction was applied.

Pairwise comparisons were also investigated by random-effects frequentist pairwise meta-analysis using the R package meta [24]. The PRISMA flowchart was plotted with the R package PRISMA 2020 [25].

## Results

**Fig. 1:**
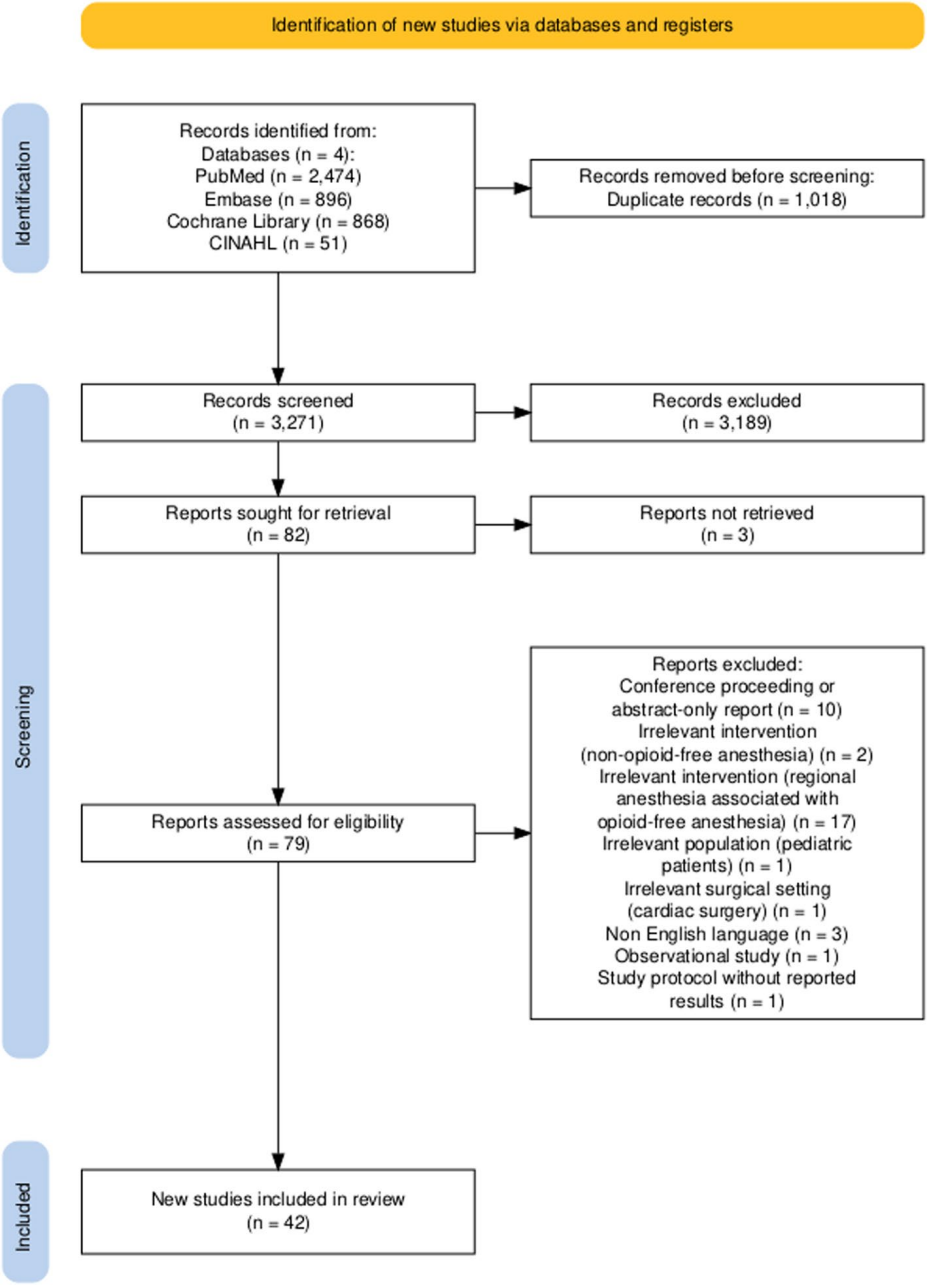
Preferred Reporting Items for Systematic reviews and Meta-Analyses (PRISMA) flowchart

We identified a total of 3271 unique records. The final meta-analysis comprised 42 RCTs, including 4666 patients [2, 3, 6, 7, 26–63]. The inclusion and exclusion process is depicted in Fig. 1 as a PRISMA flow diagram. The characteristics of the included studies are detailed in Table 1. Supplementary Table 1 provides a summary of the reasons for the exclusion of studies based on full-text assessments. Notably, only three of the included studies were designed as multicentric trials. The predominant surgical settings were endoscopic general surgery (23.81%), gynecologic surgery (21.43%), breast surgery (14.29%) and endoscopic bariatric surgery (11.9%). Twenty-eight studies included patients undergoing only endoscopic surgeries. There was considerable variability observed in the anesthesia regimens, drug doses and combinations employed across the studies. The interventions examined in the included studies consisted of the following: clonidine ( one study as a single bolus, one study as an infusion), dexmedetomidine ( two studies as a bolus, sixteen studies as an infusion, two studies with repeated boluses), ketamine (seven studies as a single bolus, ten studies as an infusion, two studies with repeated boluses), intravenous lidocaine (six studies as a single bolus, twelve studies as an infusion) and magnesium sulfate (three studies as a bolus, seven studies as an infusion).

**Table 1:** Characteristics of the included studies

Study	Design	Country	Surgery	Inclusion criteria	Exclusion criteria	Primary outcome	Sample	Interventions
Aboelela 2021	Monocentric	Egypt	Gynecologic Surgery	1. ASA I- II 2. Age 18–65 years old 3. Scheduled for abdominal gynecological surgery	1. Patient refusal to participate in the study 2. Known allergy to the study drugs 3. Addiction 4. Psychological troubles 5. Major cardiopulmonary disorders 6. Altered kidney function 7. Altered liver function 8. Altered thyroid function 9. Complicated surgery	Not declared	68	1. IV propofol 1–2 mg/kg + IV fentanyl 1 µg/kg followed by infusion 1 µg/kg/h + IV atracurium 0.6 mg/kg 2. IV propofol 1–2 mg/kg + IV ketamine 0.5 mg/kg + IV lidocaine 1.5 mg/kg followed by infusion 1.5 mg/kg/h + IV atracurium 0.6 mg/kg
Feng 2024	Monocentric	China	Thoracic Surgery (endoscopic procedures only)	1. Age ≥ 18 years 2. ASA I-III 3. Body mass index (BMI) 18–30 kg/m^2^ 4. Scheduled for elective VATS lung resection	1. Sick sinus syndrome or severe bradycardia (heart rate (HR) < 50 beats/min) 2. Second-degree or greater atrioventricular block without a pacemaker 3. Left ventricular ejection fraction < 40% 4. Coronary heart disease or history of myocardial infarction 5. Liver or renal dysfunction (Child–Pugh class C or undergoing renal replacement therapy) 6. Parkinson’s disease or Alzheimer’s disease 7. Seizures or epilepsy 8. Pregnancy or breast feeding 9. History of chronic pain or preoperative use of sedatives or analgesics 10. Allergies to medications in this study	Postoperative nausea and vomiting incidence	120	1. Dexmedetomidine 0.6 µg/kg bolus and 0.2–1 µg/kg/h infusion + esketamine 0.3 mg/kg bolus and maintenance boluses of 0.1 mg/kg + propofol 1.5–2.0 mg/kg bolus + sevoflurane 1–3% + flurbiprofen axetil 50 mg + sufentanil PCA 2. Sufentanil 0.3 µg/kg bolus and maintenance boluses of 0.1 µg/kg + propofol 1.5–2 mg/kg bolus + sevoflurane 1–3% + flurbiprofen axetil 50 mg + sufentanil PCA
Goyal 2017	Monocentric	India	Breast Surgery	1. ASA I–II 2. Age 18–75 years 3. Elective breast cancer surgery	1. Allergy to study drugs 2. Chronic analgesic medication, opioids or substance abuse 3. ASA III–IV 4. Obesity 5. Neurological or psychiatric disease 6. Cardiorespiratory disease 7. Renal disease 8. Hepatic disease 9. Refusal	Arterial pressure and heart rate	60	1. Loading dose of fentanyl 2 μg/kg with maintenance dose of 0.5 μg/kg/h 2. Loading dose of dexmedetomidine 1 μg/kg with maintenance dose of 0.25 μg/kg/h
Greiss 2021	Monocentric	Egypt	General Surgery (endoscopic procedures only)	1. Elective laparoscopic surgery under general anesthesia ≤ 2 h 2. Age18–65 years 3. ASA I-II 4. Body mass index 18.5–29.9	1. Anticipated difficult intubation 2. History of myocardial, pulmonary, or endocrine diseases 3. Diabetes mellitus, hepatic or renal impairment 4. Drug abuse or opioid addiction 5. Surgical complication 6. Failure of laparoscopy	Metabolic stress response and moprhine consumption	91	1. Dexmedetomidine 1 μg/kg as a loading dose over 10 min prior to induction, followed by 0.2–0.7 μg/kg/h till 10 min before the end of surgery 2. Fentanyl 1 μg/kg as a loading dose over 10 min prior to induction, followed by 0.2–0.7 μg/kg/h till 10 min before the end of surgery
Hao 2023	Monocentric	China	General Surgery (endoscopic procedures only)	1. ASA I-II 2. Age 20–60 years 3. Elective laparoscopic cholecystectomy surgery	1. Severe heart, lung disease and psychiatric disease 2. Hypotension, bradycardia and atrioventricular block 3. Allergy to study drugs 4. Pregnancy, breastfeeding or menstruating women 5. BMI > 30 kg/m^2^	Quality of recovery (QoR-15 questionnaire)	80	1. Dexmedetomidine 0.6 μg/kg bolus and 0.5–0.7 μg/kg/h infusion + esketamine 0.2 mg/kg bolus and 0.2–0.5 mg/kg/h + propofol 4–8 mg/kg/h + sevoflurane MAC 0.8–1.4 2. Sufentanil 0.3–0.5 μg/kg bolus + remifentanil 0.1–0.3 μg/kg/min + sevoflurane MAC 0.8–1.4
Akman 2023	Monocentric	Turkey	General Surgery Bariatric Surgery (endoscopic procedures only)	1. Scheduled to undergo laparoscopic bariatric surgery under general anesthesia 2. ASA II-III	1. Arrhythmias that could affect the Nociception Level (NOL) Index measurement 2. Beta-blocker drugs 3. Peripheral vascular disease 4. History of allergy to the drugs 5. Intraoperative arrhythmia 6. Pulmonary and surgical complications 7. Hemodynamic stabilization could not be easily provided 8. Monitoring interrupted	Nociception Level Index	40	1. Dexmedetomidine 0.5 µg/kg/h + Magnesium sulfate 30 mg/kg as an IV infusion in 100 ml of saline for 10 min + 1.5 mg/kg lidocaine IV bolus 2. Remifentanil at 0.05–0.1 µg/kg/min
Hontoir 2016	Monocentric	Belgium	Breast Surgery	1. Oncological patients undergoing a total mastectomy or lumpectomy associated with a total axillary dissection 2. ASA II 3. Knowledge of either French, English or Dutch	1. Allergy or contraindications to one of the study drugs 2. Renal failure 3. Hepatic failure 4. Hyperthyroidism 5. AV block 2 or 3 or severe bradycardia 6. Left ventricular failure 7. Unstable blood pressure 8. Epilepsy 9. Psychiatric disturbance	QoR-40 (quality of recovery)	66	1. IV clonidine (0.2 µg/kg), ketamine (0.3 mg/kg), lidocaine (1.5 mg/kg) and propofol (2–3 mg/kg); maintenance used sevoflurane (MAC: 1) adapted for hemodynamic stability, with acetaminophen (1000 mg) and diclofenac (75 mg) given upon incision, additional ketamine boluses (0.2 mg/kg, up to three), piritramide (0.03 mg/kg) upon subcutaneous closure, and postoperative pain managed with IV acetaminophen (1000 mg every 6 h), diclofenac (75 mg every 12 h) and a PCA pump of piritramide 2. IV remifentanil TCI, ketamine (0.3 mg/kg), lidocaine (1.5 mg/kg) and propofol (2–3 mg/kg); maintenance used sevoflurane (MAC: 1), with acetaminophen (1000 mg) and diclofenac (75 mg) given upon incision, piritramide (0.03 mg/kg) upon subcutaneous closure and postoperative pain managed with IV acetaminophen (1000 mg every 6 h), diclofenac (75 mg every 12 h), and a PCA pump of piritramide
Beloeil 2021	Multicentric	France	General Surgery Orthopedic Surgery Otolaryngology (ENT) Surgery Urology Vascular Surgery Gynecologic Surgery Colorectal Surgery Endocrine Surgery	1. Patients older than 18 years 2. Planned for major or intermediate scheduled surgery 3. Affiliated to a social security system 4. Had given written informed consent	1. Known allergies to any of the drugs used for anesthesia or to any of their excipients; 2. pregnancy or breastfeeding; 3. urgent surgery; 4. intracranial surgery; 5. transplant surgery or transplanted patients; 6. surgery with planned regional anesthesia; 7. outpatient surgery; 8. atrioventricular, intraventricular, or sinoatrial block; 9. Adam-Stokes syndrome; 10. patients chronically treated with beta blockers and heart rate of fewer than 50 beats/min; 11. cardiac insufficiency with a left ventricular ejection fraction of less than 40%; 12. epilepsy or seizures; 13. acute cerebral pathology; 14. obstructive sleep apnea syndrome; 15. patients with a preoperative oxygen saturation measured by pulse oximetry (Spo2) less than 95%; 16. severe hepatic insufficiency (defined as prothrombin ratio less than 15%); 17. adults legally protected (under judicial protection, guardianship, or supervision); 18. persons deprived of their liberty; or patients in whom the Confusion Assessment Method could not be performed	Components of the composite primary outcome were postoperative hypoxemia, defined as a Spo_2_ < 95% with a need for oxygen supplementation; postoperative ileus, defined as an absence of flatus or stools; and postoperative cognitive dysfunction, evaluated using the Confusion Assessment Method	314	1. Propofol (1.5 to 2 mg/kg) and then desflurane, IV lidocaine (1.5 mg/kg bolus plus 1.5 mg kg^−1^ h^−1^), IV ketamine (0.5 mg/kg bolus plus 0.25 mg kg^−1^ h^−1^), neuromuscular blockade, and dexamethasone (8 mg, IV bolus) and IV dexmedetomidine administered at the infusion rate of 0.4 to 1.4 μg kg^−1^ h^−1^ (dexmedetomidine group 2. propofol (1.5 to 2 mg/kg) and then desflurane, IV lidocaine (1.5 mg/kg bolus plus 1.5 mg kg^−1^ h^−1^), IV ketamine (0.5 mg/kg bolus plus 0.25 mg kg^−1^ h^−1^), neuromuscular blockade, and dexamethasone (8 mg, IV bolus) and IV remifentanil, using effect site target-controlled infusion mode (3 to 5 ng/ml corresponding to 0.1 to 0.25 µg kg^−1^ min^−1^; remifentanil group)
Campos‐Pérez 2022	Monocentric	Mexico	General Surgery (endoscopic procedures only)	1. Patients aged 18 to 60 years; 2. BMI > 30 kg/m^2^; 3. scheduled to undergo a gastric bypass after an integrated preoperative evaluation; 4. signed the informed consent	1. Patients with a history of ischemic heart disease, history of drug abuse, and with any known allergy to any of the drugs used during anesthesia	Cytokine levels	40	1. TIVA (Opioid-containing anesthesia): Fentanyl: 3 mcg/kg bolus (loading) + 0.003–0.006 µg/kg/min (maintenance); Propofol: 2–2.5 Cp via TCI with the Cortínez-Sepúlveda model (real weight) (loading AND maintenance); Ketamine: 0.15 mg/kg (loading) + 0.15 mg/kg/min (maintenance) + 0.5 mg/kg (post-operative); Lidocaine 2%: 1 mg/kg (loading) + 1 mg/kg (maintenance) + 1 mg/kg (post-operative); Magnesium sulfate: 30–50 mg/kg (loading) + 10 mg/kg/min (maintenance) + 5 mg/kg (post-operative); Rocuronium bromide: 0.6 mg/kg bolus (loading) + 1.25 µg/kg/min (maintenance); Metamizole (post-operative): 30 mg/kg; Buprenorphine (post-operative): 1 µg/kg; Paracetamol (post-operative): 1 g every 12 h 2. OFA (Opioid-free anesthesia): Dexmedetomidine: 1–1.5 µg/kg (loading) + 0.3–0.7 µg/kg/min (maintenance); Propofol: 2.5–3.5 Cp via TCI with the Cortínez-Sepúlveda model (real weight) (loading) + 2–4 Cp (maintenance); Ketamine: 0.15 mg/kg (loading) + 0.15 mg/kg/min (maintenance) + 0.5 mg/kg (post-operative); Lidocaine 2%: 1 mg/kg (loading) + 1 mg/kg (maintenance) + 1 mg/kg (post-operative); Magnesium sulfate: 30–50 mg/kg (loading) + 10 mg/kg/min (maintenance) + 5 mg/kg (post-operative); Rocuronium bromide: 0.6 mg/kg bolus (loading) + 1.25 µg/kg/min (maintenance); Metamizole (post-operative): 30 mg/kg; Paracetamol (post-operative): 1 g every 12 h
Bhardwaj 2019	Monocentric	India	Urology (endoscopic procedures only)	1. Age 20–60 years; 2. ASA II–III; 3. BMI ≥ 30 kg/m^2^; 4. laparoscopic urological procedures under general anesthesia	1. History of known allergies to study drugs; 2. opioid use 1 month prior to surgery or chronic opioid addiction; 3. inability to comprehend visual analogue scale (VAS); 4. pregnant or lactating mothers; 5. a history of significant hepatic, renal, or cardiac disease; 6. patients in whom the surgical procedure was converted to the open procedure were excluded		80	1. Group 1 (Fentanyl and Propofol): Fentanyl: 2 mcg/kg; Propofol: 2.5–3.5 mg/kg; Atracurium: 0.5 mg/kg; Maintenance: Propofol 50–200 µg/kg/min + intermittent fentanyl 0.5 µg/kg bolus; Goal: BIS 40–60; 2. group 2 (Dexmedetomidine & Propofol): Dexmedetomidine: 0.5 µg/kg loading over 10 min; Propofol: 2.5–3.5 mg/kg; Atracurium: 0.5 mg/kg; Maintenance: Propofol 50–200 µg/kg/min + dexmedetomidine 0.1–0.3 µg/kg/h; Lignocaine: 1.5 mg/kg at induction + 0.1 mg/kg/h infusion; Ketamine: 0.5 mg/kg before incision; Goal: BIS 40–60
Cha 2023	Monocentric	Colombia	Gynecologic Surgery (endoscopic procedures only)	1. Female patients; 2. age: 18–65 years; 3. American Society of Anesthesiologists Physical Status Class I–II; 4. scheduled for elective hysteroscopy	1. History of drug abuse; 2. refusal to provide consent; 3. history of psychotropic medication or psychological disorders; 4. treatment using angiotensin-converting enzyme inhibitors; 5. gastro-esophageal reflux; 6. morbid obesity (BMI ≥ 30 kg/m^2^); 7. allergy to any of the study drugs; 8. use of medications or nutraceuticals that affect blood pressure (BP) or heart rate (HR); 9. surgical procedure exceeding one hour in duration; 10. unexpected bleeding complications; 11. repeated laryngeal mask airway insertion attempts	Quality of recovery 24 h postoperatively as assessed using the QoR-40 questionnaire	90	1. 0.3 μg kg^−1^ sufentanil + 1.5 mg kg^−1^ h^−1^ lidocaine + 2.0 mg kg^−1^ propofol and 1 mg kg^−1^ scoline + 0.3 mg kg^−1^ of rocuronium + 2–3% sevoflurane; 2. 1.5 mg kg^−1^ lidocaine + 0.9% saline + 2.0 mg kg^−1^ propofol and 1 mg kg^−1^ scoline + 0.3 mg kg^−1^ of rocuronium + 2–3% sevoflurane
Choi 2022	Monocentric	South Korea	Gynecologic Surgery (endoscopic procedures only)	1. Patients aged 20–65 years; 2. scheduled for elective gynecological laparoscopy at Seoul St. Mary’s Hospital, the Catholic University of Korea	1. Emergency surgery; 2. cancer surgery; 3. chronic pain requiring medication; 4. history of psychiatric disease; 5. hypotension; 6. bradycardia; 7. atrioventricular block; 8. intraventricular or sinoatrial block; 9. body mass index > 35 kg/m^2^; 10. known allergies; 11. history of adverse events to any of the drugs used for anesthesia; 12. pregnancy; 13. lactation	Quality of recovery on postoperative day (POD) 1 measured using the Quality of Recovery-40 (QoR-40) questionnaire scores	78	1. Dexmedetomidine: 0.7 μg/kg IV loading dose for 10 min before induction; continuous infusion: 0.5 μg/kg/h; Lidocaine: 1.5 mg/kg IV bolus + 1.5 mg/kg/h infusion; Propofol: 1.5–2 mg/kg; Rocuronium: 0.8 mg/kg IV; Maintenance: 4–6% desflurane; 2. Remifentanil: Target-controlled infusion to achieve an effector site concentration of 3.5 ng/ml; Propofol: 1.5–2 mg/kg; Rocuronium: 0.8 mg/kg IV; Maintenance: 4–6% desflurane
Clanet 2024	Monocentric		General Surgery (endoscopic procedures only)	1. Obesity class III; 2. age > 18 years; 3. scheduled for elective laparoscopic gastric bypass surgery; 4. written informed consent	1. Known allergies to any of the drugs used for anesthesia or to any of their excipients; 2. pregnancy or breastfeeding; 3. atrioventricular, intraventricular, or sinoatrial block; 4. patients with a heart rate < 50 beats/min at the preoperative consultation	Total morphine consumption in the first 24 h after surgery	172	1. Dexmedetomidine: 0.4–0.8 µg/kg IBW/h; Lidocaine 2%: 49 ml + Ketamine 50 mg (1 ml/10 kg IBW/h); Ketamine: 25 mg; Lidocaine: 1.5 mg/kg IBW; Propofol: 2 mg/kg TBW; Rocuronium: 1.2 mg/kg IBW; Paracetamol: 15 mg/kg TBW; Diclofenac: 75 mg; Dexamethasone: 1 0 mg; Ondansetron: 4 mg; Sevoflurane; Dexmedetomidine (maintenance): 0.4–0.8 µg/kg/h; Rocuronium (maintenance): 0.1 mg/kg IBW if post-tetanic count > 1/10; 2. Remifentanil: 0.2–0.4 µg/kg/min IBW; Saline 0.9%: 1 ml/10 kg IBW/h; Ketamine: 25 mg; Lidocaine: 1.5 mg/kg IBW; Propofol: 2 mg/kg TBW; Rocuronium: 1.2 mg/kg IBW; Paracetamol: 15 mg/kg TBW; Diclofenac: 75 mg; Dexamethasone: 10 mg; Ondansetron: 4 mg; Sevoflurane; Remifentanil (maintenance): 0.2–0.4 µg/kg/min; Rocuronium (maintenance): 0.1 mg/kg IBW if post-tetanic count > 1/10
Das 2022	Monocentric	India	Plastic and Reconstructive Surgery Otolaryngology (ENT) Surgery	1. Head and neck cancer surgery; 2. Aged between 18 and 60 years; 3. patient belonging to ASA I-II	1. Any known allergy to study drugs; 2. patient with bradycardia (HR < 60/min); 3. surgeries exceeding > 5 h; 4. patients on ventilator in the postoperative period; 5. patient refusal		62	1. IV lignocaine 1.5 mg/kg, IV Dexmedetomidine 0.5 µg/kg and IV Ketamine 0.5 mg/kg + IV midazolam 1 mg and glycopyrrolate 0.2 mg + IV propofol; 2. IV fentanyl 2 µg/kg + IV midazolam 1 mg and glycopyrrolate 0.2 mg + IV propofol
Jose 2023	Monocentric	India	Breast Surgery	1. ASA physical status I–II; 2. elective modified radical mastectomy surgery; 3. age 18–65 years; 4. BMI 18–30 kg/m^2^	1. Suspect of pregnancy; 2. lactation; 3. allergy to study drugs; 4. atrioventricular nodal block; 5. beta-blocker use; 6. autonomic dysfunction; 7. chronic pain; 8. chronic use of analgesics	Hemodynamic parameters	120	1. IV morphine 0.15 mg/kg before anesthesia + normal saline infusion + 20 ml of 0.25% bupivacaine infiltration at the surgical site; 2. IV dexmedetomidine 1 µg/kg before anesthesia + 0.5 µg/kg/h + IV lignocaine 1.5 mg/kg before anesthesia + 1.5 mg/kg/h
Khaled 2023	Monocentric	Egypt	Orthopedic Surgery (endoscopic procedures only)	1. ASA status I-II 2. age > 65 years old 3. elective arthroscopic shoulder surgery under general anesthesia	1. Uncontrolled systemic diseases; 2. significant organ dysfunctions; 3. morbid obesity (BMI > 35); 4. history of allergy to the study drugs; 5. use of beta blockers; 6. chronic use of opioids	Arterial pressure and surgical field condition	30	1. Dexmedetomidine 1 µg/kg loading over 10 min + continuous infusion 0.3 µg/kg/h + lidocaine 2 mg/kg/h + magnesium sulfate 1.5 g/h; 2. Fentanyl 2 µg/kg loading + continuous infusion 1 µg/kg/h + saline syringes for blinding
Kumar 2023	Monocentric	India	General Surgery (endoscopic procedures only)	1. ASA status class I–II; 2. age 20–60 years; 3. elective laparoscopic surgeries lasting for < 2 h	1. Pregnant, breast-feeding women; 2. hepatic, renal or cardiac insufficiency; 3. psychiatric disease; 4. allergy or contraindication to study drugs; 5. BMI > 30 kg/m^2^; 6. Obstructive sleep apnoea (OSA) syndrome	Post-operative pain intensity and need of rescue analgesics	70	1. Dexmedetomidine 1 mcg/kg + ketamine 25 mg IV before induction; 2. Fentanyl 1 µg/kg IV before induction
Kurhekar 2023	Monocentric	India	Gynecologic Surgery	1. Elective minor gynecological procedure 2. Age 18–60 years; 3. ASA status I–II	1. BMI < 18 or > 30; 2. chronic opioid treatment or chronic pain conditions; 3. cardiovascular disease, hypertension; 4. not willing to participate in the study	Recovery time	56	1. Dexmedetomidine 1 µg/kg in 100 ml normal saline as infusion over ten minutes + propofol 2 mg/kg; 2. Fentanyl 2 mcg/kg in 100 ml normal saline as infusion over ten minutes + propofol 2 mg/kg
Luong 2020	Monocentric	Vietnam	General Surgery Hepatobiliary Surgery (endoscopic procedures only)	Laparoscopic cholecystectomy	1. Disagreement to enroll in the study; 2. history of epilepsy; 3. history of mental illness; 4. communication difficulties; 5. history of increased intracranial pressure; 6. heart disease; 7. hypertension; 8. bradycardia; 9. liver failure; 10. kidney failure; 11. pregnancy; 12. nursing mother	Efficacy and side effects of free opioid anesthesia	94	1. Lidocaine 2 mg/kg before induction + 1.5 mg/kg/h for maintenance + magnesium 30 mg/kg before induction + 1.5 g infusion for maintenance + ketamine 0.5 mg/kg IV + ketorolac 30 mg IV; 2. Fentanyl 5 µg/kg IV for induction + 1.5 µg/kg every 30 min for maintenance of anesthesia
Mansour 2013	Monocentric	Egypt	Bariatric Surgery (endoscopic procedures only)	1. BMI > 50 kg/m^2^; 2. laparoscopic sleeve gastrectomy	1. Age < 18 years; 2. positive pregnancy test; 3. history of drug abuse or opioid drug dependency; 4. patients with chronic pain; 5. patients with severe cardiac, pulmonary, liver, or neurological disease	Hemodynamic parameters	28	1. Propofol 2 mg/kg IV + Ketamine 0.5 mg/kg IV + Rocuronium 0.5 mg/kg IV + Sevoflurane 2–4% + Ketamine infusion 0.5 mg/kg/h + Paracetamol 1 g/6 h IV for 24 h + Diclofenac 75 mg/12 h IM for 48 h + Tramadol 50–100 mg/12 h IV prn for 24 h + Tramadol PCA: Concentration 10 mg/ml, Dose 1 ml, lock-out period 6 min, no basal infusion 2. Propofol 2 mg/kg IV + Fentanyl 2–5 µg/kg IV + Rocuronium 0.5 mg/kg IV + Sevoflurane 2–4% + Fentanyl infusion 0.025–0.25 µg/kg/min + Paracetamol 1 g/6 h IV for 24 h + Fentanyl PCA: Concentration 10 µg/ml, dose 1 ml, lock-out interval 6 min
Massoth 2021	Monocentric	Germany	Gynecologic Surgery (endoscopic procedures only)	1. Female sex; 2. age > 18 years; 3. elective inpatient gynecological laparoscopy	1. Pregnancy; 2. breastfeeding; 3. history of chronic pain or intake of any sedatives and analgesics; 4. allergies or contraindications to any study drugs; 5. participation in another interventional trial	Incidence of PONV up to 24 h post-op	157	1. Dexmedetomidine 0.6 µg/kg + Esketamine 0.15 mg/kg + Propofol 1–2 mg/kg + Rocuronium 0.6 mg/kg + Dexmedetomidine 0.3 µg/kg/h + Esketamine 0.15 mg/kg/h + Sevoflurane (MAC 1.0–1.4); 2. Sufentanil 0.3 µg/kg + Propofol 1–2 mg/kg + Rocuronium 0.6 mg/kg + Sufentanil 0.15 µg/kg boluses as needed + Sevoflurane (MAC 0.8–1.0)
Menck 2022	Monocentric	Brazil	Bariatric Surgery (endoscopic procedures only)	1. Adult patients; 2. BMI ≥ 35 kg/m^2^; 3. elective laparoscopic Roux-en-Y gastric bypass performed by the same surgeon	1. Refusal to participate in the study; 2. chronic pain; 3. chronic use of analgesics; 4. any condition or pathology that could affect pain perception; 5. heart block or significant arrhythmia; 6. patients anesthetized differently from the proposed protocol or by a professional who did not participate in the study	Pain intensity and morphine use for rescue analgesia	60	1. Opioid-Free Anesthesia (OFA) Group: Dexmedetomidine 0.5 µg/kg + Magnesium sulfate 40 mg/kg + Ketamine 25 mg + Lidocaine 1.5–2 mg/kg + Propofol + Rocuronium 1.2 mg/kg + Sevoflurane 0.9–1 MAC + Rocuronium 10 mg every 40 min + Clonidine 75 µg + Magnesium sulfate 2.5 g + Lidocaine 2% (1 ml/kg/h); 2. Fentanyl Group: Fentanyl 2.5 µg/kg bolus + Propofol + Rocuronium 1.2 mg/kg + Sevoflurane 0.9–1 MAC + Rocuronium 10 mg every 40 min
Mieszczański 2023	Monocentric	Poland	Bariatric Surgery (endoscopic procedures only)	1. BMI > 40 or > 35 with comorbidities; 2. sleeve gastrectomy; 3. written informed consent	1. Patient’s refusal; 2. known allergies to study medication; 3. inability to comprehend or participate in pain scoring scale; 4. inability to use intravenous patient controlled analgesia; 5. changes of operation extent during procedure; 6. revisional operations	Opioid consumption	60	1. OFA Group: Dexmedetomidine 1 µg/kg IBW + Lidocaine 1.5 mg/kg IBW + Ketamine 0.5 mg/kg IBW + Propofol 2–2.5 mg/kg + Dexmedetomidine 1 µg/kg/h + Lidocaine 3 mg/kg/h + Magnesium sulfate 40–50 mg/kg IBW + Fentanyl 100 µg (rescue) + Oxycodone 0.1 mg/kg IBW IV + Paracetamol 1 g IV every 6 h + Metamizole 1 g IV every 6 h + PCA Oxycodone (bolus 2 mg, lockout 10 min); 2. MMA Group: Remifentanil TCI (induction 6 ng/ml, maintenance adjusted) + Propofol 2–2.5 mg/kg + Sevoflurane + Rocuronium or Cis-atracurium + Sugammadex or Neostigmine with Atropine + Oxycodone 0.1 mg/kg IBW IV + Paracetamol 1 g IV every 6 h + Metamizole 1 g IV every 6 h + PCA Oxycodone (bolus 2 mg, lockout 10 min)
Saravanaperumal 2022	Monocentric	India	Gynecologic Surgery	1. ASA physical status I and II; 2. aged 23 to 38 years; 3. ultrasound (USG) showing > 3 bilateral ovarian follicular response	1. ASA III patients; 2. history of cardiac/renal/liver disease; 3. BMI > 35 kg/m 2; 4. USG showing less than 3 dominant follicle	Quality of recovery, QOR-15	66	1. Dexmedetomidine 0.5 μg/kg over 10 min in 100 ml of normal saline as infusion, about 10 min prior to procedure. At the start of the procedure, another 0.5 μg/kg of dexmedetomidine was given as infusion over 15 min + Propofol 1.5 mg/kg/h ev; 2. fentanyl 1 μg/kg ev over 10 min in 100 ml of normal saline as infusion, about 10 min prior to procedure. At the start of the procedure, another 1 μg/kg of fentanyl was given + Propofol 1.5 mg/kg/h ev
Tochie 2022	Monocentric	Cameroon	Gynecologic Surgery	1. Adult non-pregnant women; 2. aged ≥ 18 years; 3. ASA I and II; 4. patient udergoing elective myomectomy, hysterectomy, ovarian cystectomy or total mastectomy for benign pathologies and localized malignancies	1. History of allergy to any drug used; 2. history of alcohol, opioid or drug abuse; 3. chronic pain; 4. psychiatric illness; 5. patients undergoing surgery with planned regional anesthesia of tissular infiltration of local anesthesia, those with iatrogenic surgical complications such as bowel, ureter or bladder injuries	1. faiqlure of OFA (defined as the intraoperative need to administer opioids for adequate intraoperative analgesia) and the occurrence of intra-operative complications	36	1. Premedication with lidocaine 1.5 mg/kg IV, magnesium sulfate 40 mg/kg (in 100 ml of saline without exceeding 2.5 g), ketamine 25 mg IV and dexamethasone 0.1 mg/kg IV. Induction of general anesthesia with propofol 1.5 mg/kg IV and rocuronium 0.1 mg/kg IV. Anesthesia was maintained using isoflurane between 0.5–2%, and an electric pump syringe at 10–15 ml/h containing a mixture of magnesium sulfate 40 mg/kg (without exceeding a total dose of 2.5 g/24 h taking note of the induction dose), lidocaine 1.5 mg/kg, ketamine 0.25 mg, and clonidine 1 µg/kg; 2. premedication with diazepam 5 mg IV and dexamethasone 0.1 mg/kg IV. The anesthesia was induced using fentanyl 3 µg/kg IV, propofol 2.5 mg/kg IV, and rocuronium 0.1 mg/kg IV. Anesthesia was maintained using isoflurane between 0.5–2%, reinjections of one-quarter of the induction dose of fentanyl every 20–30 min and one-quarter of the induction dose of propofol as needed and a continuous infusion of normal saline via an electric pump syringe at 10–15 ml/h as placebo
Toleska (a) 2019	Monocentric	North Macedonia	General Surgery (endoscopic procedures only)	1. Patients who were hospitalized for elective laparoscopic cholecystectomy; 2. ASA I, II, III; 3. Age between 25 and 60	1. Patients with ASA classification 4 and 5; 2. allergy to opioids (fentanyl and tramadol), lidocaine, magnesium, ketamine, paracetamol, ketonal and metamizole; 3. patients who chronically use benzodiazepines or opioids; 4.patients who are pregnant or are breastfeeding; 5. patients with chronic pain; 6. patients with cardiac, renal and hepatic failure; 7. patients with diabetes and psychiatric illness	The level of pain (VAS scores)	60	1. OFA Group (OFAG): Dexamethasone 0.1 mg/kg IV + Paracetamol 1 g IV (preemptive analgesia) + Midazolam 0.04 mg/kg IV + Lidocaine 1 mg/kg IV + Propofol 2 mg/kg IV + Rocuronium 0.6 mg/kg IV + Ketamine 0.5 mg/kg IV + Sevoflurane 0.7–1 MAC + Lidocaine infusion 2 mg/kg/h IV + Magnesium sulphate infusion 1.5 g/h IV; 2. Fentanyl Group (FG): Midazolam 0.04 mg/kg IV + Fentanyl 0.002 mg/kg IV + Propofol 2 mg/kg IV + Rocuronium 0.6 mg/kg IV + Sevoflurane 0.7–1 MAC
Toleska (b) 2022	Monocentric	North Macedonia	General Surgery (endoscopic procedures only)	1. Patient undergoing elective laparoscopic cholecystectomy; 2. ASA classification1; 3. informed consent	1. ASA classification 3, 4 and 5; 2. allergy to opioids (fentanyl and tramadol), lidocaine, magnesium, ketamine, paracetamol, ketonal and metamizole; 3. patients which chronically use benzodiazepines and opioids; 4. pregnant and breastfeeding women; 5. patients with the presence of chronic pain; 6. patients with heart, renal and hepatic failure; 7. patients with psychiatric illness	PONV	80	1. Fentanyl group—FG: midazolam 0.04 mg/kg, fentanyl 0.002 mg/kg; 2 mg/kg propofol and 0.6 mg/kg rocuronium bromide; 2. opioid free anesthesia group—OFAG: dexamethasone 0.1 mg/kg and 1 g of paracetamol before introduction to anesthesia as pre-emptive analgesia. Midazolam 0.04 mg/kg, lidocaine 1 mg/kg, propofol 2 mg/kg, ketamine 0.5 mg/kg, and 0.6 mg/kg rocuronium bromide. Immediately after intubation, continuous intravenous infusion with lidocaine 2 mg/kg/h and magnesium sulfate 1.5 g/h was given
Van Loocke 2022	Monocentric	Belize	Bariatric Surgery (endoscopic procedures only)	1. Patients > 18 years of age; 2. patients undergoing ndergoing an elective primary laparoscopic bariatric Roux & Y surgery; 3. informed consent	1. Patients with diabetes type 1 or diabetes type 2 (IV or antidiabetic medication); 2. glucose intolerance at the time of surgery or during pregnancy; 3. ASA IV patients; 4. patients with an addiction to opioids or chronic opioid use; 5. patients with allergy or contraindications to any of the drugs included for anesthesia, patients with major cardiovascular, pulmonary, liver or renal insufficiency before surgery; 6. patients planned for postoperative intensive care admission; 7. patients with a contra-indication for general anesthesia with intubation and mechanical ventilation	Glycemia level	39	1. OFA Group: dexmedetomidine in a loading dose of 0.25 µg/kg before incision followed by a continuous infusion of 0.1 µg/kg/h during surgery; Lidocaine in a loading dose of 1 mg/kg before incision followed by a continuous infusion of 1 mg/kg/h during surgery; Esketamine in a loading dose of 25 mg before incision followed by a continuous infusion of 0.05 mg/kg/h during surgery. Furthermore, a loading dose of 2.5 g Magnesium was given to every OFA patient; 2. OA Group: Sufentanil in a loading dose of 15–25 µg Sufentanil before incision, followed by additional 5–10 µg Sufentanil
Yasar 2023	Monocentric	Turkey	Bariatric Surgery (endoscopic procedures only)	1. ASA I-II-III group; 2. patients aged between 18 and 65; 3. patients undergoing bariatric surgery in general surgery operating rooms; 4. patients with a body mass index (BMI) ≥ 35 kg/m^2^, patients whose surgery performed under general anesthesia (from incision to closure) is not expected to exceed 2 h	1. Urgent surgery; 2. inability to provide consent; 3. ASA IV, V	Pain intensity and adverse events	64	1. Opioid Anesthesia Group: initial dose of 1 µg/kg for 30 to 60 s according to IBW 0.5–1 µg/kg/min by continuous IV infusion in induction of remifentanil as an opioid for analgesia. A continuous IV infusion of remifentanil 0.25 mcg/kg/min (range 0.05–2 µg/kg/min) was administered for maintenance; 2. Non Opioid Anesthesia Group: patients received an IV infusion of 1 g of paracetamol and an infusion of 400 mg of ibuprofen IV for analgesia 30 min before the incision. Before the incision in the perioperative period, 0.2 mg/kg IV bolus ketamine was administered as an IV bolus of 0.5 mg/kg according to IBW (ideal body weight) between 30–45 min of surgery. A 30 mg/kg bolus of magnesium sulfate was administered followed by a 10 mg/kg/h perioperative infusion
Yu 2023	Monocentric	China	General Surgery (endoscopic procedures only)	1. Patients undergoing laparoscopic cholecystectomy (LC) with use of LMA. 2. Patient with ASA I-II	1. Patients who were aged < 18 years or > 65 years; 2. patients with a body mass index (BMI) ≥ 30 kg/m^2^; 3. patients with hepatic or renal disease, coagulopathy, a history of alcohol or drug abuse; 4. Patients with ASA ≥ III; 5. patients with basal heart rate (HR) ≤ 50 beats/min; 6. patients with patients who were pregnant; 7. patients who had a past medical history of chronic pain; 8. patients who should not take NSAIDs; 9. patients with allergies to related medication; 10. patients with communication disorders	Use of rescue analgesic within 24 h after surgery	150	1. Opioid-free anesthesia (OFA) group: infusion of dexmedetomidine 0.6 μg/kg at a constant rate for 10 min (the participants in the OBA group received an infusion of the same dose of normal saline). Then, anesthesia was induced with a fixed protocol of propofol 2–3 mg/kg, lidocaine 1.5 mg/kg (followed by intravenous infusion at 2 mg kg^−1^ h^−1^ continuously but terminated when the gallbladder was extracted), and cisatracurium besilate 0.2 mg/kg. A single dose of 0.3 mg/kg esketamine was injected approximately 2 min before incision. A continuous intravenous infusion of 3–12 mg/kg/h of propofol was administered in either group; 2. opioid-based anesthesia (OBA) group: Anesthesia was induced with a fixed protocol of 2–3 mg/kg propofol, 1 μg/kg remifentanil (followed by continuous intravenous infusion of 0.1–0.3 μg/kg/min), and 0.2 mg/kg cisatracurium besilate. A continuous intravenous infusion of 3–12 mg/kg/h of propofol was administered in either group
Zhou 2023	Multicentric	China	Otolaryngology (ENT) Surgery	1. Patients aged at least 16 years; 2. informed consent; 3. patient scheduled for elective ESS; 4. operation time of at least 30 min; 5. ASA I–II	1. Known mental or nervous system diseases; 2. severe impairment of hearing, visual or language system functions; 3. cognitive impairment before surgery; 4. patients with chronic pain, identified or suspected opioid abuse or long-term use of narcotic sedatives and analgesics; 5. contraindications or allergies to opioid drugs; 6. not understanding or cooperating with the QoR-40 questionnaire or numeric rating scale (NRS)	24-h postoperative quality of recovery using the QoR-40 questionnaire	773	1. OFA Group: Dexmedetomidine 0.5 mcg/kg infused over 10 min, followed by lidocaine 1.5 mg/kg. Propofol 1.5 to 2 mg/kg, midazolam 0.05 mg/kg, cisatracurium 0.15 mg/kg, betamethasone 8 mg. During maintenance of anaesthesia, the OFA group received a continuous infusion of dexmedetomidine 0.5 µg/kg/h, lidocaine 1.5 mg/kg/h, sevoflurane (1% to 3%) and propofol 4 to 12 mg/kg/h; 2. OA Group: Sufentanil 0.3 µg/kg, Propofol 1.5 to 2 mg/kg, midazolam 0.05 mg/kg, cisatracurium 0.15 mg/kg, betamethasone 8 mg
Hu 2024	Monocentric	China	Gynecologic Surgery (endoscopic procedures only)	1. Women aged 18–65 years; 2. ASA I or II; 3. patient scheduled for elective laparoscopic gynecological surgery	1. Untreated underlying disease (e.g. hypertension, epilepsy, and diabetes); 2. body mass index (BMI) ≥ 30 kg/m^2^; 3. hemoglobin concentration ≤ 80 g/l; 4. severe cardiac arrhythmias (e.g. second-II-II atrioventricular block, atrial fibrillation and heart failure);;5. Consent refusal	48-h TWA of the NRS	74	1. Group C: propofol (2 mg/kg) and rocuronium (0.6 mg/kg); sufentanil (0.3 μg/kg); 2. Group F (OFA): propofol (2 mg/kg) and rocuronium (0.6 mg/kg); lidocaine (1.5 mg/kg) and esketamine (0.15 mg/kg)
Rani 2024	Monocentric	India	Neurosurgery	1. Patient undergoing spine surgery; 2. informed Consent	1. Refusal informed consent	Postoperative pain‐free period and postoperative pain scores	60	1. OBA—patients received OBA with fentanyl and propofol. Around 10 ml syringe (analgesic agent) contains fentanyl (in a dose of 2 μg/kg) diluted with saline to a total volume of 10 ml. Around 50 ml syringe (labeled as an anesthetic agent) contains 48 ml of propofol (10 mg/ml) [1 ml of propofol contains 10 mg of propofol]; 2. OFA—patients received OFA with ketamine and ketofol. Around 10 ml syringe (labeled as an analgesic agent) contains ketamine (in a dose of 1 mg/kg) diluted with saline to a total volume of 10 ml. Around 50 ml syringe (labeled as an anesthetic agent) contains ketofol (ketamine and propofol in a ratio of 1:5), which contains 8 ml of ketamine (10 mg/ml) and 40 ml of propofol (10 mg/ml) [1 ml of ketofol contains 1.67 mg of ketamine and 8.33 mg of propofol.]
Seyam 2024	Monocentric	El Salvador	Bariatric Surgery (endoscopic procedures only)	1. Patients undergoing bariatric surgery; 2. 20–60 years old, both males and females; 3. ASA-II and III; 4. BMI ranging from 35–50	1. Declined participation; 2. pregnant women; 3. patients with communication difficulties that could hinder a reliable postoperative assessment; 4. patients with comorbidities like uncontrolled hypertension, ischemic heart disease, uncontrolled diabetes mellitus and renal or liver impairment	Assess the effects of OFA utilizing the modified mulimix technique on the levels of plasma IL-2 and IL-6	60	1. OBA Group: Propofol 2 mg/kg; Cisatracurium 0.15 mg/kg; Intermittent boluses of cisatracurium; Sevoflurane 2%; Atropine and neostigmine. Opioid-containing anesthesia with fentanyl 2 μg/kg 10 min before the induction of anesthesia, then 0.5 μg/kg/h of fentanyl infusion was started at a rate of 20 ml/h throughout the surgery. Additionally, morphine 0.03 μg/kg was administered at the time of port placement; 2. OFA Group: Propofol 2 mg/kg; Cisatracurium 0.15 mg/kg; Intermittent boluses of cisatracurium; Sevoflurane 2%; Atropine and neostigmine. Multimodal infusion as follows; dexmedetomidine 1 mg/kg intravenously over 10 min before initiation of anesthesia, followed by dexmedetomidine infusion at a rate of 0.5 μg/kg/h throughout the time of surgery. The Modified Mulimix technique, consisting of dexmedetomidine 2.5 μg/ml, ketamine 2.5 mg/ml, and lignocaine 20 mg/mL, was continued throughout the time of surgery at a rate of 20 ml/h. If the patient’s weight was 110 kg, the infusion rate was increased by 10%
Wallden 2006	Monocentric	Switzerland	General Surgery (endoscopic procedures only)	1. Patients undergoing laparoscopic cholecystectomy; 2. 3	1. Converted to open cholecystectomy; 2. duration of surgery exceeded 150 min; 3. refusal informed consent	The primary endpoints in the study were the gastric emptying parameters, and we tested the hypothesis that there would be a difference in gastric emptying between the study groups	50	1. TIVA Group with Opioid: premedication with midazolam 1–2 mg IV; anesthesia was induced with an infusion of remifentanil 0.2 μg kg^−1^ min^−1^, followed, after 2 min, by a target-controlled infusion (TCI) of propofol at 4 μg ml^−1^ (induction time, 60 s); muscular relaxation was obtained in both groups with rocuronium 0.6 mg kg^−1^ IV; anesthesia was maintained with remifentanil 0.2 μg kg^−1^ min^−1^ and TCI propofol, adjusted (2–4 μg ml^−1^) to maintain a BIS index below 50; Acetaminophen absorption was used as an indirect measure of gastric emptying; 2. GAS Group: premedication with midazolam 1–2 mg IV; anesthesia was induced with 8% sevoflurane via a facial mask; muscular relaxation was obtained in both groups with rocuronium 0.6 mg kg^−1^ IV; anesthesia was maintained with sevoflurane, with concentrations adjusted to maintain a BIS index below 50; Acetaminophen absorption was used as an indirect measure of gastric emptying
Wang 2024 (a)	Multicentric	China	General Surgery	1. ASA physical status 1–3; 2. Patients undergoing thyroid and parathyroid surgery requiring general anaesthesia	1. Patients with any dyspnoea or tracheal compression; 2. patients with formal diagnosis of obstructive sleep apnoea; biochemical; 3. hyperthyroidism or hypothyroidism (based on biochemistry); 4. left ventricular ejection fraction < 40%; 5. heart rate < 50 beats min^−1^; 6. sick sinus syndrome or second-degree or greater atrioventricular block; 7. ChildPugh-Turcotte class C hepatic dysfunction; 8. need for renal replacement therapy; 9. seizures or epilepsy; chronic pain history; 10. pre-operative use of sedatives or analgesics; 11. pregnancy or breastfeeding; or allergy to medications used in this study	PONV 0–48 h	394	1. OFA Group: propofol 1.52.0 mg kg; intravenous esketamine 0.3 mg kg; intravenous lidocaine 1 mg kg; dexmedetomidine infusion (0.5 µg/kg/h followed by 0.2 µg/kgkg/h); cisatracurium 0.150.2 mg.kg; esketamine.; 2. Opioid Group: propofol 1.52.0 mg kg; intravenous sufentanil 0.3 lg.kg; normal saline volume matched to lidocaine; cisatracurium 0.150.2 mg kg
Ziemann-Gimmel 2014	Monocentric	Uruguay	Bariatric Surgery (endoscopic procedures only)	1. Patients older than 18 years old; 2. Patients undergoing elective bariatric surgery	1. Patients taking high doses of opioids before operation for chronic pain; 2. Patients with allergies to any study medication	Postoperative nausea and vomiting	124	1. Classic (Opioid) Group: fentanyl; sevoflurane or desflurane; morphine or hydromorphone. 2. TIVA (OFA) Group: dexmedetomidine IV; propofol IV
Pal 2023	Monocentric	India	General Surgery (endoscopic procedures only)	1. ASA I, II patients; 2. patients aged from 20 to 60 years; 3. patients scheduled for LC under general anaesthesia	1. Patients with allergy to study medication; 2. history of analgesic dependence and opiate tolerance; 3. epilepsy and psychiatric disturbances; 4. pre-existing diseases like cardiopulmonary diseases, hepatic dysfunction, renal dysfunction, psychiatric illness; 5. pregnancy and lactation	Haemodynamic stability; Postoperative speed and quality of recover	90	1. OBA: midazolam 0.05 mg/kg iv propofol iv fentanyl (2 μg/kg) over 10 min before induction of anaesthesia; fentanyl 0.5 μg/kg was given whenever required till the gall bladder was resected; 2. OFA: midazolam 0.05 mg/kg iv propofol iv lignocaine (2 mg/kg) and dexmedetomidine (0.5 μg/kg) both intravenously over 10 min before induction of anaesthesia; analgesia was maintained by infusion of lignocaine 2 mg/kg/HR and dexmedetomidine 0.5 μg/kg/h
Perez 2024	Monocentric	Uruguay	Bariatric Surgery (endoscopic procedures only)	1. Patients at least 18 years of age; 2. patients undergoing elective bariatric surgery; 3. informed consent; 4. robotic or laparoscopic Roux-en-Y gastric bypass (RYGB) and robotic or laparoscopic sleeve gastrectomy (SG)	1. Chronic opioid use (including any opioid use within the 4 weeks prior to surgery); 2. chronic antiemetic use; 3. hypersensitivity or contraindication to any of the study drugs; 4. inability to provide informed consent; 5. pregnant or lactating patients; 6. inability to provide postoperative pain scores; 7. conversion to open laparotomy	Opioid consumption in the 24 h following surgery	181	1. Group B (OFA): Oral acetaminophen + Gabapentin (preoperative) + Lidocaine 1.5 mg/kg bolus + Propofol 2–3 mg/kg bolus + Dexmedetomidine 1 μg/kg bolus over 10 min + Ketamine 0.5 mg/kg bolus + Dexmedetomidine infusion 0.4 μg/kg/hour (titrated between 0.3 and 0.5 μg/kg/hour) + Lidocaine infusion 2 mg/kg/h + Esmolol boluses (as needed) + Sevoflurane (maintenance) + Neostigmine + Glycopyrrolate or Sugammadex (reversal) + Scopolamine patch + Dexamethasone 4 mg + Haloperidol 1 mg (prophylaxis) + Ondansetron (as needed) 2. Group A (Control): Oral acetaminophen + Gabapentin (preoperative) + Lidocaine 1.5 mg/kg bolus + Propofol 2–3 mg/kg bolus + Fentanyl 50 μg with induction + Additional fentanyl boluses as needed + Sevoflurane (maintenance) + Neostigmine + Glycopyrrolate or Sugammadex (reversal) + Scopolamine patch + Dexamethasone 4 mg + Haloperidol 1 mg (prophylaxis) + Ondansetron (as needed)
Bae 2024	Monocentric	South Korea	General Surgery (endoscopic procedures only)	1. Patients aged > 19 years; 2. patients scheduled to undergo laparoscopic gastrectomy for gastric cancer	1. History of allergic reactions to drugs; 2. history of drug addiction; 3. chronic pain requiring analgesics; 4. cancers other than the stomach cancer; 5. history of hospitalization for psychiatric disorders; 6. history of sleep apnea; 7. preoperative pulse oximetry (SpO_2_) values < 95%; 8. moderate or severe hepatic impairment; 9. hypotension, bradycardia (heart rate [HR] < 50 bpm), atrioventricular block, intraventricular block, or sinus block; 10. body mass index (BMI) > 35 kg/m^2^; 11. blood clotting disorders; 12. cognitive impairment; 13. pregnant or lactating women; 14. those who could not understand the consent form (e.g. patients with low literacy, patients who were foreign-born etc.)	Opioid requirement within 24 h after surgery	120	1. Opioid anesthesia (OA) group: Glycopyrrolate 0.2 mg + Propofol 1.5–2.0 mg/kg + Rocuronium 0.8 mg/kg + Sevoflurane (adjusted to PSI 25–50) + Remifentanil via TCI (Minto model; 3–5 ng/mL during induction, 2–8 ng/mL during surgery) + Nicardipine or Esmolol (as needed for BP/HR control); 2. opioid-free anesthesia (OFA) group: Glycopyrrolate 0.2 mg + Propofol 1.5–2.0 mg/kg + Rocuronium 0.8 mg/kg + Sevoflurane (adjusted to PSI 25–50) + Dexmedetomidine 1 μg/kg (10-min bolus) + Dexmedetomidine infusion 0.2–0.7 μg/kg/h + Lidocaine 1 mg/kg (bolus) + Lidocaine infusion 1 mg/kg/h + Nicardipine or Esmolol (as needed for BP/HR control)
Wang 2024 (b)	Monocentric	China	General Surgery	1. Aged 18–65 years; 2. ASA I–II; 3. absence of significant abnormalities in heart, lung, liver and kidney function	1. Allergic to narcotic drugs; 2. history of long-term opioid use; 3. presence of comorbid mental illness; 4. patients refused	Postoperative assessment of the QoR-40 questionnaire score after 24 h	129	1. OFA Group: premedication: Midazolam. -Induction: esketamine (0.5 mg/kg), propofol (1–2 mg/kg) and cisatracurium (0.2–0.3 mg/kg); Dexmedetedomidine. Manteinance: Sevoflurane and Dexmedetedomidine. 2. OA Group: Premedicatio: Midazolam. -Induction: sufentanyl (0.3–0.4 μg/kg), propofol (1–2 mg/kg), and cisatracurium (0.2–0.3 mg/kg). Maintenance: sufentanyl (0.2 μg/kg/h), cisatracurium (0.1 mg/kg/h) and sevoflurane (0.5–1 MAC)
Chassery 2024	Monocentric	France	Orthopedic Surgery	1. Patients scheduled for daycase total hip replacement 2. Age > 18 3. Informed consent	1. Age < 18; 2. refusal consent; 3. pregnancy; 4. breastfeeding; 5. neuropsychiatric disorder; 6. chronic pain syndrome; 7. patients with contraindications to any medications; 8. contraindications to Dex infusion and to laryngeal mask; 9. patients under the protection of the adults	Total oral morphine equivalent (OME) consumption during the first 24 h after surgery	80	1. OSA group (control group): preoperative: 100 ml normal saline infusion over 30 min was administered and a dose of sufentanil 10 pg, 2 ml iv was injected on induction of anaesthesia. Induction: propofol bolus and ketamine bolus Maintenance: propofol iv; 2. OFA group: Preoperative: Dexmedetomidine 1 µg/kg/h ev. Induction: propofol bolus and ketamine bolus maintenance: propofol iv

Thirteen of the included studies described maintenance regimens for total intravenous anesthesia based on propofol infusion. In contrast, the remaining studies reported the use of inhalational agents: sevoflurane (21 studies), desflurane (6 studies), isoflurane (6 studies) and nitrous oxide (6 studies). Eleven studies described the infiltration of the surgical wound with local anesthesia. All included studies compared two distinct intervention arms.

### Risk of bias

**Fig. 2:**
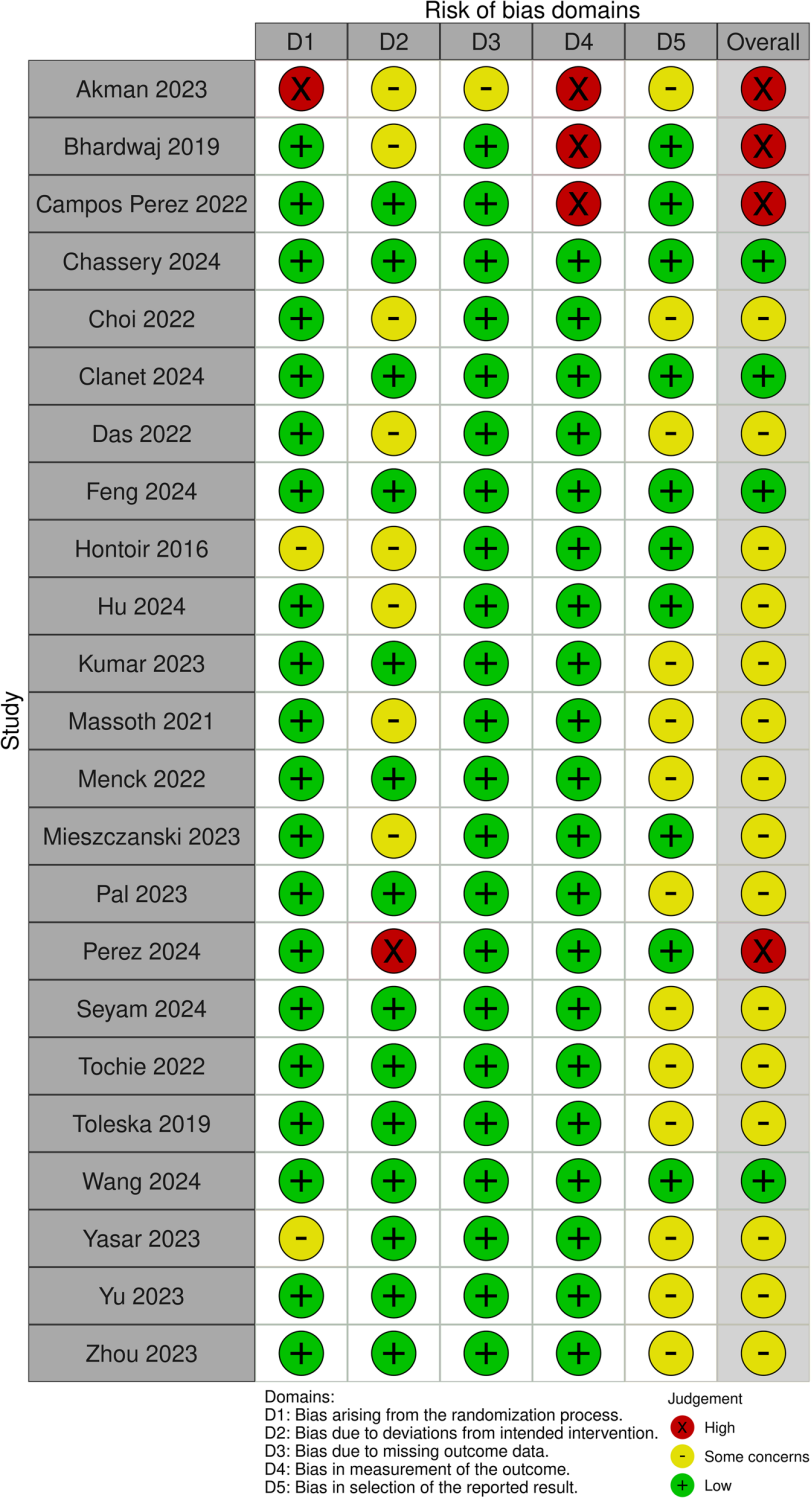
Traffic light plot for Risk of Bias assessment (ROB2) of the primary outcome

Out of the studies reporting the primary outcome, six were classified as low risk of bias, six as high risk and eleven raised some concerns. Among the high-risk studies, two had critical issues with the randomization process, and two reported deviations from intended interventions, and multiple domains in the remaining studies were rated as having some concerns, significantly increasing the overall risk of bias. Figure 2 shows the results of the risk of bias assessment related to the primary outcomes in a graphical format.

We performed the GRADE assessment for each comparison between each OFA regimen and opioid-based anesthesia as a comparator, and we rated seven comparisons with moderate confidence, one with low confidence, and four with very low confidence (Supplementary Fig. 14).

The main reasons for the downgrading of the confidence in our results were within-study risk of bias, reporting bias and, for three studies, statistical heterogeneity (Supplementary Fig. 14).

### Primary outcome

#### Pain intensity at 24 h

**Fig. 3:**
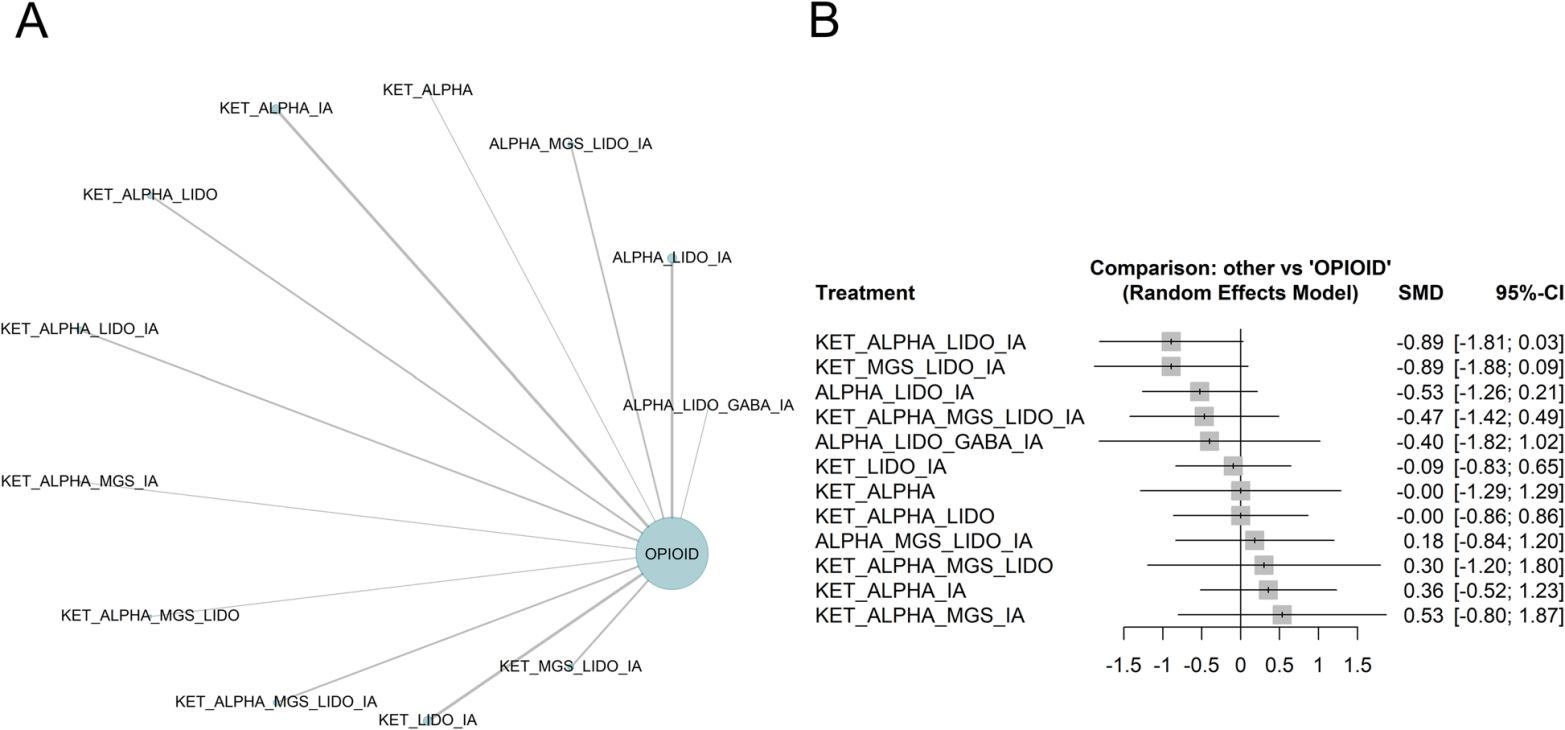
The analysis of the primary outcome: pain intensity at 24 h. **A** Network of interventions with node sizes proportional to the number of patients allocated to the treatments. **B** Forest plot for the comparisons of the different OFA regimens vs opioid based anesthesia. *ALPHA* clonidine or dexmedetomidine, *GABA* gabapentinoids, *IA* inhaled anesthetics, *KET* ketamine, *LIDO* lidocaine, *MGS* magnesium sulfate

Twenty-three studies allocating 2962 patients to 13 different anesthesia regimens were included in the quantitative analysis [6, 27, 29, 30, 32–36, 40, 41, 44, 47–50, 53–55, 59–62]. The primary node of the network is the clustered intervention used as the main comparator, opioid-based anesthesia, with 49% of the patients, followed by alpha agonists, lidocaine and inhaled anesthetics with 470 patients across three studies (Fig. 3A, Supplementary Figs. 2–4). Based on the random-effects frequentist network meta-analysis, the top-ranked treatments were the following combinations: ketamine, alpha agonists, lidocaine, inhaled anesthetics (SUCRA 0.843); ketamine, magnesium sulfate, lidocaine, inhaled anesthetics (SUCRA 0.836); alpha agonists, lidocaine, inhaled anesthetics (SUCRA 0.71, Supplementary Fig. 6). According to the GRADE confidence rating, the comparison of ketamine, alpha agonists, lidocaine and inhaled anesthetics vs. opioid-based anesthesia was graded as “very low” due to within-study bias, reporting bias and heterogeneity (Supplementary Fig. 14).

**Fig. 4:**
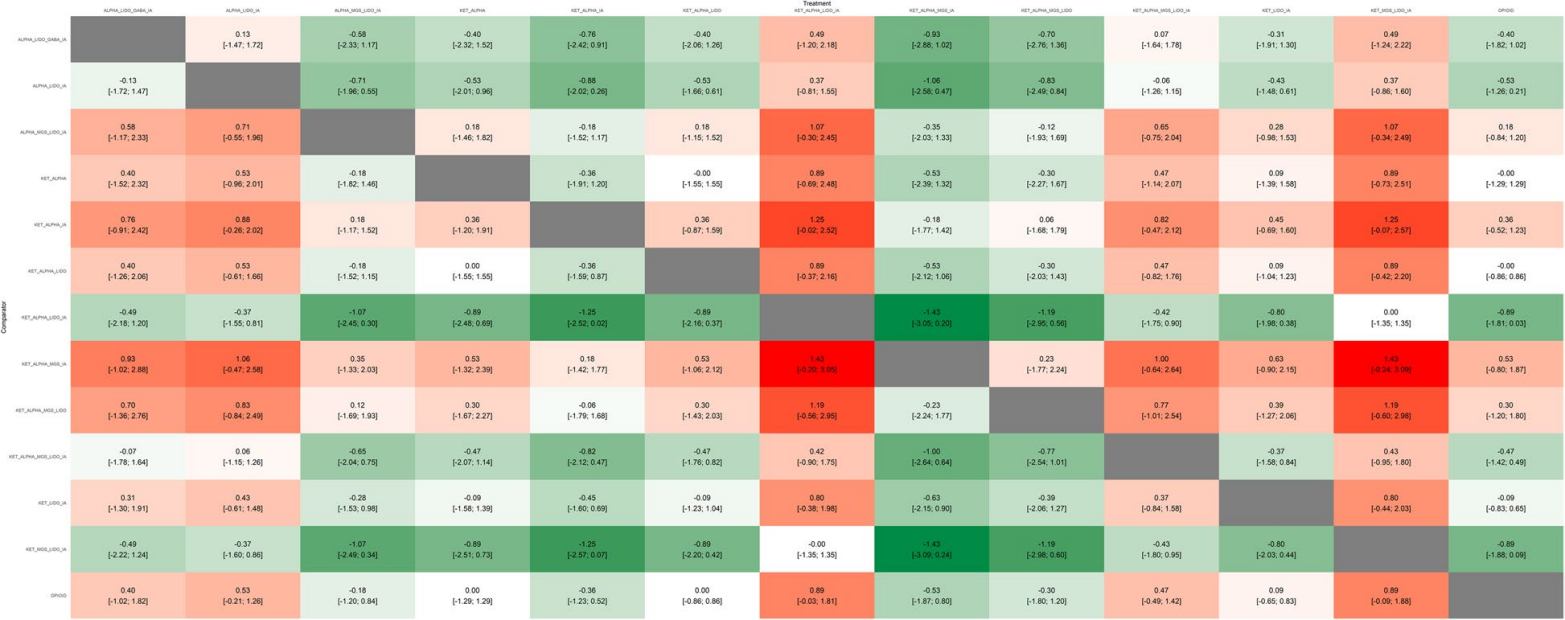
Heatplot containing effect estimates on the primary outcome with confidence intervals for all possible pairwise comparisons. *ALPHA* clonidine or dexmedetomidine, *GABA* gabapentinoids, *IA* inhaled anesthetics, *KET* ketamine, *LIDO* lidocaine, *MGS* magnesium sulfate

None of the studied interventions showed a statistically significant effect (Fig. 3B and Fig. 4). The random-effects pairwise meta-analysis comparing opioid-free and opioid-based regimens showed no difference in pain intensity at 24 h (Supplementary Fig. 8). A sub-analysis excluding the studies judged at high risk of bias showed that the combination of ketamine, alpha agonists, lidocaine and inhaled anesthetics was the best-ranked intervention (SUCRA 0.842), with no statistically significant difference with the opioid comparator (SMD − 0.89, CI − 1.81 to 0.03, Supplementary Figs. 10–13).

### Secondary outcomes

#### Pain intensity at 0–2 h

Eighteen studies reported pain intensity at 0–2 h [6, 27, 32, 35, 40, 43, 44, 47–49, 52, 53, 55, 57, 59–62]. The network of the interventions included 12 different regimens and 2322 patients (Supplementary Figs. 15–18). The top-ranked treatments were alpha agonists, magnesium sulfate, lidocaine and inhaled anesthetics (SUCRA 0.83); ketamine, alpha agonists, magnesium sulfate, lidocaine and inhaled anesthetics (SUCRA 0.74); ketamine, magnesium sulfate and lidocaine, inhaled anesthetics (SUCRA 0.726); and alpha agonists, lidocaine and inhaled anesthetics (SUCRA 0.721, Supplementary Fig. 20).

**Fig. 5:**
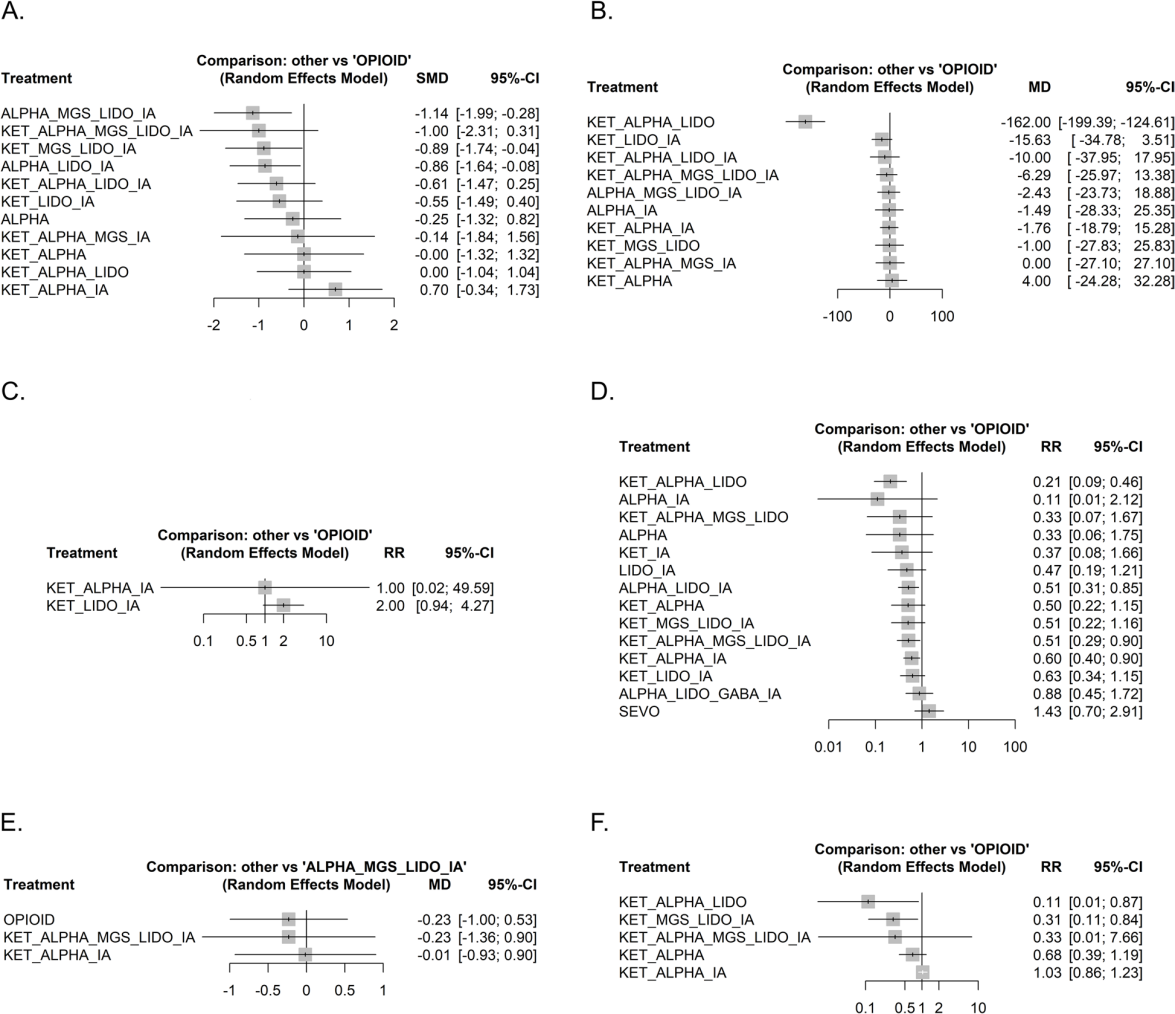
Forest plots for the comparisons of the different OFA regimens vs opioid based anesthesia for the secondary outcomes and selected adverse events. **A** Pain intensity at 0–2 h. **B** Postoperative opioid consumption within 48 h as oral morphine equivalents. **C** Number of postoperative rescue analgesia requests. **D** Incidence of postoperative nausea or vomiting (PONV). **E** Duration of hospital stay. **F** Hypotension. *ALPHA* clonidine or dexmedetomidine, *GABA* gabapentinoids, *IA* inhaled anesthetics, *KET* ketamine, *LIDO* lidocaine, *MGS* magnesium sulfate

The only intervention that showed a significant effect on pain intensity at 0–2 h was the combination of alpha agonists, magnesium sulfate, lidocaine and inhaled anesthetics (SMD − 1.14, CI − 1.99 to − 0.28, Fig. 5A).

The random-effects pairwise meta-analysis comparing opioid-free and opioid-based regimens showed a statistically but not clinically significant reduction of pain at 0–2 h in the OFA group (SMD − 0.42, CI − 0.7 to − 0.14, Supplementary Fig. 23).

Additional outcomes of interest included pain intensity within 2 h following emergence from anesthesia, postoperative opioid consumption within 48 h expressed as oral morphine equivalents, the number of postoperative rescue analgesia requests, the incidence of postoperative nausea or vomiting (PONV), duration of hospital stay and the occurrence of adverse events as reported by the authors.

#### Postoperative opioid consumption within 48 h as oral morphine equivalents

Fifteen studies reported postoperative opioid consumption within 48 h [3, 6, 26, 27, 29, 32, 34, 36, 38, 40, 46–48, 57, 61]. One thousand five hundred and ninety-two patients were allocated to eleven interventions (Supplementary Figs. 25–28).

The top-ranked treatments were ketamine, alpha agonists and lidocaine (SUCRA 1); ketamine, lidocaine and inhaled anesthetics (SUCRA 0.727); ketamine, alpha agonists, lidocaine and inhaled anesthetics (SUCRA 0.586, Supplementary Fig. 30).

Only the combination of ketamine, alpha agonists and lidocaine was associated with a statistically and clinically significant reduction in postoperative opioid consumption (MD − 162 mg, CI − 199.39 to − 124.61 mg, Fig. 5B).

The random-effects pairwise meta-analysis comparing opioid-free and opioid-based regimens showed no difference (MD − 13.79 mg, CI − 36.12 to 8.55 mg, Supplementary Fig. 33).

A single study, Bhardwaj 2019, was a notable outlier with an extreme effect size (MD − 162 mg, − 188.05 to − 135.95 mg), and it heavily affected the results of the NMA but not the pairwise meta-analysis (weight 6.1%).

### Number of postoperative rescue analgesia requests

Five studies reported the number of pain episodes that required the administration of rescue analgesia, allocating 634 patients to three interventions [3, 26, 35, 36, 44] (Supplementary Figs. 35–38). Neither the NMA (Fig. 5C) nor the pairwise meta-analysis showed a statistically significant effect of an opioid-free regimen (Supplementary Fig. 43).

### Incidence of postoperative nausea or vomiting

Twenty-nine studies allocating 3774 patients to 15 interventions reported the proportion of patients experiencing postoperative nausea or vomiting [2, 3, 6, 7, 26, 29–34, 36, 37, 39, 41, 42, 49–52, 54, 56–63] (Supplementary Figs. 45–48). The association of ketamine, alpha agonists, and lidocaine (RR 0.21, CI 0.09 to 0.46; SUCRA 0.822) showed a statistically significant effect on PONV incidence (Fig. 5D).

The pairwise meta-analysis showed a favorable effect of OFA on PONV (RR 0.54, CI 0.43 to 0.68, Supplementary Fig. 53).

### Duration of hospital stay

Four studies reported the duration of the hospital stay and allocated 373 patients to 4 interventions [2, 6, 36, 48, 54] (Supplementary Figs. 55–58). According to SUCRA, the comparator opioid-based anesthesia (OBA) was the best intervention (SUCRA 0.647), followed by the combination of ketamine, alpha agonists, magnesium sulfate, lidocaine and inhaled anesthetics (SUCRA 0.598). No interventions showed a statistically significant effect according to both NMA (Fig. 5E) and pairwise meta-analysis (Supplementary Fig. 62).

### Adverse events and safety

Sixteen studies reported adverse events for each intervention arm [2, 3, 6, 7, 26, 29–34, 36, 37, 39, 41, 42, 49–52, 54, 56–61, 63]. Safety data available from the included studies are limited to the intraoperative and the early postoperative period (Supplementary Table 2, Supplementary Figs. 64–66). The vast majority of cases pertain to hemodynamic variables such as intraoperative hypertension, hypotension, bradycardia and tachycardia. The combinations of ketamine, alpha agonists and lidocaine (RR 0.11, CI 0.01 to 0.87) and ketamine, magnesium, lidocaine and inhaled anesthetics (RR 0.31, CI 0.11 to 0.84) showed a statistically significant reduction in the incidence of hypotension (Fig. 5F).

## Discussion

Our systematic review and meta-analysis showed no statistically significant effect of OFA regimens compared with OBA regimens on pain within 24 h after surgery. The results were in line with a previously published pairwise meta-analysis comparing opioid-free and opioid-based regimens [1]. These data suggest that, despite the theoretical benefits associated with avoiding perioperative opioids, such as reduced opioid-related adverse effects and improved postoperative recovery, current evidence does not support the superior analgesic efficacy of OFA in the immediate postoperative period compared with the use of opioids within 24 h after surgery. However, some combinations of drugs adopted as OFA regimens turned out to be associated with a statistically and clinically significant reduction in postoperative opioid consumption (secondary outcome). This was the case for ketamine, alpha agonists and lidocaine (MD – 162 mg, CI – 199.39 to – 124.61 mg) that also had a statistically significant effect on PONV incidence and was ranked as the best intervention towards the primary outcome (pain at 24 h). The combinations of ketamine, alpha agonists and lidocaine (RR 0.11, CI 0.01 to 0.87) and ketamine, magnesium, lidocaine and inhaled anesthetics (RR 0.31, CI 0.11 to 0.84) also showed a statistically significant reduction in the incidence of hypotension. These results seem to be in contrast with the data regarding the duration of hospital stay, where the comparator (OBA) was the best intervention (SUCRA 0.64). However, these data confirms how difficult it is to impact the length of stay or other critically important clinical outcomes with a single intervention or even with a combination of pharmacological intraoperative interventions. Overall, we found a high rate of publications over time on the topic of opioid free anesthesia, reflecting the great interest from both researchers and physicians. However, research quality remains low, and no multinational multicentric studies have been conducted. The lack of international multicentric studies highlights a research methodology gap and probably networking issues in this research field, providing important insights for both researchers and scientific societies.

The critical issues with most of the published RCTs are highlighted by our GRADE assessment, which downgraded the confidence in our results for the primary outcome to a moderate to very low level.

However, we identified seven recently published meta-analyses on the topic [1, 5, 8, 64–67].

Of note, the pairwise meta-analysis by Frauenknecht et al. included 23 RCTs comparing OFA to opioid-based anesthesia focused on early postoperative pain (2 h) as the primary outcome and found no significant difference.

The systematic review by Bugada et al. restricted the eligible population to patients with cancer to assess the effect of OFA on PONV as the primary outcome, but their literature screening yielded only two suitable studies.

The pairwise meta-analysis by Feenstra et al. included thirty-eight RCTs, of which 30, with a total of 1701 patients, were included in their quantitative synthesis of postoperative pain intensity at 24 h. They estimated the mean difference of pain scores in favor of OFA as – 0.39, CI – 0.59 to – 0.19. This finding was confirmed by neither our NMA non pairwise meta-analysis (SMD – 0.16, CI – 0.34 to 0.03). A notable difference between their work and our NMA lies in the  inclusion of OFA regimens combined with regional anesthesia. This difference offers an important insight, and we can speculate that positive evidence on OFA over the years may not have been attributable only to OFA itself.

Looking at subgroups of patients categories, meta-analysis by Zhang et al. investigated the effect of OFA in patients with gynecological cancer undergoing elective surgery and included 6 RCTs. Their quantitative synthesis showed a favorable effect of OFA on the incidence of PONV (RR 0.52, CI 0.40 to 0.66) and the use of antiemetic drugs (RR 0.64, CI 0.42 to 0.97). Our synthesis confirmed this result in a broader surgical population (RR 0.54, CI 0.43 to 0.68), and it identified a combination of interventions that could be particularly effective in preventing PONV, such as the association of ketamine, alpha agonists, and lidocaine (RR 0.21, CI 0.09 to 0.46; SUCRA 0.822).

A systematic review and meta-analysis by Olausson comprised 1934 patients from 26 RCTs including laparoscopic gynaecological surgery, upper gastrointestinal surgery and breast surgery. This works compared adverse events, postoperative recovery, discharge time from post-anesthesia care unit, postoperative pain, nausea, vomiting and opioid consumption between strict opioid-free and opioid-based general anesthesia. The paper shows that opioid-free anesthesia may significantly reduce adverse postoperative events (OR 0.32, 95% CI 0.22 to 0.46, I^2^ = 56%, *p* < 0.00001), mainly driven by decreased risk of nausea (OR 0.27, (0.17 to 0.42), *p* < 0.00001) and vomiting (OR 0.22 (0.11 to 0.41), *p* < 0.00001). Postoperative opioid consumption was significantly lower in the opioid-free group (− 6.00 mg (− 8.52 to − 3.48), *p* < 0.00001). There was no significant difference in the length of post-anesthesia care unit stay and overall postoperative pain between groups [65].

A pairwise meta-analysis by Liu et al. investigated the effect of OFA with or without regional anesthesia on the perceived quality of recovery measured with the Quality of Recovery-40 Questionnaire (QoR-40) or the Quality of Recovery-15 Questionnaire (QoR-15) and found a clinically significant difference in the QoR-40 score only [66].

A recently published systematic review by Ao et al. compared any OFA regimens with OBA and opioid-sparing anesthesia in adult laparoscopic bariatric surgery, focusing on PONV as the primary outcome [67]. In contrast to our review, they included regimens that involved regional analgesia techniques and did not distinguish between the different drug combinations. This systematic review showed that OFA resulted in less PONV than opioid-sparing regimens, with no differences in postoperative pain and opioid consumption [67]. The incidence of bradycardia was higher in the OFA group compared to the opioid-sparing group [67].

The main limitation of this NMA is given by the quality of the included studies, as evidenced by the presence of risk of bias in multiple domains and by limited external validity. Moreover, we found a profound heterogeneity of the included interventions since both OFA and OBA regimens included a significant variety of drugs, modalities of administration (bolus vs. repeated bolus vs. infusion), and dosage ranges.

Our analysis also has strengths, such as the adoption of rigorous methodology and GRADE assessment, and the decision to exclude regional anesthesia techniques, such as neuraxial and plexus anesthesia, which  allowed us to better isolate the effects of the OFA drugs without the known beneficial effects on pain and opioid consumption associated with these techniques.

The concept of opioid-free anesthesia arose from the need to improve patient safety, facilitate recovery and support public health efforts aimed at preventing the side effects associated with opioid drugs; however, it currently lacks robust evidence regarding its impact on clinical outcomes. Indeed, current evidence does not support the premise that complete intraoperative opioid avoidance is sufficient to improve postoperative outcomes.

Factors such as patient variability, surgical complexity and the need for individualized pain management strategies must be considered when evaluating OFA’s efficacy. Additionally, concerns about the adequacy of alternative analgesic regimens, hemodynamic stability, and the risk of insufficient pain control require further investigation.

Future research should focus on multicentric designs and large-scale RCTs to  generate more robust evidence.

## Conclusions

We have identified a significant heterogeneity in OFA regimens and moderate to high risk of bias in over 70% of studies reporting the primary outcome. No OFA regimen showed a statistically significant effect compared with opioid-based anesthesia on pain within 24 h after surgery. The certainty of evidence for the primary outcome ranges from moderate to very low among the different comparisons. Current evidence does not support the superiority of the analgesic efficacy of OFA in the immediate postoperative period compared with the use of opioids.

## Supplementary Information

Supplementary Material 1: Table 1–2. Figure 1–33.

## Data Availability

The datasets used and/or analyzed during the current study are available from the corresponding author on reasonable request.

## Abbreviations

NMA: Network meta-analysis
OFA: Opioid-free anesthesia
NRS: Numerical rating scale
VAS: Visual analogue scale
VRS: Verbal rating scale
OBA: Opioid-based anesthesia
PONV: Postoperative nausea and vomiting
NMDA: *N*-Methyl-_D_-aspartate
NSAIDs: Non-steroidal anti-inflammatory drugs
PRISMA: Preferred reporting items for systematic reviews and meta-analyses
GRADE: Grading of recommendations, assessment, development and evaluation approach for network meta-analysis
SUCRA: Surface under the cumulative ranking curve
