# Supplementary material for: Effectiveness and safety of opioid-free anesthesia compared to opioid-based anesthesia: a systematic review and network meta-analysis
Source: J Anesth Analg Crit Care. 2025 Aug 13;5:53. doi: 10.1186/s44158-025-00272-9 (PMC12351777; doi:10.1186/s44158-025-00272-9)
Supplement: Supplementary file 1 — Supplementary Material 1: Table 1–2. Figure 1–33. [file 44158_2025_272_MOESM1_ESM.docx]

**Supplementary Material**

**Effectiveness and safety of opioid-free anesthesia compared to opioid-based anesthesia: a systematic review and network meta-analysis**

Vincenzo Francesco Tripodi^§,1^, Salvatore Sardo^§,2^, Mariachiara Ippolito^3,4^, Andrea Cortegiani^3,4^

^§^ These authors contributed equally and should be considered as first authors

^1^ Anesthesia and Intensive Care, Human Pathology Department, University Hospital “Gaetano Martino”, Messina, Italy

^2^ Department of Medical Sciences and Public Health, University of Cagliari, Monserrato, Italy

^3^ Department of Anesthesia, Analgesia, Intensive Care and Emergency. University Hospital Policlinico Paolo Giaccone, Palermo, Italy

^4^ Department of Precision Medicine in Medical, Surgical and Critical Care (Me.Pre.C.C.). University of Palermo, Italy.

**List of contents**

[PRISMA NMA Checklist of Items to Include When Reporting A Systematic Review Involving a Network Meta-analysis 4](#_Toc193215434)

[List of abbreviations 9](#_Toc193215435)

[Supplementary Table 1: Excluded studies with reason 10](#_Toc193215436)

[Supplementary Table 2: Adverse events 14](#_Toc193215437)

[Geographical distribution of the included studies: Supplementary Figure 1 20](#_Toc193215438)

[Pain intensity at 24 hours: Supplementary Figures 2-14 21](#_Toc193215439)

[Supplementary Figure 2: Pain intensity at 24 hours – Network characteristics 21](#_Toc193215440)

[Supplementary Figure 3: Pain intensity at 24 hours – Characteristics of the interventions 22](#_Toc193215441)

[Supplementary Figure 4: Pain intensity at 24 hours – Characteristics of the comparisons 23](#_Toc193215442)

[Supplementary Figure 5: Pain intensity at 24 hours – Nodesplit analysis 24](#_Toc193215443)

[Supplementary Figure 6: Pain intensity at 24 hours – Rankplot 25](#_Toc193215444)

[Supplementary Figure 7: Pain intensity at 24 hours – Results of the individual studies 26](#_Toc193215445)

[Supplementary Figure 8: Pain intensity at 24 hours – Forest plot of the pairwise meta-analysis 27](#_Toc193215446)

[Supplementary Figure 9: Pain intensity at 24 hours – Funnel plot of the pairwise meta-analysis 28](#_Toc193215447)

[Supplementary Figure 10: Pain intensity at 24 hours – Network plot of the ROB2 sub-analysis 29](#_Toc193215448)

[Supplementary Figure 11: Pain intensity at 24 hours – Forest plot of the ROB2 sub-analysis 30](#_Toc193215449)

[Supplementary Figure 12: Pain intensity at 24 hours – Heatplot of the ROB2 sub-analysis 31](#_Toc193215450)

[Supplementary Figure 13: Pain intensity at 24 hours – Rankplot of the ROB2 sub-analysis 32](#_Toc193215451)

[Supplementary Figure 14: Pain intensity at 24 hours – CINeMA assessment 33](#_Toc193215452)

[Pain intensity at 0-2 hours: Supplementary Figures 15-24 34](#_Toc193215453)

[Supplementary Figure 15: Pain intensity at 0-2 hours – Network characteristics 34](#_Toc193215454)

[Supplementary Figure 16: Pain intensity at 0-2 hours – Characteristics of the interventions 35](#_Toc193215455)

[Supplementary Figure 17: Pain intensity at 0-2 hours – Characteristics of the comparisons 36](#_Toc193215456)

[Supplementary Figure 18: Pain intensity at 0-2 hours – Network plot 37](#_Toc193215457)

[Supplementary Figure 19: Pain intensity at 0-2 hours – Heatplot 38](#_Toc193215458)

[Supplementary Figure 20: Pain intensity at 0-2 hours – Rankplot 39](#_Toc193215459)

[Supplementary Figure 21: Pain intensity at 0-2 hours – Nodesplit analysis 40](#_Toc193215460)

[Supplementary Figure 21: Pain intensity at 0-2 hours – Results of the individual studies 41](#_Toc193215461)

[Supplementary Figure 23: Pain intensity at 24 hours – Forest plot of the pairwise meta-analysis 42](#_Toc193215462)

[Postoperative opioid consumption as oral morphine equivalents in milligrams: Supplementary Figures 25-34 44](#_Toc193215463)

[Supplementary Figure 25: Postoperative opioid consumption – Network characteristics 44](#_Toc193215464)

[Supplementary Figure 26: Postoperative opioid consumption – Characteristics of the interventions 45](#_Toc193215465)

[Supplementary Figure 27: Postoperative opioid consumption – Characteristics of the comparisons 46](#_Toc193215466)

[Supplementary Figure 28: Postoperative opioid consumption – Network plot 47](#_Toc193215467)

[Supplementary Figure 29: Postoperative opioid consumption – Heatplot 48](#_Toc193215468)

[Supplementary Figure 30: Postoperative opioid consumption – Rankplot 49](#_Toc193215469)

[Supplementary Figure 31: Postoperative opioid consumption – Nodesplit analysis 50](#_Toc193215470)

[Supplementary Figure 32: Postoperative opioid consumption – Result of the individual studies 51](#_Toc193215471)

[Supplementary Figure 33: Postoperative opioid consumption – Forest plot of the pairwise meta-analysis 52](#_Toc193215472)

[PubMed 88](#_Toc193215473)

[The Cochrane Library for clinical trials in CENTRAL 88](#_Toc193215474)

[Embase via Elsevier 88](#_Toc193215475)

[CINAHL (Cumulative Index to Nursing and Allied Health Literature) via EBSCO 88](#_Toc193215476)

# PRISMA NMA Checklist of Items to Include When Reporting A Systematic Review Involving a Network Meta-analysis

*Hutton B, Salanti G, Caldwell DM, Chaimani A, Schmid CH, Cameron C, Ioannidis JP, Straus S, Thorlund K, Jansen JP, Mulrow C, Catalá-López F, Gøtzsche PC, Dickersin K, Boutron I, Altman DG, Moher D. The PRISMA Extension Statement for Reporting of Systematic Reviews Incorporating Network Meta-analyses of health care Interventions: Checklist and Explanations. Ann Intern Med. 2015;162(11):777-784.*

**PRISMA NMA Checklist of Items to Include When Reporting A Systematic Review Involving a Network Meta-analysis**

| **Section/Topic** | | **Item #** | **Checklist Item** | **Reported on Page #** |
| --- | --- | --- | --- | --- |
| **TITLE** | |  |  |  |
| Title | | 1 | Identify the report as a systematic review *incorporating a network meta-analysis (or related form of meta-analysis).* | Manuscript-Title page |
|  | |  |  |  |
| **ABSTRACT** | |  |  |  |
| Structured summary | | 2 | Provide a structured summary including, as applicable:  **Background:** main objectives  **Methods:** data source, study eligibility criteria, participants, and interventions; study appraisal, and *synthesis methods, such as network meta-analysis.*  **Results:** number of studies and participants identified, summary estimates with corresponding confidence/credible interval, and *treatment rankings may also be discussed. The authors may choose to summarise pairwise comparisons against a chosen treatment included in their analyses for brevity.*  **Discussion/Conclusions:** limitations: conclusions and implications of findings.  **Other:** primary source of funding; systematic review registration number with registry name. | Manuscript-Abstract |
|  | |  |  |  |
| **INTRODUCTION** | |  |  |  |
| Rationale | | 3 | Describe the rationale for the review in the context of what is already known*, including mention of why a network meta-analysis has been conducted.* | Manuscript-Introduction section |
| Objectives | | 4 | Provide an explicit statement of questions being addressed, with reference to participants, interventions, comparisons, outcomes, and study design (PICOS). | Manuscript-Introduction and Methods section |
|  | |  |  |  |
| **METHODS** | |  |  |  |
| Protocol and registration | | 5 | Indicate whether a review protocol exists and if and where it can be accessed (e.g., web address); and, if available, provide registration information, including registration number. | Manuscript-Abstract and Methods section |
| Eligibility criteria | | 6 | Specify study characteristics (e.g., PICOS, length of follow-up) and report characteristics (e.g., years considered, language, publication status) used as criteria for eligibility, giving a rationale. *Clearly describe eligible treatments included in the treatment network, and note whether any have been clustered or merged into the same node (with justification).* | Manuscript-Methods section |
| Information sources | | 7 | Describe all information sources (e.g., databases with dates of coverage, contact with study authors to identify additional studies) in the search and date last searched. | Manuscript-Methods section, Figure 1 |
| Search | | 8 | Present full electronic search strategy for at least one database, including any limits used, so that it could be repeated. | Supplementary Material |
| Study selection | | 9 | State the process for selecting studies (i.e., screening, eligibility, included in systematic review, and, if applicable, included in the meta-analysis). | Manuscript-Methods section |
| Data collection process | | 10 | Describe the method of data extraction from reports (e.g., piloted forms, independently, in duplicate) and any processes for obtaining and confirming data from investigators. | Manuscript-Methods section |
| Data items | | 11 | List and define all variables for which data were sought (e.g., PICOS, funding sources) and any assumptions and simplifications made. | Manuscript-Methods section, Table 1 |
| **Geometry of the network** | | **S1** | Describe methods used to explore the geometry of the treatment network under study and potential biases related to it. This should include how the evidence base has been graphically summarised for presentation, and what characteristics were compiled and used to describe the evidence base to readers. | Manuscript-Methods section |
| Risk of bias within individual studies | | 12 | Describe methods used for assessing risk of bias of individual studies (including specification of whether this was done at the study or outcome level), and how this information is to be used in any data synthesis. | Manuscript-Methods section |
| Summary measures | | 13 | State the principal summary measures (e.g., risk ratio, difference in means). *Also describe the use of additional summary measures assessed, such as treatment rankings and surface under the cumulative ranking curve (SUCRA) values, as well as modified approaches used to present summary findings from meta-analyses.* | Manuscript-Methods section |
| Planned methods of analysis | | 14 | Describe the methods of handling data and combining results of studies for each network meta-analysis. This should include, but not be limited to:   - *Handling of multi-arm trials;* - *Selection of the variance structure;* - *Selection of prior distributions in Bayesian analyses; and* - *Assessment of model fit.* | Manuscript-Methods section |
| **Assessment of Inconsistency** | | **S2** | Describe the statistical methods used to evaluate the agreement of direct and indirect evidence in the treatment network(s) studied. Describe efforts taken to address its presence when found. | Manuscript-Methods section |
| Risk of bias across studies | | 15 | Specify any assessment of the risk of bias that may affect the cumulative evidence (e.g., publication bias, selective reporting within studies). | Manuscript-Methods section |
| Additional analyses | | 16 | Describe methods for additional analyses if done, indicating which were pre-specified. This may include, but not be limited to, the following:   - Sensitivity or subgroup analyses; - Meta-regression analyses; - *Alternative formulations of the treatment network; and* - *Use of alternative prior distributions for bayesian analyses (if applicable).* | Manuscript-Results section |
|  | |  |  |  |
| **RESULTS†** | |  |  |  |
| Study selection | | 17 | Give numbers of studies screened, assessed for eligibility, and included in the review, with reasons for exclusions at each stage, ideally with a flow diagram. | Figure 1 – Supplementary Table 2 |
| **Presentation of network structure** | | **S3** | Provide a network graph of the included studies to enable visualisation of the geometry of the treatment network. | Figures 2A, 4A, 5A, 6A Supplementary Figures 24-35 |
| **Summary of network geometry** | | **S4** | Provide a brief overview of characteristics of the treatment network. This may include commentary on the abundance of trials and randomized patients for the different interventions and pairwise comparisons in the network, gaps of evidence in the treatment network, and potential biases reflected by the network structure. | Manuscript-Results section, Supplementary Figures 2-4, 15-18, 25-28, 35-38, 45-48, 55-58 |
| Study characteristics | | 18 | For each study, present characteristics for which data were extracted (e.g., study size, PICOS, follow-up period) and provide the citations. | Table 1, Supplementary Tables 2, 3, References |
| Risk of bias within studies | | 19 | Present data on risk of bias of each study and, if available, any outcome level assessment. | Manuscript-Results section, Figure 2, Supplementary Figure 14 |
| Results of individual studies | | 20 | For all outcomes considered (benefits or harms), present, for each study: 1) simple summary data for each intervention group, and 2) effect estimates and confidence intervals. *Modified approaches may be needed to deal with information from larger networks.* | Supplementary Figures 7, 21, 32, 42, 52, 61 |
| Synthesis of results | | 21 | Present results of each meta-analysis done, including confidence/credible intervals. *In larger networks, authors may focus on comparisons versus a particular comparator (e.g. placebo or standard care), with full findings presented in an Supplementary Material. League tables and forest plots may be considered to summarize pairwise comparisons.* If additional summary measures were explored (such as treatment rankings), these should also be presented. | Figures 3, 4, 5 Supplementary Figures 6, 19-20, 29-30, 39-40, 49-50, 59-60, 64-66 |
| **Exploration for inconsistency** | | **S5** | Describe results from investigations of inconsistency. This may include such information as measures of model fit to compare consistency and inconsistency models, *P* values from statistical tests, or summary of inconsistency estimates from different parts of the treatment network. | Supplementary Figures 5, 21, 31, 41, 51, 61 |
| Risk of bias across studies | | 22 | Present results of any assessment of risk of bias across studies for the evidence base being studied. | Figure 2 |
| Results of additional analyses | | 23 | Give results of additional analyses, if done (e.g., sensitivity or subgroup analyses, meta-regression analyses*, alternative network geometries studied, alternative choice of prior distributions for Bayesian analyses,* and so forth). | ROB2 sub-analysis Supplementary Figures 10-13  Pairwise Meta-analysis Supplementary Figures 8-9, 23-24, 33-34, 43-44, 53-54, 62-63 |
|  | |  |  |  |
| **DISCUSSION** | |  |  |  |
| Summary of evidence | | 24 | Summarize the main findings, including the strength of evidence for each main outcome; consider their relevance to key groups (e.g., healthcare providers, users, and policy-makers). | Manuscript-Conclusions section |
| Limitations | | 25 | Discuss limitations at study and outcome level (e.g., risk of bias), and at review level (e.g., incomplete retrieval of identified research, reporting bias). *Comment on the validity of the assumptions, such as transitivity and consistency. Comment on any concerns regarding network geometry (e.g., avoidance of certain comparisons).* | Manuscript-Discussion section |
| Conclusions | | 26 | Provide a general interpretation of the results in the context of other evidence, and implications for future research. | Manuscript-Conclusions section |
|  | |  |  |  |
| **FUNDING** | |  |  |  |
| Funding | 27 | Describe sources of funding for the systematic review and other support (e.g., supply of data); role of funders for the systematic review. This should also include information regarding whether funding has been received from manufacturers of treatments in the network and/or whether some of the authors are content experts with professional conflicts of interest that could affect use of treatments in the network. | | Manuscript-Funding section |

# List of abbreviations

- **ALPHA**, Clonidine/Dexmedetomidine
- **CLON**, Clonidine
- **CORT**, Corticosteroid
- **DIAZ**, Diazepam
- **DES**, Desflurane
- **DEXM**, Dexmedetomidine
- **FOSPROP**, Fospropofol
- **GABA,** Propofol
- **HR,** heart rate
- **IA**, Inhaled Anesthetics
- **ISO**, Isoflurane
- **KET**, Ketamine
- **LIDO**, Lidocaine
- **MGS**, Magnesium sulfate
- **MID**, Midazolam
- **N2O**, Nitrous oxide
- **NSAID**, Non-Steroidal Anti-Inflammatory Drug(s)
- **OPIOID**, Opioid analgesia
- **PCA,** Patient-controlled Analgesia
- **PROP**, Propofol
- **REM**, Remimazolam
- **SEVO**, Sevoflurane
- **THIO**, Thiopental

# Supplementary Table 1: Excluded studies with reason

| Title | Author | Year | Reason for exclusion |
| --- | --- | --- | --- |
| The effect of opioid free versus opioid based anaesthesia on breast cancer pain score and immune response | Aboalsoud | 2021 | Irrelevant intervention (regional anesthesia associated with opioid-free anesthesia) |
| Opioid-free anesthesia compared to opioid anesthesia for laparoscopic radical colectomy with pain threshold index monitoring: a randomized controlled study | An | 2022 | Irrelevant intervention (regional anesthesia associated with opioid-free anesthesia) |
| A Comparison of Analgesic and Recovery Profiles of Ketamine, Lignocaine, and Dexmedetomidine (KeLiDex) Versus Fentanyl-Based Anesthesia in Laparoscopic Nephrectomies: A Randomized, Single-Blind, Pilot Study. | Arun | 2024 | Irrelevant intervention (regional anesthesia associated with opioid-free anesthesia) |
| Anestesia venosa total livre de opioides, com infusões de propofol, dexmedetomidina e lidocaína para colecistectomia laparoscópica: estudo prospectivo, randomizado e duplo-cego | Bakan | 2015 | Non English language |
| Effects of opioid-free anaesthesia compared with balanced general anaesthesia on nausea and vomiting after video-assisted thoracoscopic surgery: a single-centre randomised controlled trial | Bao | 2024 | Irrelevant intervention (regional anesthesia associated with opioid-free anesthesia) |
| Opioid free anaesthesia technique in patients undergoing laparotomy for gynaecological malignancy: a randomized controlled trial | Bhoi | 2021 | Conference proceeding or abstract-only report |
| Application of opioid-free general anesthesia for gynecological laparoscopic surgery under ERAS protocol: a non-inferiority randomized controlled trial | Chen | 2023 | Irrelevant intervention (regional anesthesia associated with opioid-free anesthesia) |
| Opioid-Free Using Ketamine versus Opioid-Sparing Anesthesia during the Intraoperative Period in Video-Assisted Thoracoscopic Surgery: A Randomized Controlled Trial | Choi | 2024 | Irrelevant intervention (regional anesthesia associated with opioid-free anesthesia) |
| The perioperative analgesic effect of opioid free anesthesia using combination of dexmedetomidine, ketamine and lidocaine in adolescent patients undergoing Scoliosis Surgery; A randomized Controlled Trial | Elahwal | 2023 | Irrelevant population (pediatric patients) |
| Multimodal Opioid-Free Anesthesia Versus Opioid-Based Anesthesia for Patients Undergoing Cardiac Valve Surgeries: A Randomized Controlled Trial | Elgendy | 2024 | Irrelevant surgical setting (cardiac surgery) |
| Esketamine opioid-free intravenous anesthesia versus opioid intravenous anesthesia in spontaneous ventilation videoassisted thoracic surgery: a randomized controlled trial | Fan | 2023 | Irrelevant intervention (regional anesthesia associated with opioid-free anesthesia) |
| Opioid free anaesthesia improves postoperative quality of recovery after surgery for elective lumbar spine surgeries-a prospective, randomized, comparative study | Gayatri | 2020 | Conference proceeding or abstract-only report |
| Efficiency of opioid-free anesthesia (OFA) in maxillofacial surgery | Guibla | 2021 | Conference proceeding or abstract-only report |
| Efficacy of opioid free anesthesia in mastectomy for breast cancer | Guibla | 2021 | Conference proceeding or abstract-only report |
| Comparison of the Efficacy of Opioid-Free Anesthesia With Conventional Opioid-Based Anesthesia for Nasal Surgeries - A Prospective Randomized Parallel Arm Triple-Blinded Study. | Hariharan | 2023 | Irrelevant intervention (regional anesthesia associated with opioid-free anesthesia) |
| Multimodal nonopioid pain protocol provides equivalent pain control versus opioids following arthroscopic shoulder labral surgery: a prospective randomized controlled trial | Jildeh | 2021 | Irrelevant intervention (non-opioid-free anesthesia) |
| Patient Comfort During Postop Period in Breast Cancer Surgeries: A Randomized Controlled Trial Comparing Opioid and Opioid-Free Anesthesia | Krishnasamy Yuvaraj | 2023 | Irrelevant intervention (regional anesthesia associated with opioid-free anesthesia) |
| A trial of opioid-free anesthesia for major or intermediate non-cardiac surgery | Laviolle | 2021 | Conference proceeding or abstract-only report |
| Opioid-free Anesthesia Protocol on the Early Quality of Recovery after Major Surgery (SOFA Trial): A Randomized Clinical Trial | Léger | 2024 | Irrelevant intervention (regional anesthesia associated with opioid-free anesthesia) |
| Assessing the clinical advantage of opioidreduced anesthesia in thoracoscopic sympathectomy: a prospective randomized controlled trial | Minqiang | 2024 | Irrelevant intervention (regional anesthesia associated with opioid-free anesthesia) |
| Justification of the expediency of using harmless analgesia in bariatric surgery: prospective randomized study | Neimark | 2023 | Non English language |
| Justification of the expediency of using harmless analgesia in bariatric surgery: prospective randomized study | Neimark | 2023 | Non English language |
| [SNACC-510] Comparison of Effects of Opioid Based Anaesthesia and Opioid Free Anaesthesia on Intraoperative Hemodynamics and Postoperative Analgesia in Patients Undergoing Spine Surgery | Panda | 2021 | Conference proceeding or abstract-only report |
| Comparison of analgesia nociception index, surgical pleth index and hemodynamic parameters between patients receiving fentanyl versus dexmedetomidine analgesia for supratentorial craniotomy - an open label active-controlled randomized trial. | Rakesh | 2024 | Observational study |
| Opioids and premature biochemical recurrence of prostate cancer: a randomised prospective clinical trial | Rangel | 2021 | Irrelevant intervention (regional anesthesia associated with opioid-free anesthesia) |
| Comparative Study between Opioid-Free General Anesthesia by Dexmedetomidine and Opioid-Based General Anesthesia in Rhinoplasty Surgeries | Rashad | 2021 | Conference proceeding or abstract-only report |
| Opioid-Free Forefoot Surgery vs Traditional Perioperative Opiate Regimen: A Randomized Controlled Trial | Rooney | 2023 | Irrelevant intervention (regional anesthesia associated with opioid-free anesthesia) |
| Opioid free versus opioid balanced anesthesia in middle ear surgery | Sabry | 2021 | Conference proceeding or abstract-only report |
| To compare the efficacy of opioid free general anesthesia with opioid based general anesthesia on post-operative morphine consumption in patients undergoing breast cancer surgery: a prospective randomized control study | Sarma | 2021 | Conference proceeding or abstract-only report |
| Opioid-Free Analgesia is Safe and Effective in Anterior Cervical Spine Surgery A Randomized Controlled Trial | Segebarth | 2024 | Conference proceeding or abstract-only report |
| OPIOID SPARING & SUPPRESSION OF HEMODYNAMIC RESPONSE BY PREOPERATIVE MULTIMODAL NON-OPIOID ANALGESIC REGIME IN LAPAROSCOPIC SURGERIES UNDER GENERAL ANAESTHESIA: A PROSPECTIVE RANDOMIZED COMPARATIVE STUDY | Srinivasan | 2024 | Irrelevant intervention (regional anesthesia associated with opioid-free anesthesia) |
| An Opioid-Sparing Pain Protocol of Intravenous and Oral Ketorolac Reduces Opioid Consumption and Pain Levels After Arthroscopic Meniscus Surgery: A Prospective, Randomized Controlled Trial | Strony | 2024 | Irrelevant intervention (opioid sparing anesthesia) |
| Opioid-free anesthesia for breast cancer surgery: An observational study | Swagata Tripathy | 2018 | Irrelevant intervention (regional anesthesia associated with opioid-free anesthesia) |
| Opioid-free anesthesia reduces the severity of acute postoperative motion-induced pain and patient-controlled epidural analgesia-related adverse events in lung surgery: randomized clinical trial | Wang S. | 2023 | Irrelevant intervention (regional anesthesia associated with opioid-free anesthesia) |
| Opioid-free anesthesia with esketaminedexmedetomidine versus opioid-based anesthesia with propofol-remifentanil in shoulder arthroscopy: a randomized controlled trial | Xue | 2024 | Irrelevant intervention (regional anesthesia associated with opioid-free anesthesia) |
| Effects of opioid-free anaesthesia on postoperative nausea and vomiting in patients undergoing video-assisted thoracoscopic surgery (OFA-PONV trial): study protocol for a randomised controlled trial | Yan X. | 2023 | Study protocol without reported results |

# Supplementary Table 2: Adverse events

| Study | Treatment | Sample | | Adverse events (number of events) | |
| --- | --- | --- | --- | --- | --- |
| Aboelela 2021 | PROP bolus SEVO OPIOID | | 34 | | 1. respiratory depression (5) 2. PONV (16) 3. ileus (8) 4. constipation (10) |
| Aboelela 2021 | PROP bolus KET bolus LIDO infusion SEVO | | 34 | | 1. respiratory depression (1) 2. PONV (4) 3. ileus (1) 4. constipation (3) |
| Beloeil 2021 | PROP bolus KET infusion DEXM infusion CORT DES | | 157 | | 1. hypertension (125) 2. hypotension (97) 3. bradycardia (30) 4. bradycardia with heart rate < 45 beats/min (25) 5. other severe unexpected events (5) |
| Beloeil 2021 | PROP bolus KET infusion CORT DES OPIOID | | 157 | | 1. hypertension (117) 2. hypotension (94) 3. bradycardia (14) 4. bradycardia with heart rate < 45 beats/min (9) 5. other severe unexpected events (5) |
| Bhardwaj 2019 | PROP infusion OPIOID | | 40 | | No adverse event was reported |
| Bhardwaj 2019 | PROP infusion KET bolus DEXM infusion LIDO infusion | | 40 | | No adverse event was reported |
| Campos-Perez 2022 | PROP infusion KET infusion MGS infusion LIDO infusion OPIOID | | 20 | | No adverse event was reported |
| Campos-Perez 2022 | PROP infusion KET infusion DEXM infusion MGS infusion LIDO infusion | | 20 | | No adverse event was reported |
| Cha 2023 | PROP bolus SEVO OPIOID | | 45 | | No adverse event was reported |
| Cha 2023 | PROP bolus LIDO bolus SEVO | | 45 | | No adverse event was reported |
| Chassery 2024 | PROP infusion KET bolus OPIOID | | 40 | | 1. bradycardia (6) 2. hypotension (19) 3. hypertension (17) 4. prolonged hospitalization (4) 5. bleeding (0) 6. laryngospasm (1) 7. voiding difficulties (0) 8. drowsiness (1) 9. dizziness (8) |
| Chassery 2024 | PROP infusion KET bolus DEXM infusion | | 40 | | 1. bradycardia (9) 2. hypotension (13) 3. hypertension (16) 4. prolonged hospitalization (0) 5. bleeding (1) 6. laryngospasm (0) 7. voiding difficulties (2) 8. drowsiness (1) 9. dizziness (2) |
| Choi 2022 | PROP bolus DEXM infusion LIDO infusion DES | | 37 | | 1. shivering (6) 2. pruritus (0) 3. PCA stopped due to side effects (2) |
| Choi 2022 | PROP bolus DES OPIOID | | 38 | | 1. shivering (6) 2. pruritus (1) 3. PCA stopped due to side effects (5) |
| Clanet 2024 | PROP bolus KET infusion DEXM infusion MGS infusion LIDO infusion SEVO | | 86 | | bradycardia requiring atropine administration (2) |
| Clanet 2024 | PROP bolus KET bolus LIDO bolus SEVO OPIOID | | 86 | | bradycardia requiring atropine administration (5) |
| Feng 2024 | PROP bolus KET repeated boli DEXM infusion SEVO | | 60 | | 1. hypotension (23) 2. bradycardia (1) 3. hypertension (16) 4. tachycardia (6) 5. interventions for hemodynamic events (24) 6. sedation in pacu (5) 7. headache or dizziness 0-48 h (8) 8. nightmare or hallucination 0-48 h (3) |
| Feng 2024 | PROP bolus SEVO OPIOID | | 60 | | 1. hypotension (19) 2. bradycardia (3) 3. hypertension (13) 4. tachycardia (2) 5. interventions for hemodynamic events (19) 6. sedation in pacu (1) 7. headache or dizziness 0-48 h (9) 8. nightmare or hallucination 0-48 h (2) |
| Goyal 2017 | PROP bolus NSAID DES N2O OPIOID | | 30 | | No adverse event was reported |
| Goyal 2017 | PROP bolus DEXM infusion NSAID DES N2O | | 30 | | No adverse event was reported |
| Hao 2023 | PROP infusion KET infusion DEXM infusion SEVO | | 40 | | 1. nausea (9) 2. vomiting (6) 3. constipation (2) 4. difficulty passing urine (1) 5. difficulty concentrating (3) 6. drowsiness/difficulty staying awake (4) 7. feeling lightheaded or dizzy (4) 8. feeling confused (3) 9. feelings of general fatigue or weakness (8) 10. itchiness (1) 11. xerostomia (4) 12. headache (2) |
| Hao 2023 | PROP bolus DEXM bolus DEXM infusion SEVO OPIOID | | 40 | | 1. nausea (19) 2. vomiting (17) 3. constipation (4) 4. difficulty passing urine (3) 5. difficulty concentrating (9) 6. drowsiness/difficulty staying awake (14) 7. feeling lightheaded or dizzy (12) 8. feeling confused (5) 9. feelings of general fatigue or weakness (18) 10. itchiness (3) 11. xerostomia (13) 12. headache (8) |
| Hu 2024 | PROP bolus SEVO OPIOID | | 36 | | No adverse event was reported |
| Hu 2024 | PROP bolus KET infusion LIDO infusion SEVO | | 36 | | No adverse event was reported |
| Jose 2023 | PROP infusion LIDO bolus N2O OPIOID | | 60 | | 1. vomiting (4) 2. bradycardia (0) 3. agitation (1) |
| Jose 2023 | PROP infusion DEXM infusion LIDO infusion N2O | | 60 | | 1. vomiting (0) 2. bradycardia (2) 3. agitation (0) |
| Mansour 2013 | PROP bolus KET infusion NSAID SEVO | | 13 | | No adverse event was reported |
| Mansour 2013 | DEXM bolus NSAID SEVO OPIOID | | 15 | | No adverse event was reported |
| Massoth 2021 | PROP bolus KET infusion DEXM infusion SEVO | | 79 | | 1. nausea (52) 2. vomiting (28) 3. sedation at pacu admission (44) 4. sedation at 15 minutes after pacu admission (22) 5. sedation at 30 minutes after pacu admission (7) 6. explicit recall of preoperative positioning (1) 7. PCA discontinuation (2) 8. persistent bradycardia (1) |
| Massoth 2021 | PROP bolus SEVO OPIOID | | 78 | | 1. nausea (53) 2. vomiting (31) 3. sedation at pacu admission (11) 4. sedation at 15 minutes after pacu admission (4) 5. sedation at 30 minutes after pacu admission (1) 6. explicit recall of preoperative positioning (0) 7. PCA discontinuation (1) 8. persistent bradycardia (0) |
| Pal 2023 | PROP MID CORT ISO N2O OPIOID | | 45 | | No adverse event was reported |
| Pal 2023 | PROP bolus MID DEXM infusion LIDO infusion CORT ISO N2O | | 45 | | No adverse event was reported |
| Perez 2024 | PROP bolus DEXM infusion LIDO infusion GABA CORT SEVO | | 95 | | 1. bradycardia (lowest HR <60 bpm): 54 2. severe bradycardia (lowest HR <50 bpm): 17 3. received phenylephrine (): 67 4. received ephedrine (): 54 5. received vasopressin (): 3 6. received ketorolac ly: 9 |
| Perez 2024 | PROP bolus LIDO bolus GABA CORT SEVO OPIOID | | 86 | | 1. bradycardia (lowest HR <60 bpm): 34 2. severe bradycardia (lowest HR <50 bpm): 10 3. received phenylephrine (): 41 4. received ephedrine (): 28 5. received vasopressin (): 3 6. received ketorolac ly: 20 |
| Rani 2024 | PROP infusion N2O OPIOID | | 30 | | 1. hypertension (>20% baseline map): 10 2. rescue drug for hypertension (esmolol): 10 3. hypotension: 13 4. rescue drug for hypotension (phenylephrine): 13 |
| Rani 2024 | PROP infusion KET infusion N2O | | 30 | | 1. hypertension (>20% baseline map): 18 2. rescue drug for hypertension (esmolol): 18 3. hypotension: 5 4. rescue drug for hypotension (phenylephrine): 2 |
| Saravanaperumal 2022 | PROP infusion DEXM repeated boli | | 33 | | No adverse event was reported |
| Saravanaperumal 2022 | PROP infusion OPIOID | | 33 | | No adverse event was reported |
| Tochie 2022 | PROP bolus KET infusion CLON infusion MGS infusion LIDO infusion CORT ISO | | 18 | | 1. pruritus (0) 2. need for oxygen ly (1) 3. need of reintubation (0) 4. ileus (1) 5. nausea and vomiting (2) 6. bradycardia (0) 7. hypotension (0) 8. tachycardia (0) 9. hypertension (0) |
| Tochie 2022 | PROP bolus DIAZ ISO OPIOID | | 18 | | 1. pruritus (6) 2. need for oxygen ly (12) 3. need of reintubation (0) 4. ileus (15) 5. nausea and vomiting (9) 6. bradycardia (0) 7. hypotension (1) 8. tachycardia (4) 9. hypertension (0) |
| Toleska (b) 2022 | PROP bolus MID SEVO OPIOID | | 40 | | No adverse event was reported |
| Toleska (b) 2022 | PROP bolus KET bolus MID MGS infusion LIDO infusion CORT SEVO | | 40 | | No adverse event was reported |
| Van Loocke 2022 | PROP bolus KET infusion DEXM infusion MGS bolus LIDO infusion SEVO | | 21 | | No adverse event was reported |
| Van Loocke 2022 | PROP bolus SEVO OPIOID | | 19 | | No adverse event was reported |
| Wallden 2006 | PROP infusion OPIOID | | 24 | | No adverse event was reported |
| Wallden 2006 | MID SEVO | | 21 | | No adverse event was reported |
| Wang 2024 (a) | PROP infusion KET bolus DEXM bolus LIDO bolus | | 197 | | 1. need for rescue anti-emetics (2) 2. need for rescue analgesics (1) 3. hypotension with intervention (1) 4. bradycardia with intervention (3) 5. hypertension with intervention (11) 6. tachycardia with intervention (9) 7. desaturation after extubation (1) 8. dizziness or headache (41) 9. nightmare or hallucination (7) |
| Wang 2024 (a) | PROP infusion OPIOID | | 197 | | 1. need for rescue anti-emetics (18) 2. need for rescue analgesics (7) 3. hypotension with intervention (9) 4. bradycardia with intervention (10) 5. hypertension with intervention (6) 6. tachycardia with intervention (5) 7. desaturation after extubation (9) 8. dizziness or headache (39) 9. nightmare or hallucination (4) |
| Wang 2024 (b) | PROP bolus KET infusion MID DEXM bolus DEXM infusion SEVO | | 64 | | 1. need of vasoactive drug (5) 2. respiratory depression (0) 3. delirium (1) |
| Wang 2024 (b) | PROP infusion MID SEVO OPIOID | | 56 | | 1. need of vasoactive drug (15 2. respiratory depression (6) 3. delirium (0) |
| Yasar 2023 | PROP bolus MID LIDO bolus CORT NSAID SEVO OPIOID | | 32 | | 1. bradycardia (5) 2. tachycardia (19) 3. hypotension (13) 4. hypertension (14) 5. inability to urinate (2) 6. pruritus (1) 7. headache (2) 8. spo2 <94% (with 6 lt/min o2 mask) (4) 9. obstructive breathing (2) |
| Yasar 2023 | PROP bolus KET bolus MID MGS infusion LIDO bolus CORT SEVO | | 32 | | 1. bradycardia (2) 2. tachycardia (17) 3. hypotension (4) 4. hypertension (18) 5. inability to urinate (0) 6. pruritus (0) 7. headache (1) 8. spo2 <94% (with 6 lt/min o2 mask) (5) 9. obstructive breathing (0) |
| Yu 2023 | PROP infusion KET bolus LIDO infusion SEVO | | 75 | | No adverse event was reported |
| Yu 2023 | PROP infusion SEVO OPIOID | | 75 | | No adverse event was reported |
| Zhou 2023 | PROP infusion MID DEXM infusion LIDO infusion CORT SEVO | | 388 | | 1. bradycardia (use of atropine) (26) 2. tachycardia (use of esmolol) (34) 3. hypertension (use of vasodilator) (46) 4. intra-operative hypotension (use of vasopressor) (41) |
| Zhou 2023 | PROP infusion MID CORT SEVO OPIOID | | 385 | | 1. bradycardia (use of atropine) (65) 2. tachycardia (use of esmolol) (35) 3. hypertension (use of vasodilator) (32) 4. intra-operative hypotension (use of vasopressor) (43) |
| Ziemann-Gimmel 2014 | PROP bolus CORT SEVO DES OPIOID | | 59 | | No adverse event was reported |
| Ziemann-Gimmel 2014 | PROP infusion KET bolus DEXM infusion CORT | | 60 | | No adverse event was reported |

# Geographical distribution of the included studies: Supplementary Figure 1


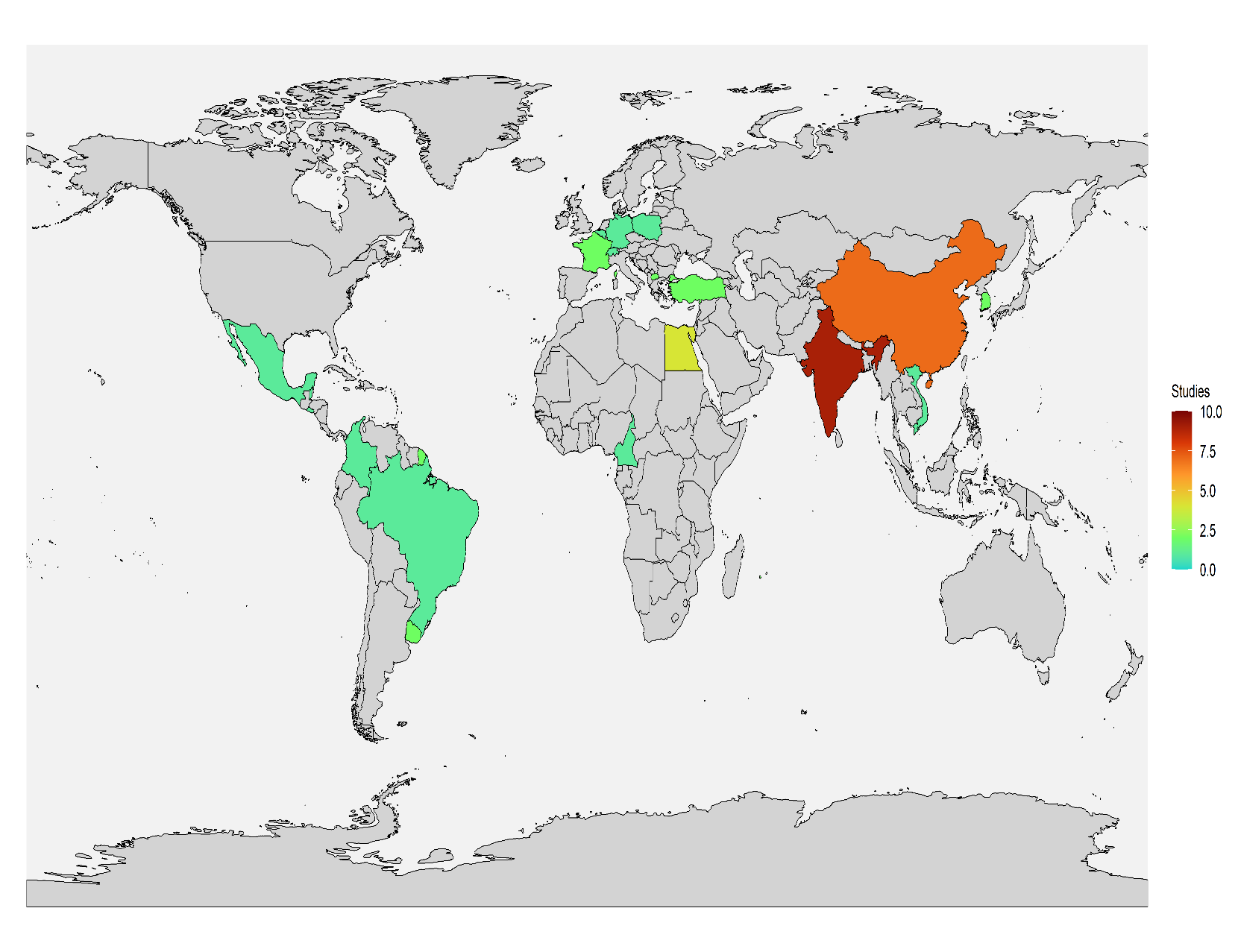


Annicchiarico L (2025). _WorldMapR: Worldwide or Coordinates-Based Heat Maps_. R package version 1.1.0,

<https://CRAN.R-project.org/package=WorldMapR>.

# Pain intensity at 24 hours: Supplementary Figures 2-14

## Supplementary Figure 2: Pain intensity at 24 hours – Network characteristics


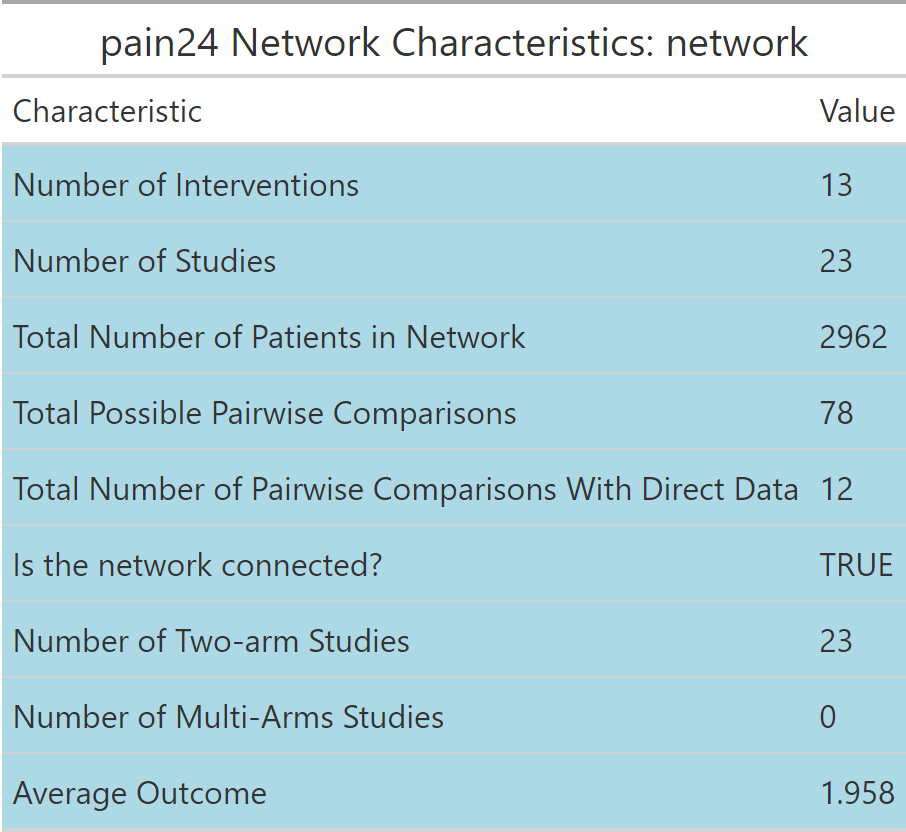


.

## Supplementary Figure 3: Pain intensity at 24 hours – Characteristics of the interventions


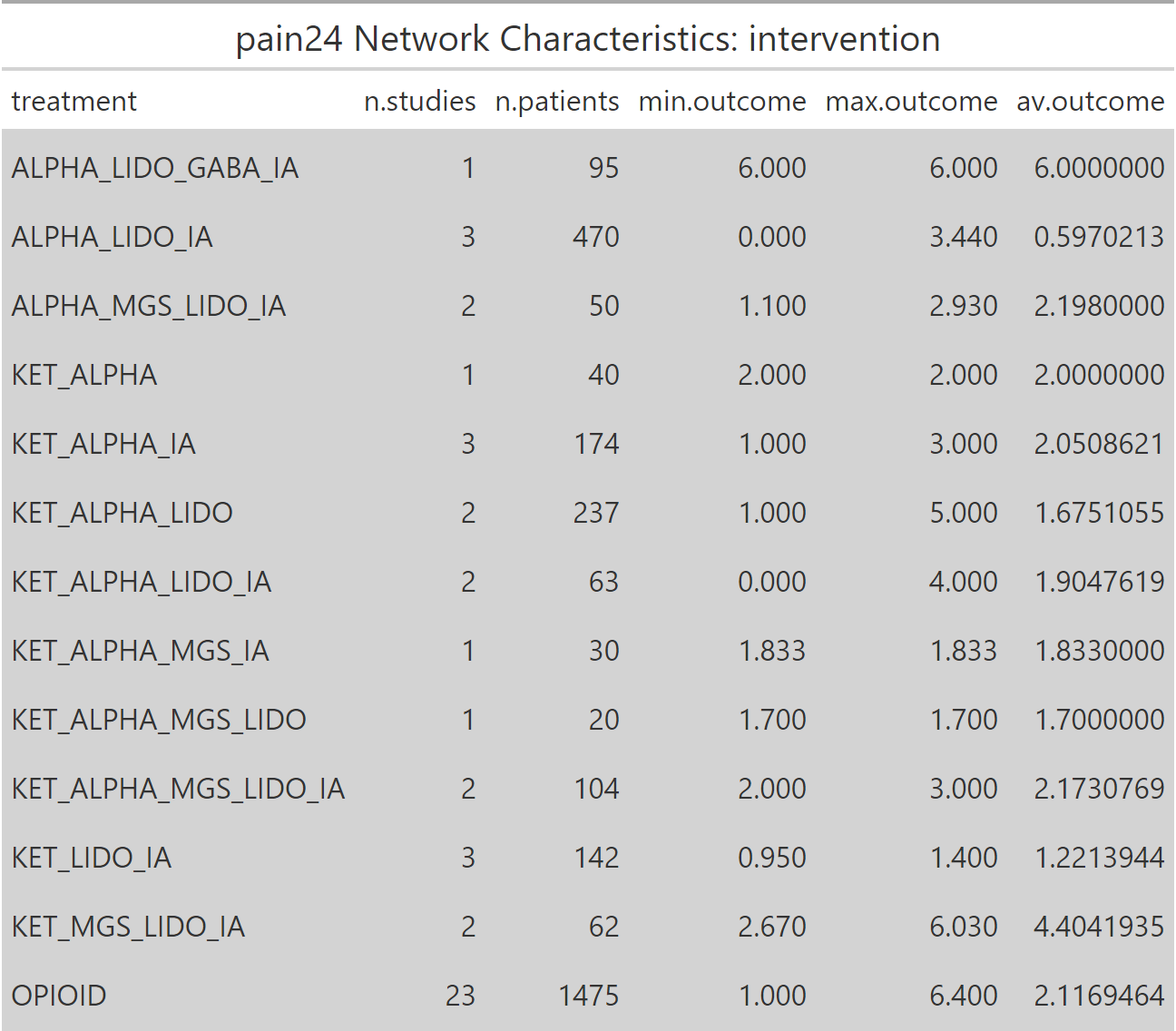


## Supplementary Figure 4: Pain intensity at 24 hours – Characteristics of the comparisons


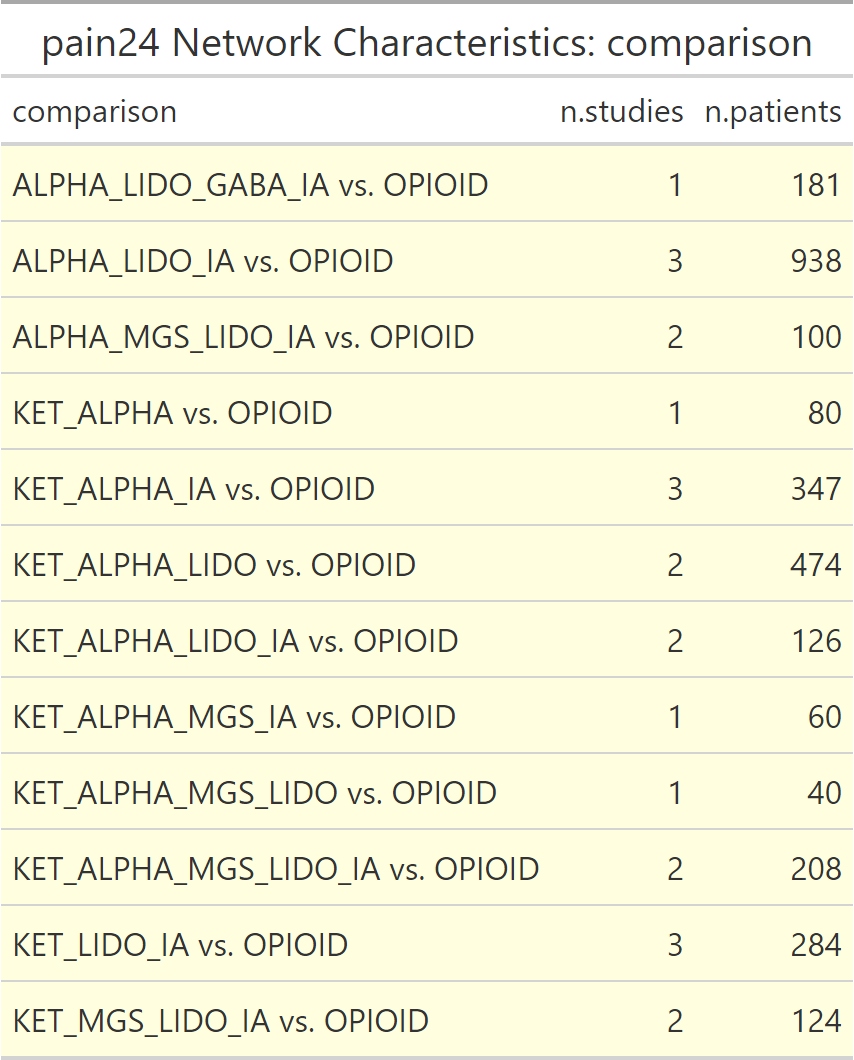


## Supplementary Figure 5: Pain intensity at 24 hours – Nodesplit analysis


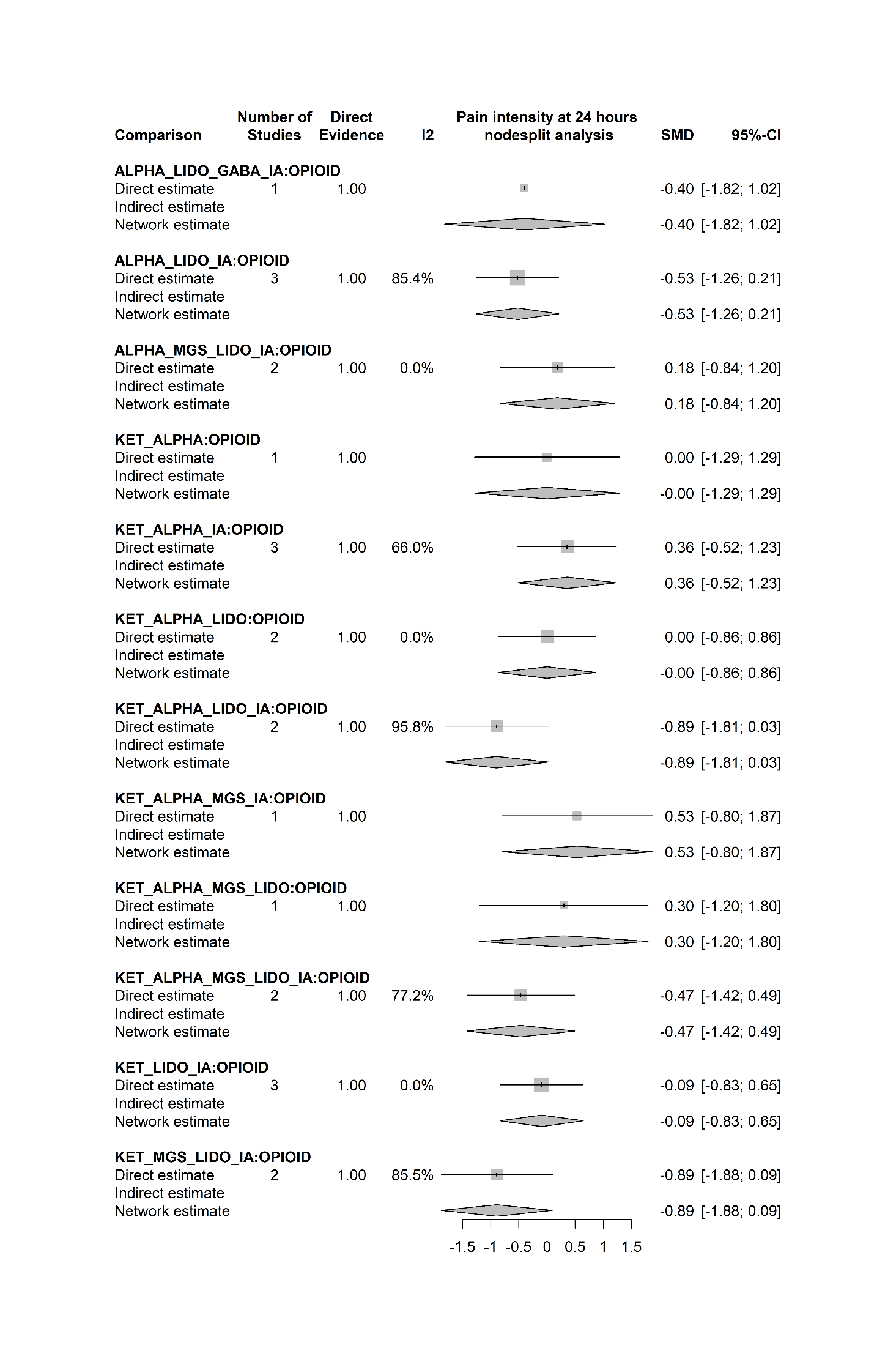


## Supplementary Figure 6: Pain intensity at 24 hours – Rankplot


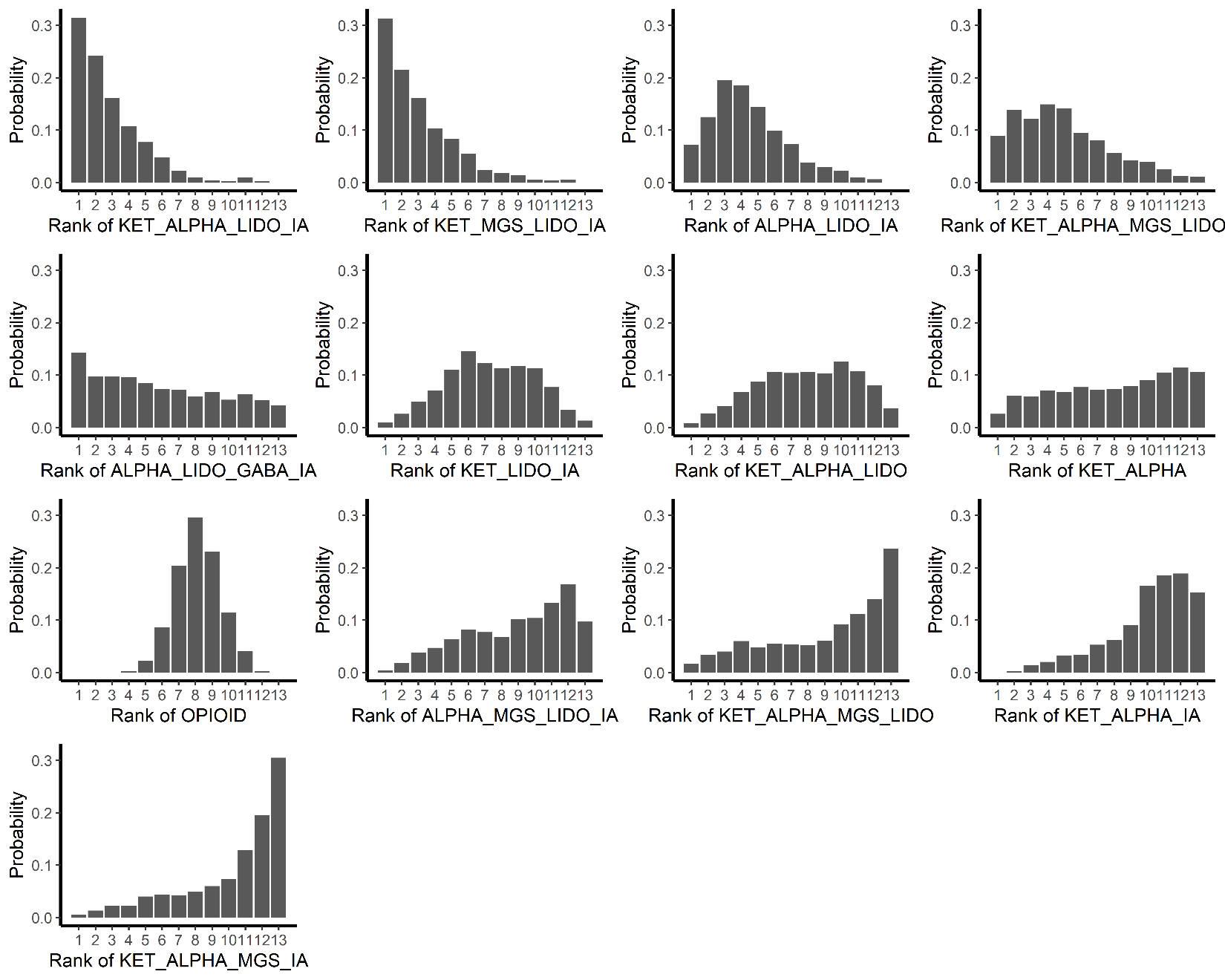


## Supplementary Figure 7: Pain intensity at 24 hours – Results of the individual studies


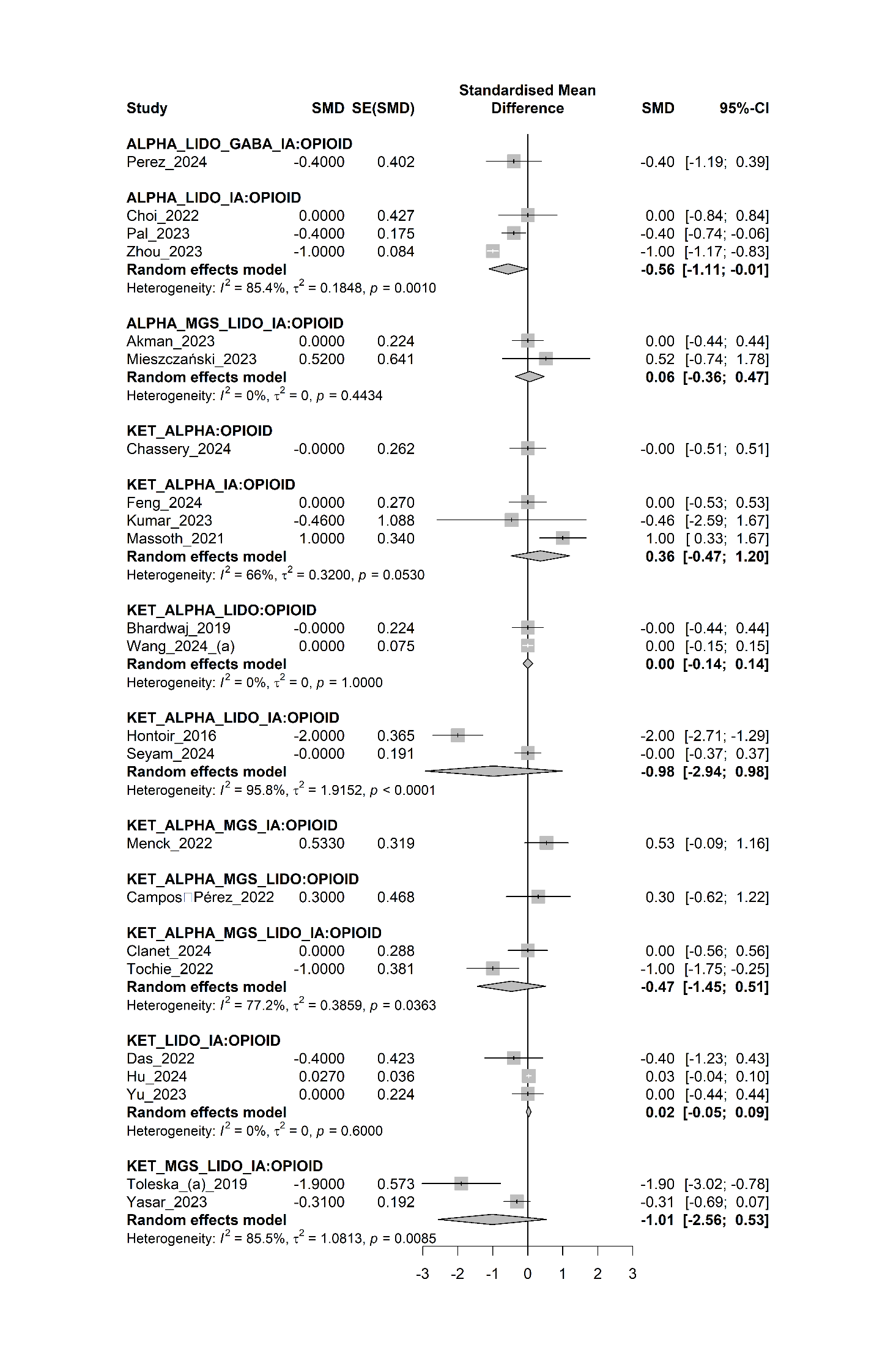


## Supplementary Figure 8: Pain intensity at 24 hours – Forest plot of the pairwise meta-analysis


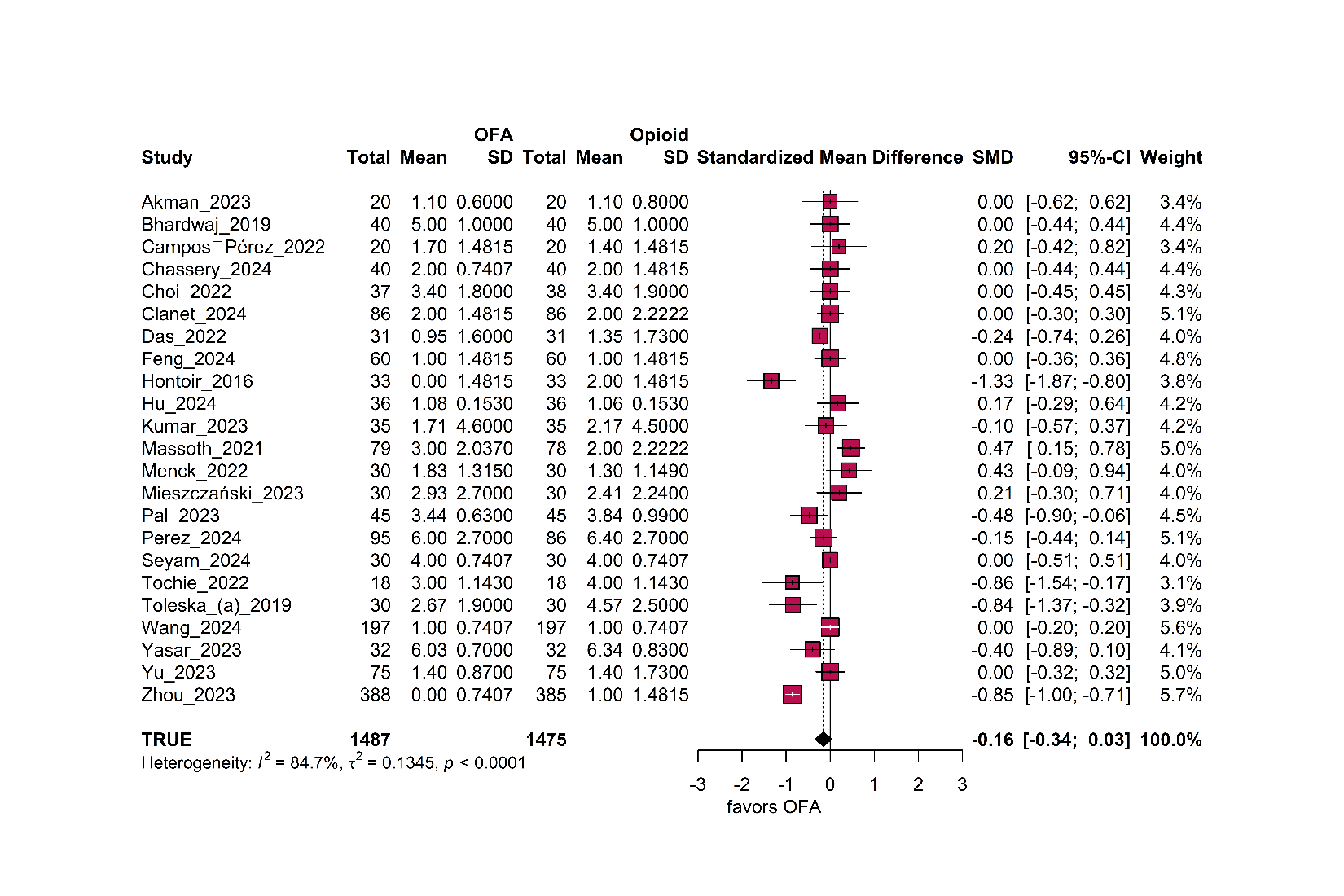


## Supplementary Figure 9: Pain intensity at 24 hours – Funnel plot of the pairwise meta-analysis


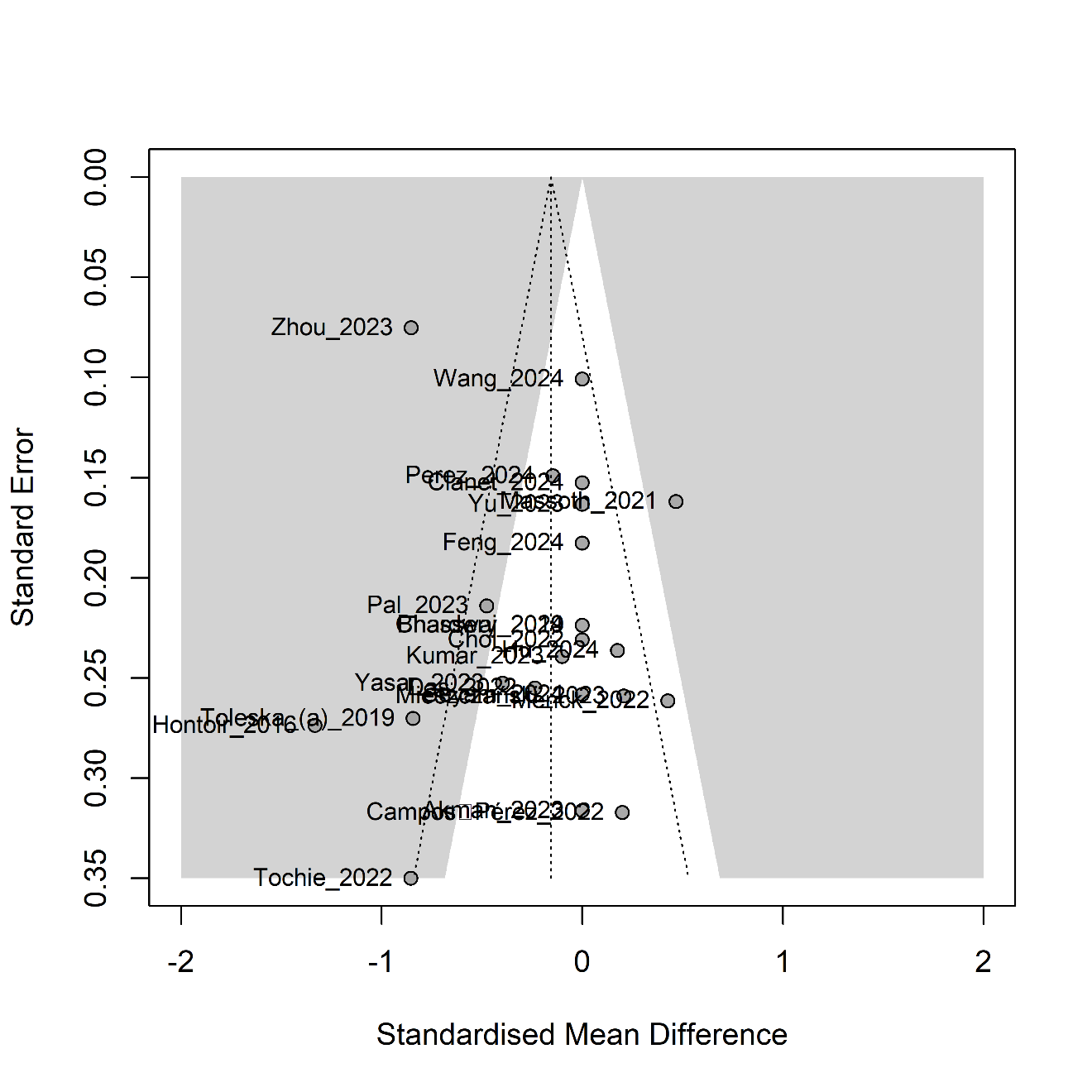


## Supplementary Figure 10: Pain intensity at 24 hours – Network plot of the ROB2 sub-analysis


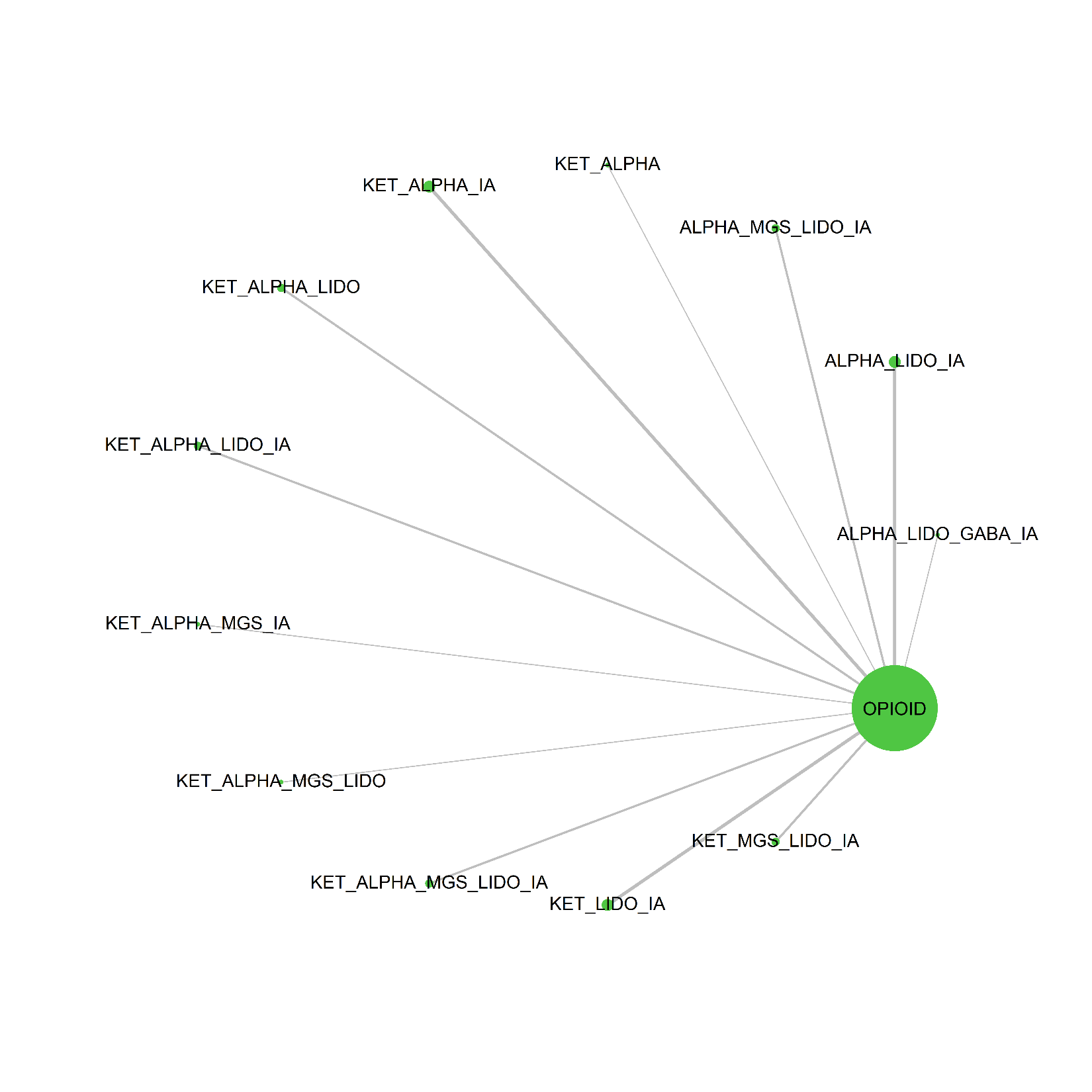


## Supplementary Figure 11: Pain intensity at 24 hours – Forest plot of the ROB2 sub-analysis


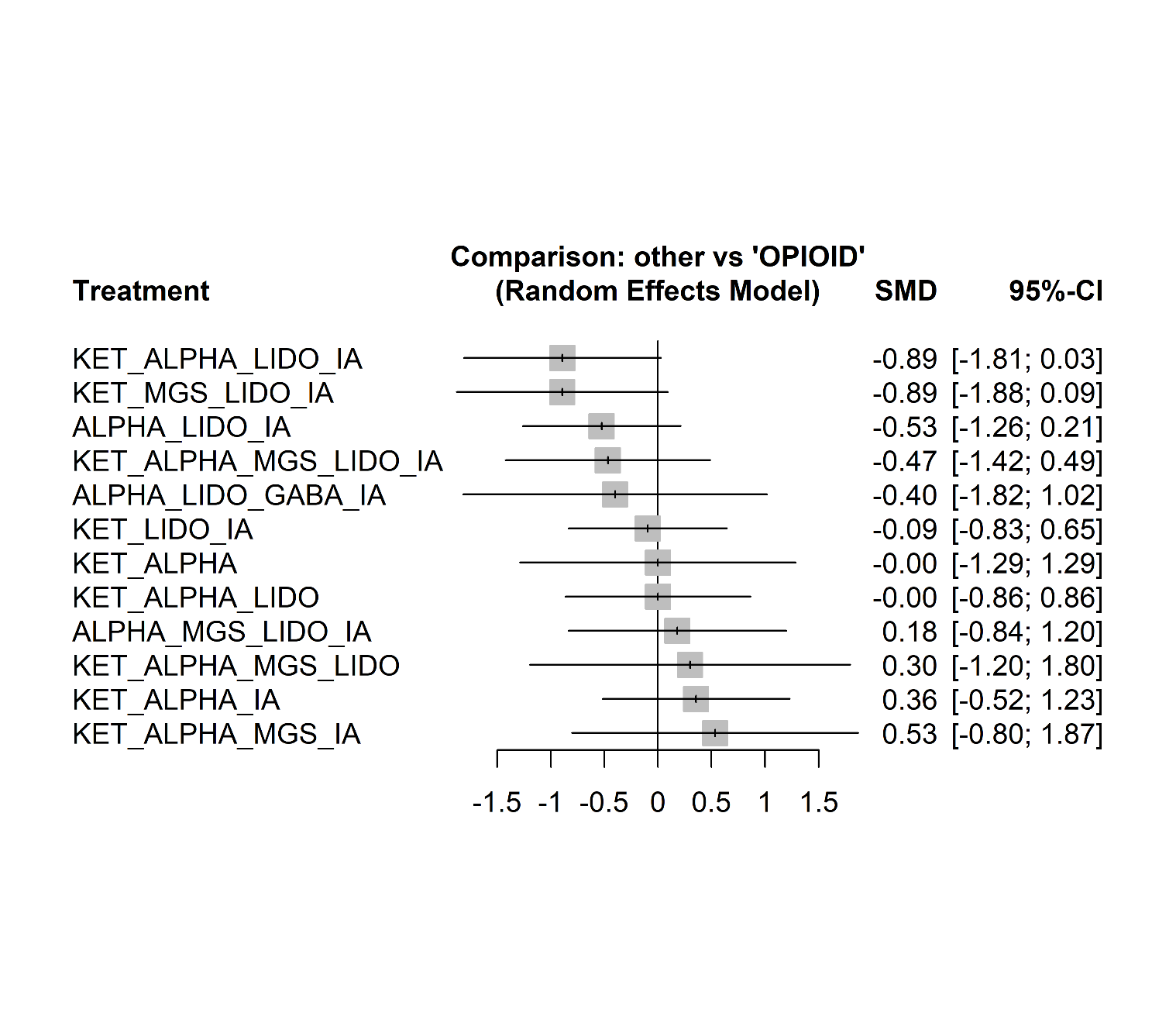


## Supplementary Figure 12: Pain intensity at 24 hours – Heatplot of the ROB2 sub-analysis


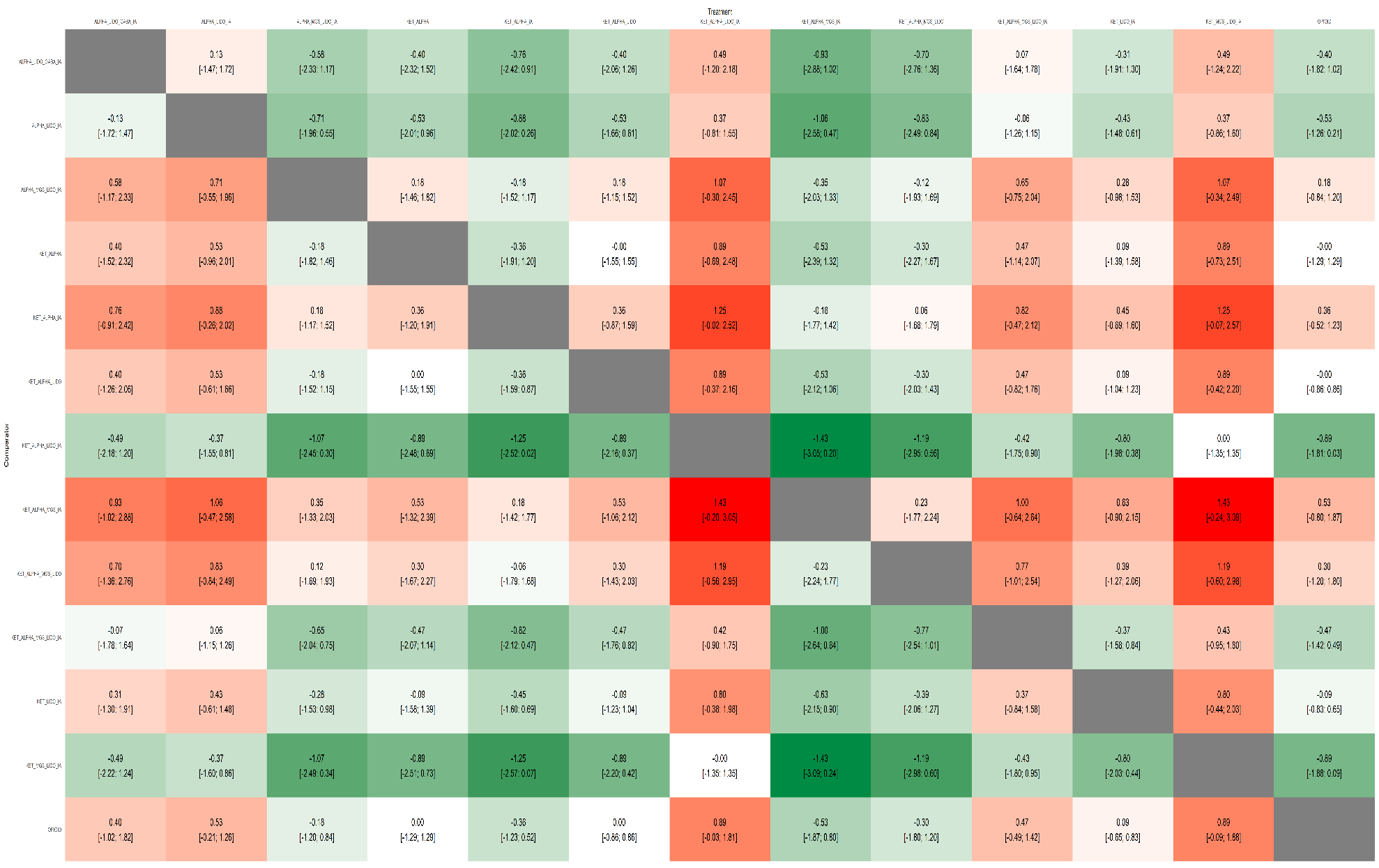


## Supplementary Figure 13: Pain intensity at 24 hours – Rankplot of the ROB2 sub-analysis


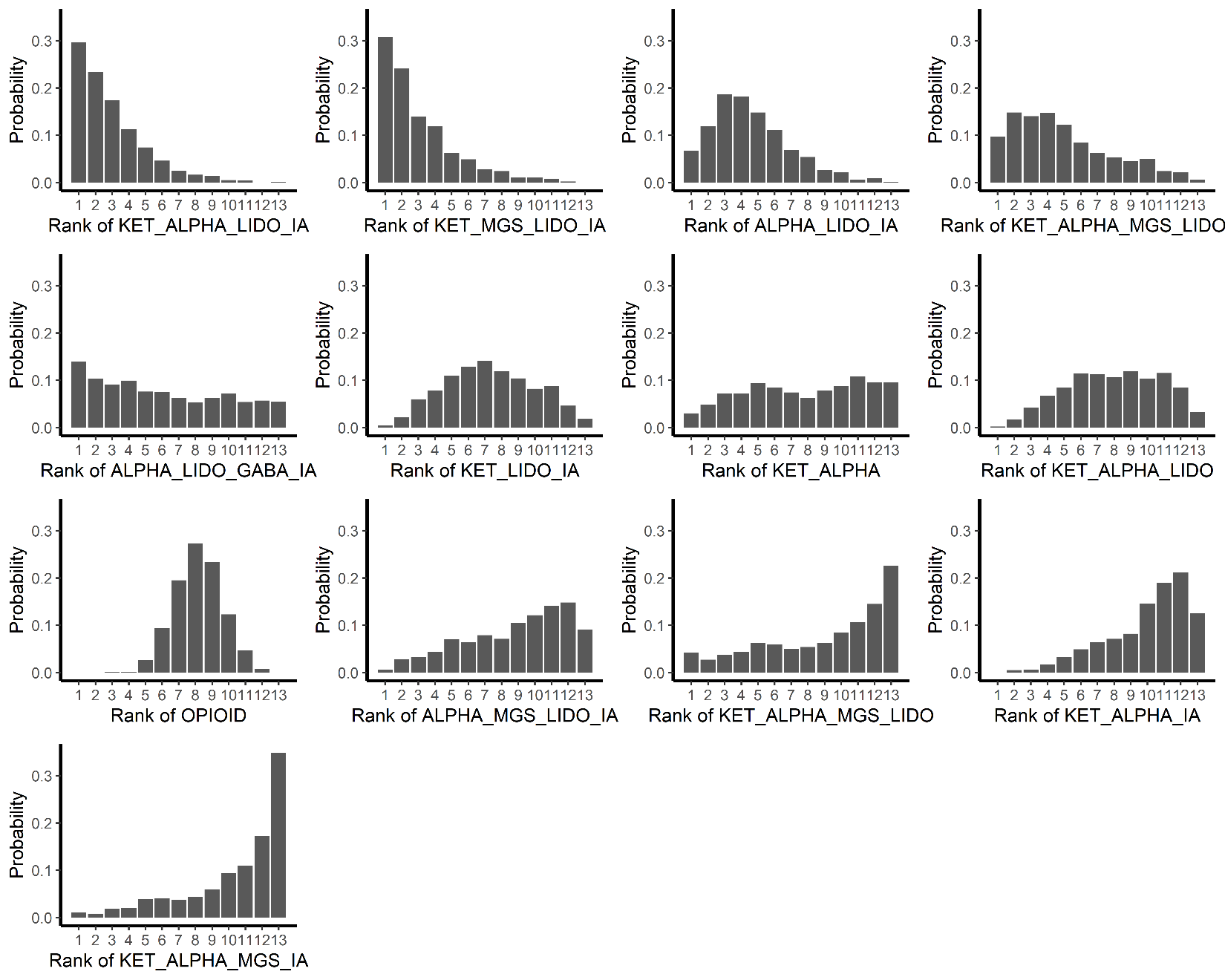


## Supplementary Figure 14: Pain intensity at 24 hours – CINeMA assessment


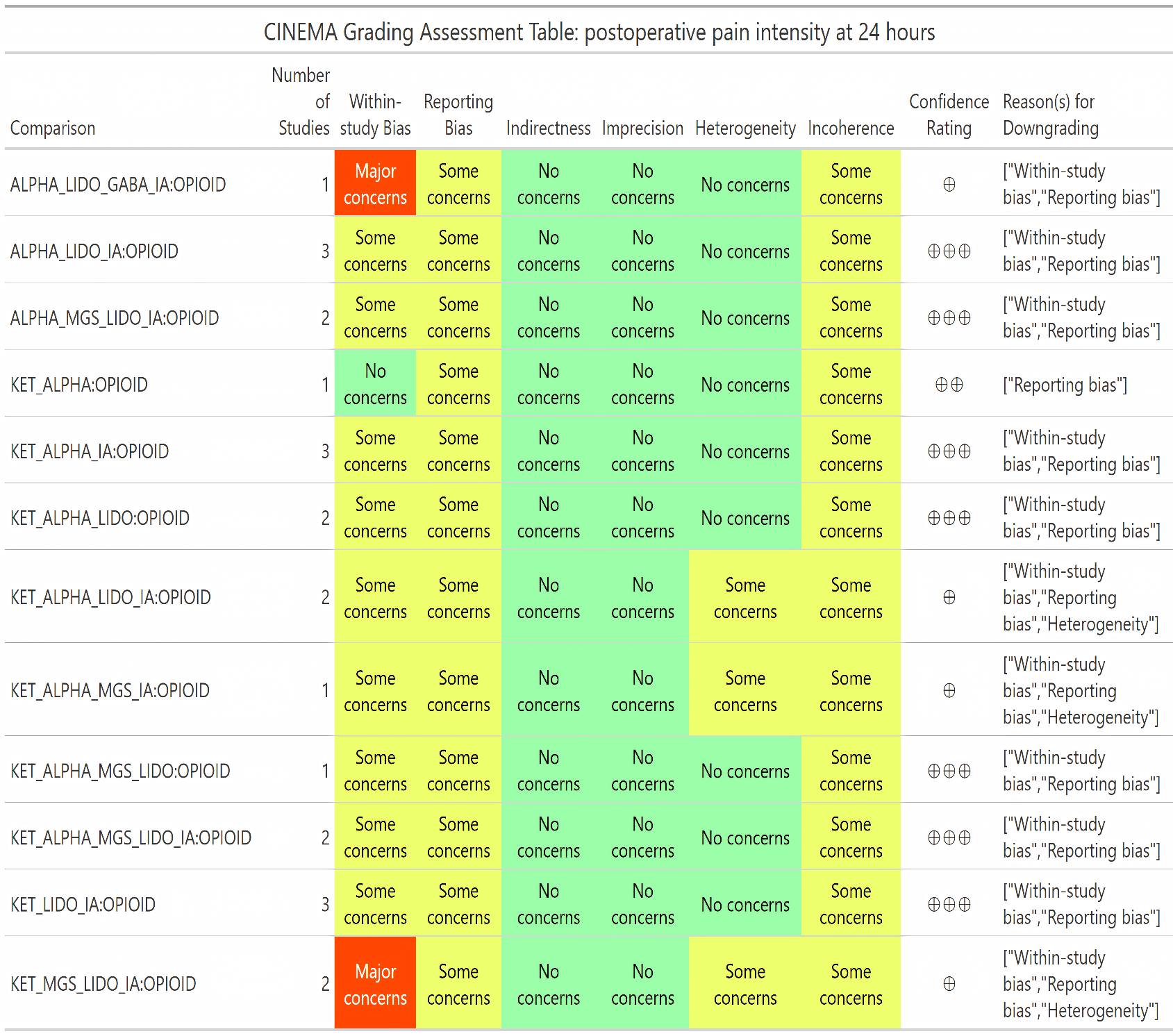


Legend: ⨁⨁⨁⨁ High confidence, ⨁⨁⨁ Moderate confidence, ⨁⨁ Low confidence, ⨁ Very low confidence

# Pain intensity at 0-2 hours: Supplementary Figures 15-24

## Supplementary Figure 15: Pain intensity at 0-2 hours – Network characteristics


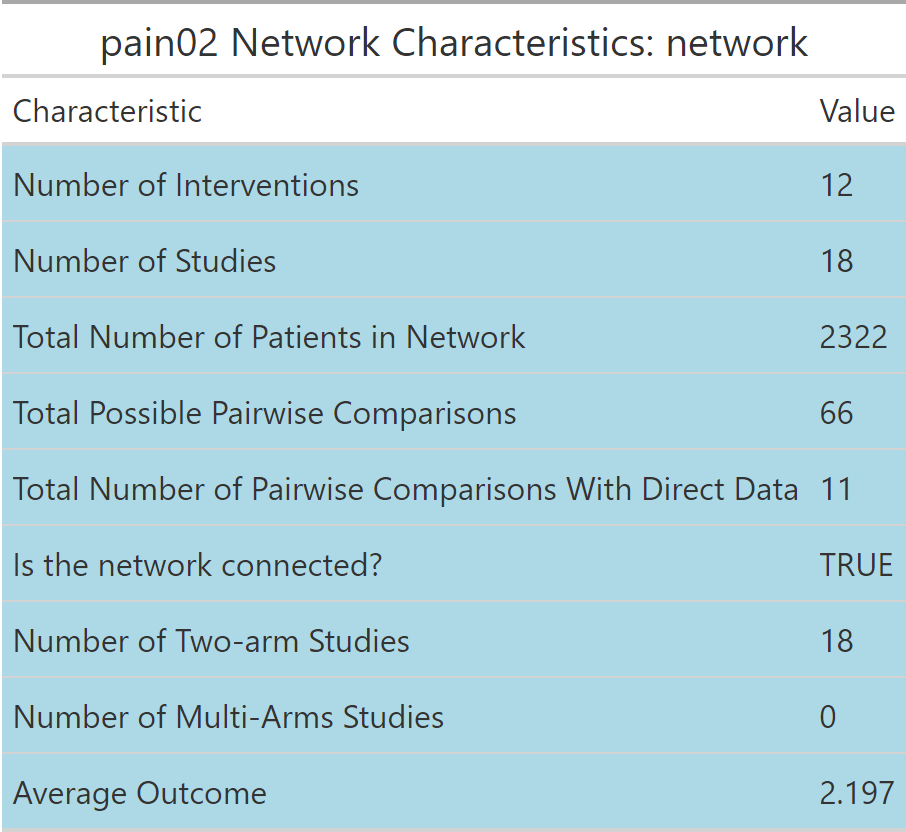


## Supplementary Figure 16: Pain intensity at 0-2 hours – Characteristics of the interventions


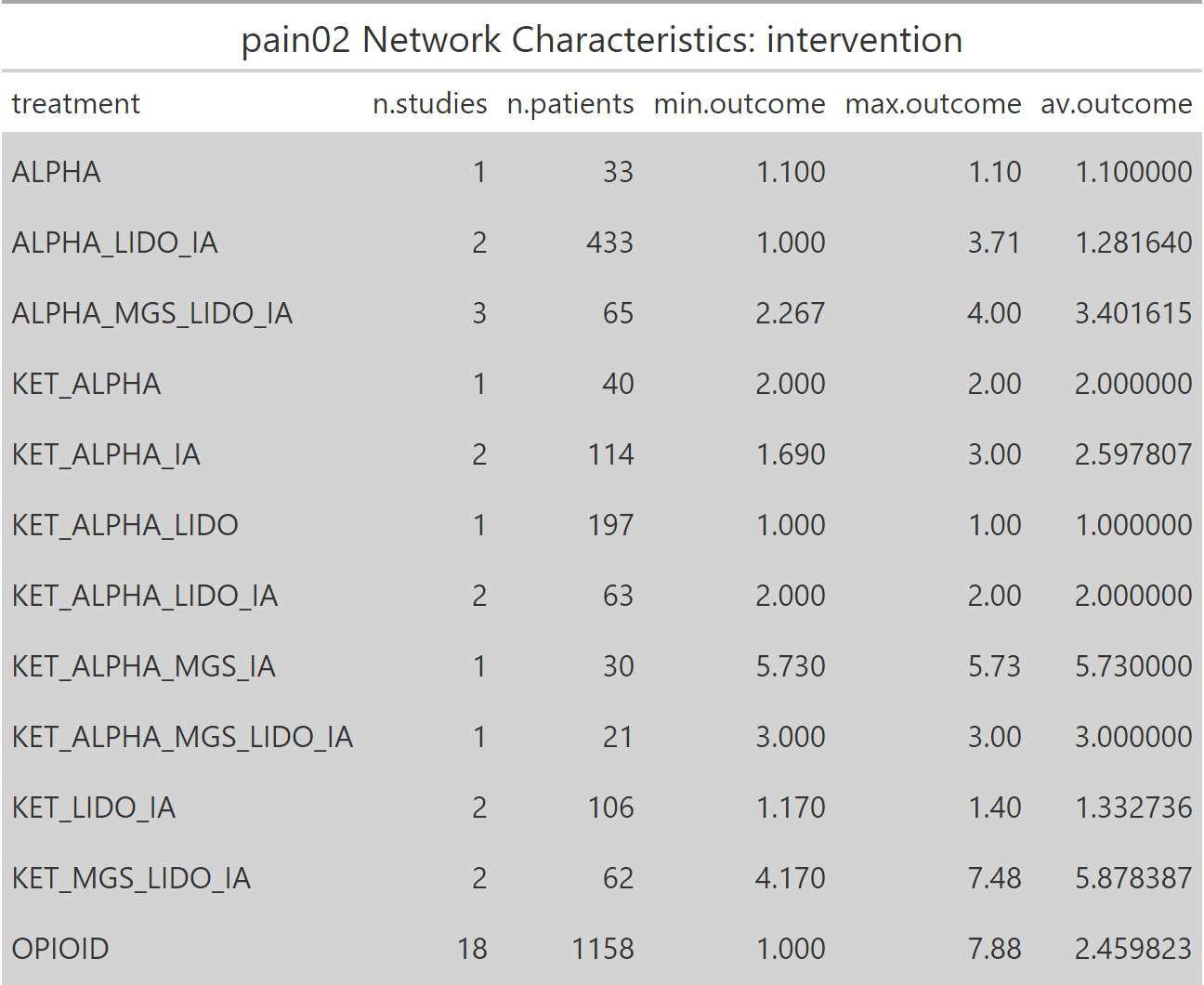


## Supplementary Figure 17: Pain intensity at 0-2 hours – Characteristics of the comparisons


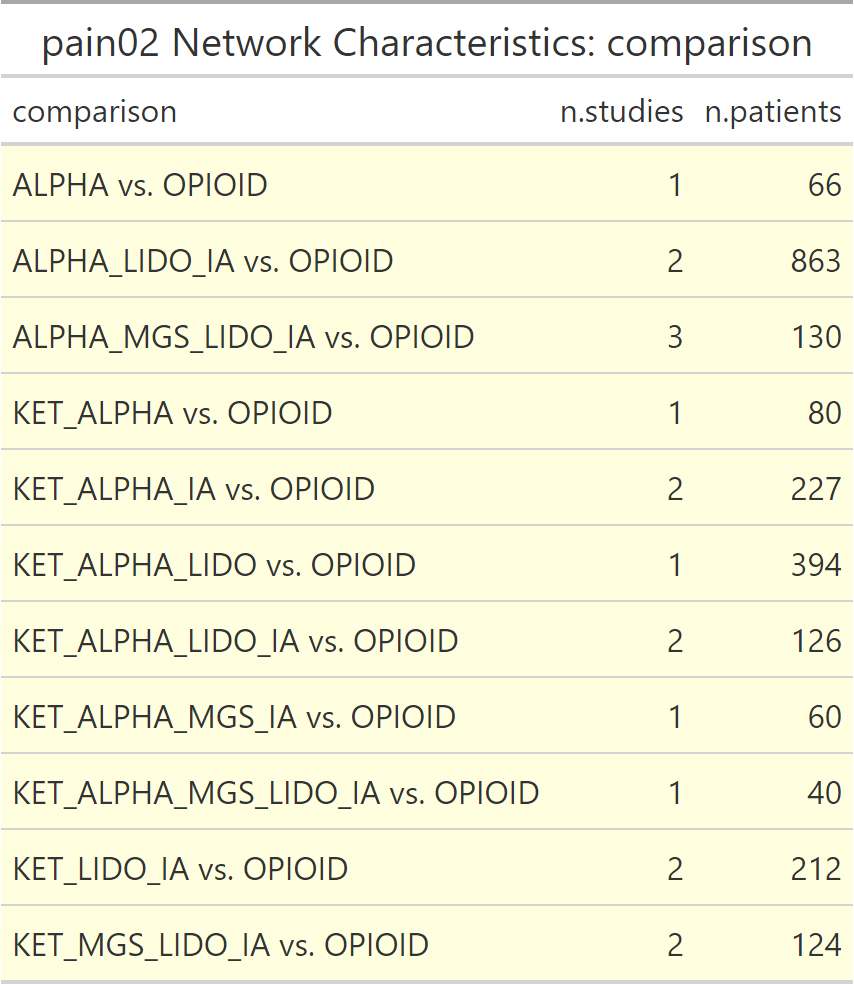


## Supplementary Figure 18: Pain intensity at 0-2 hours – Network plot


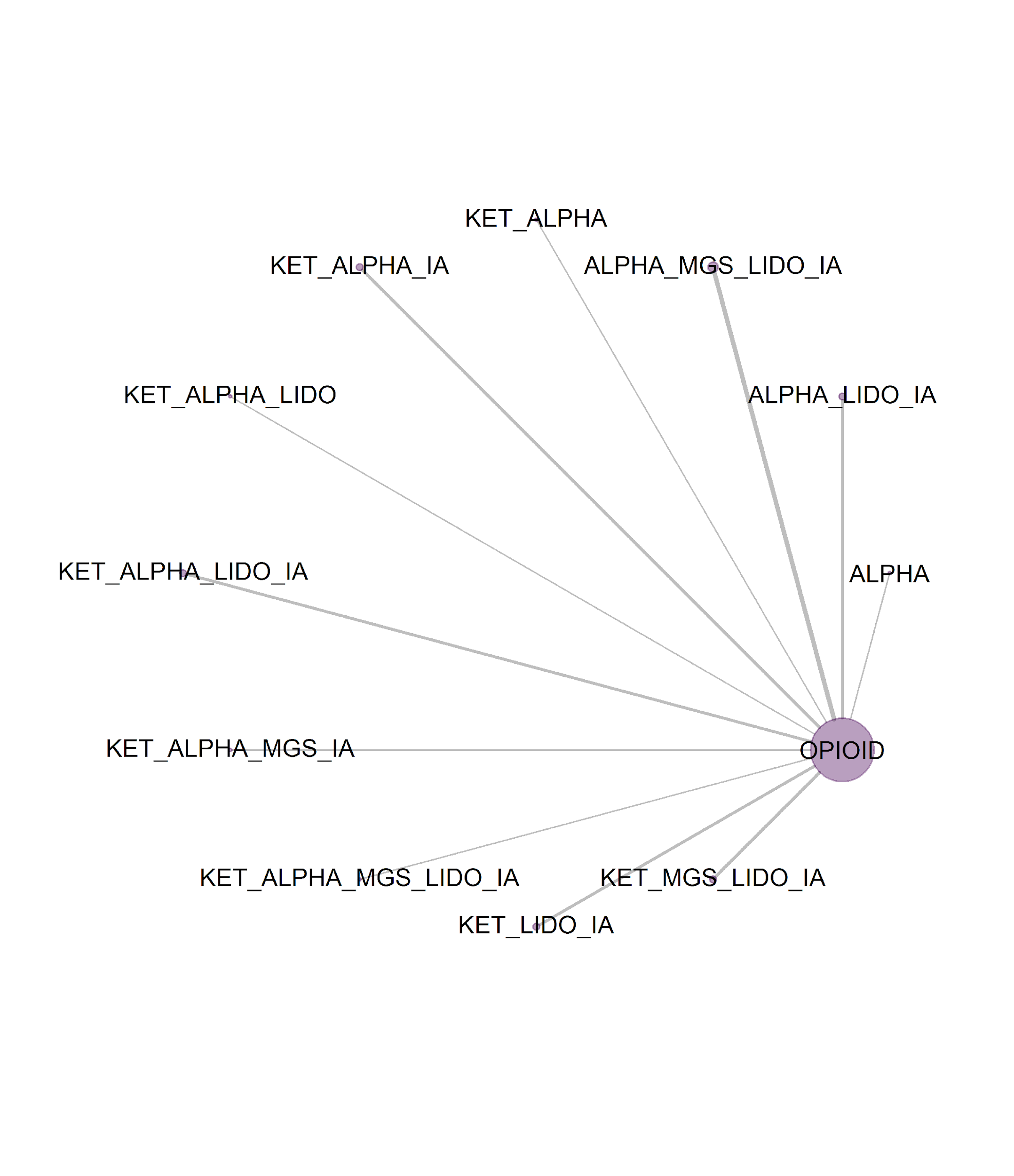


## Supplementary Figure 19: Pain intensity at 0-2 hours – Heatplot


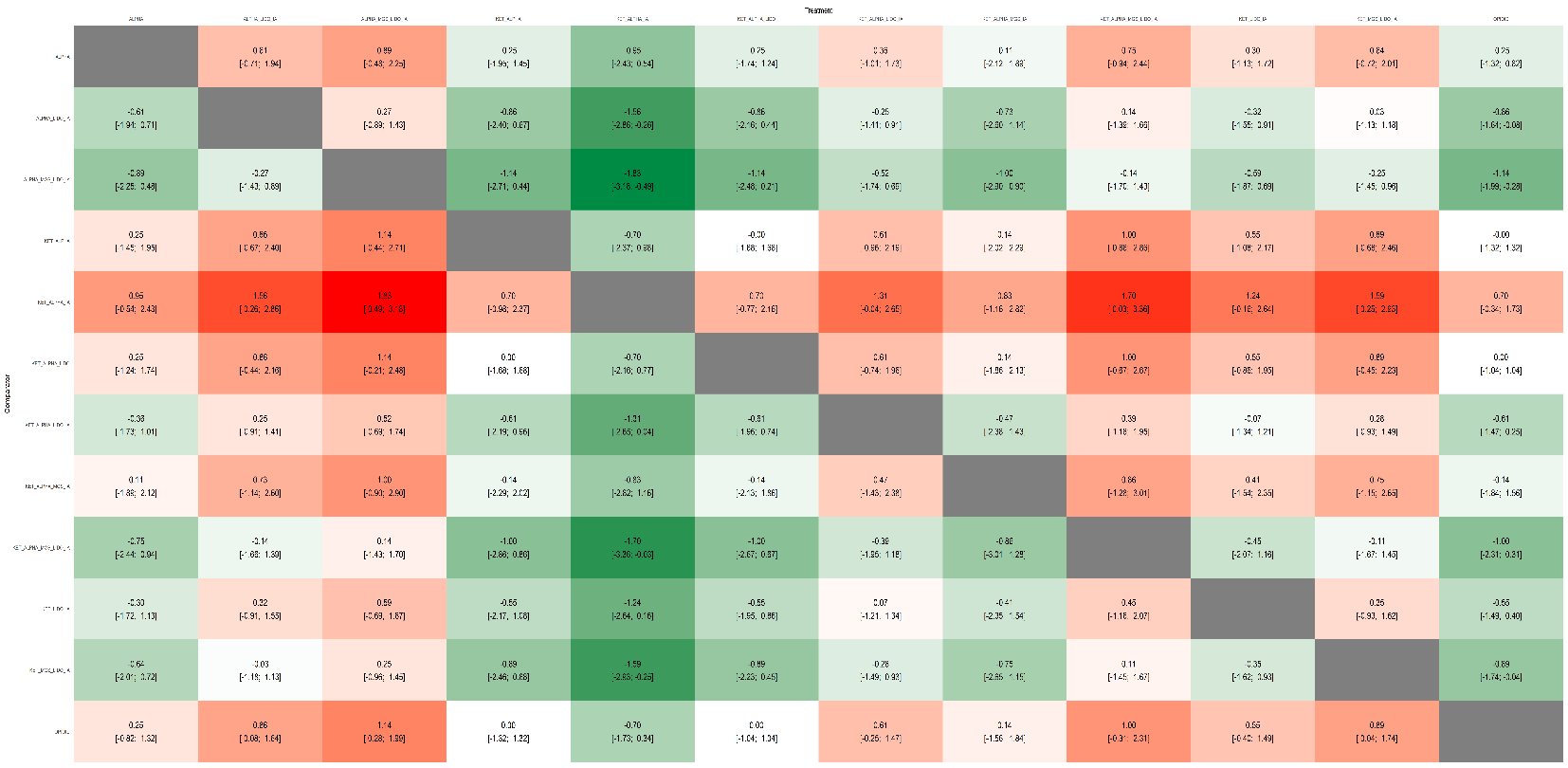


## Supplementary Figure 20: Pain intensity at 0-2 hours – Rankplot


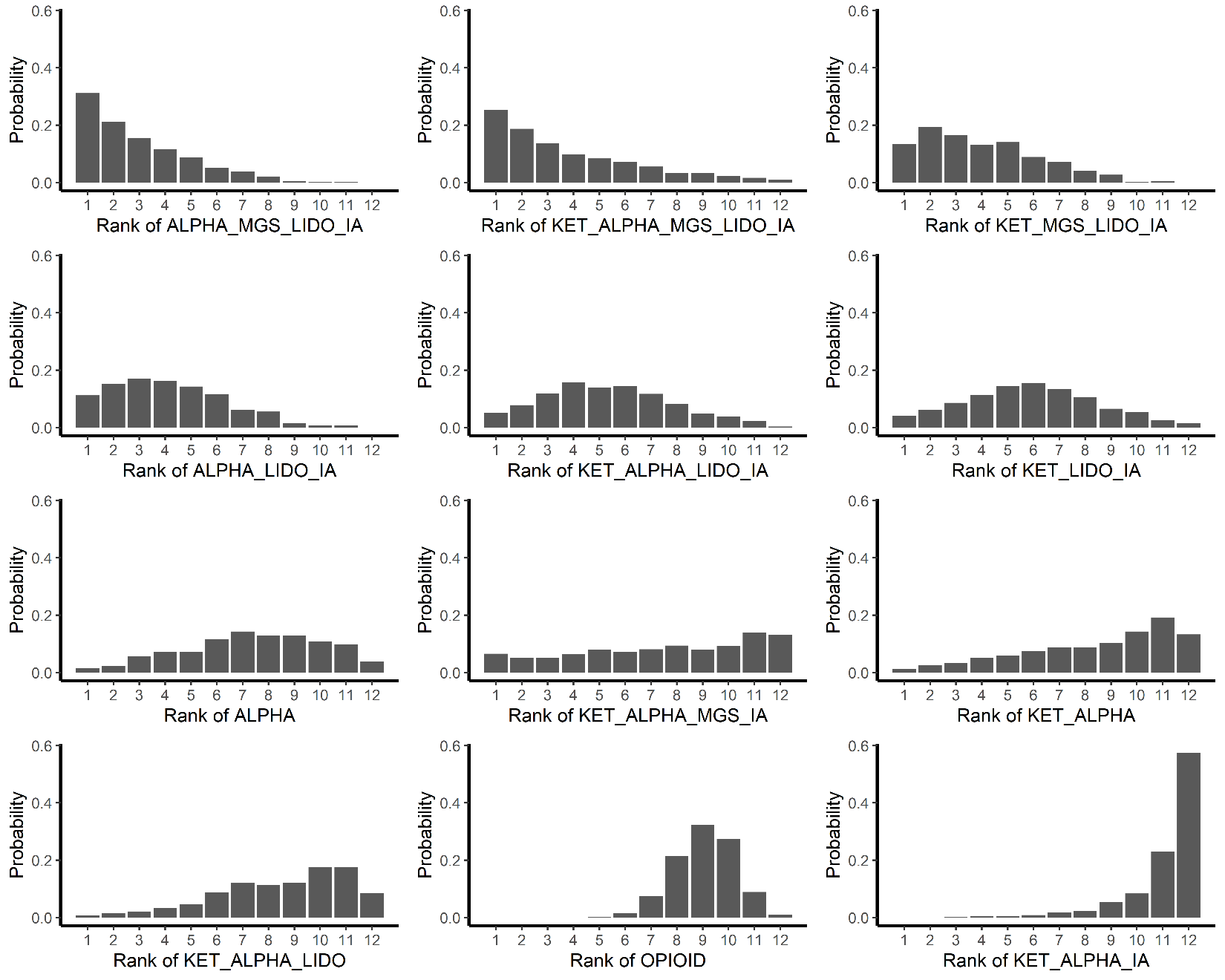


## Supplementary Figure 21: Pain intensity at 0-2 hours – Nodesplit analysis


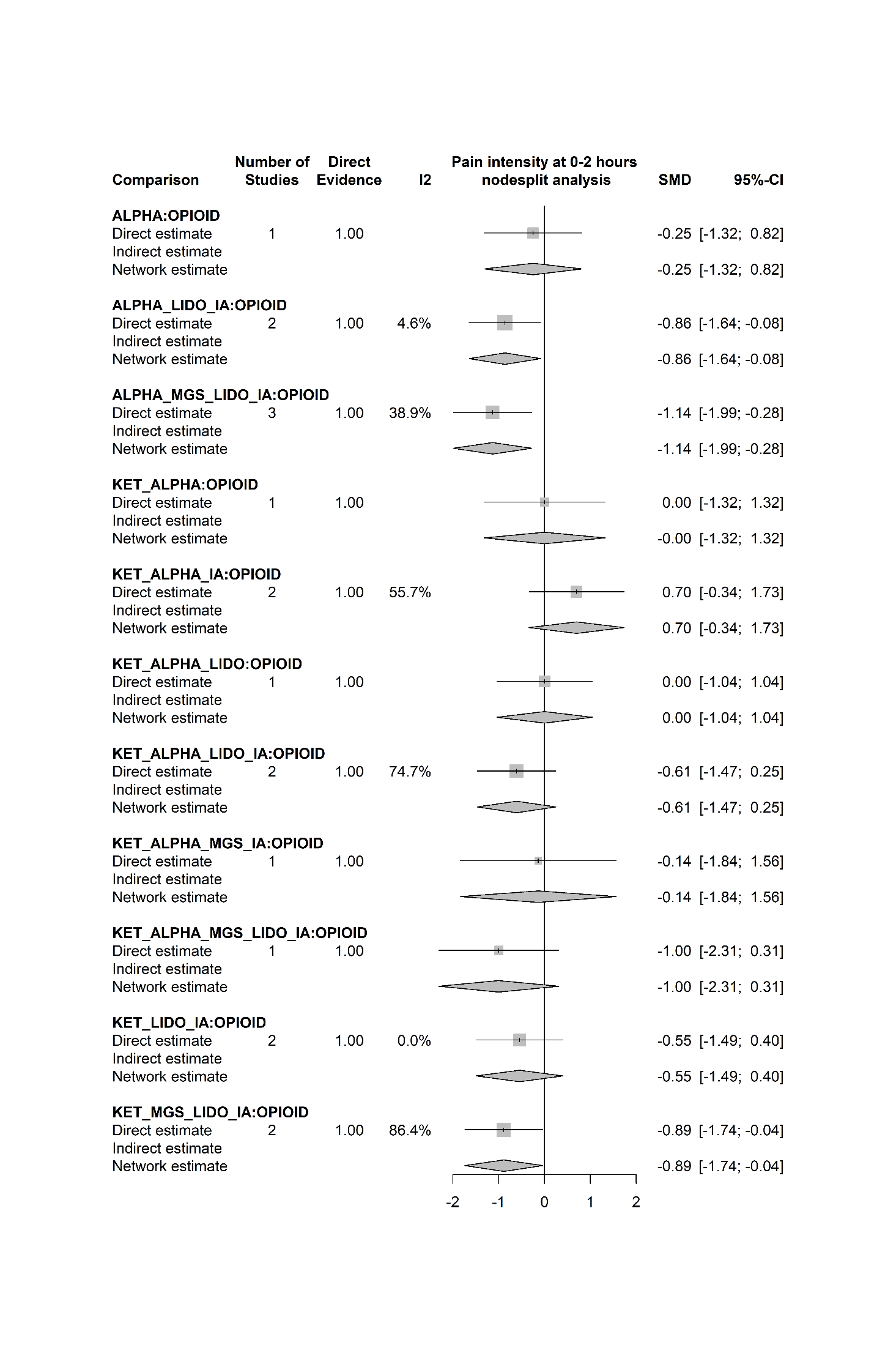


## Supplementary Figure 21: Pain intensity at 0-2 hours – Results of the individual studies


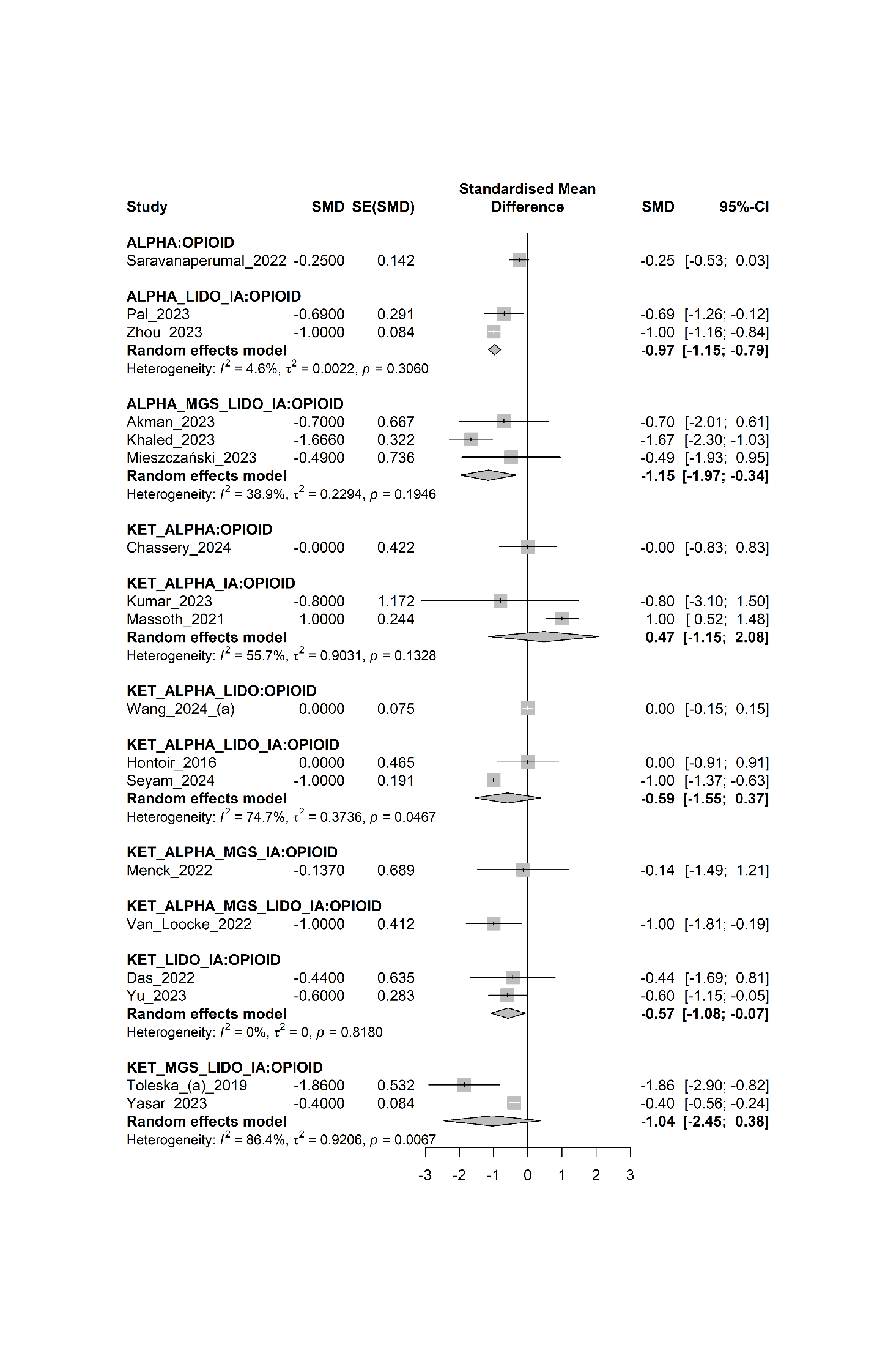


## Supplementary Figure 23: Pain intensity at 24 hours – Forest plot of the pairwise meta-analysis


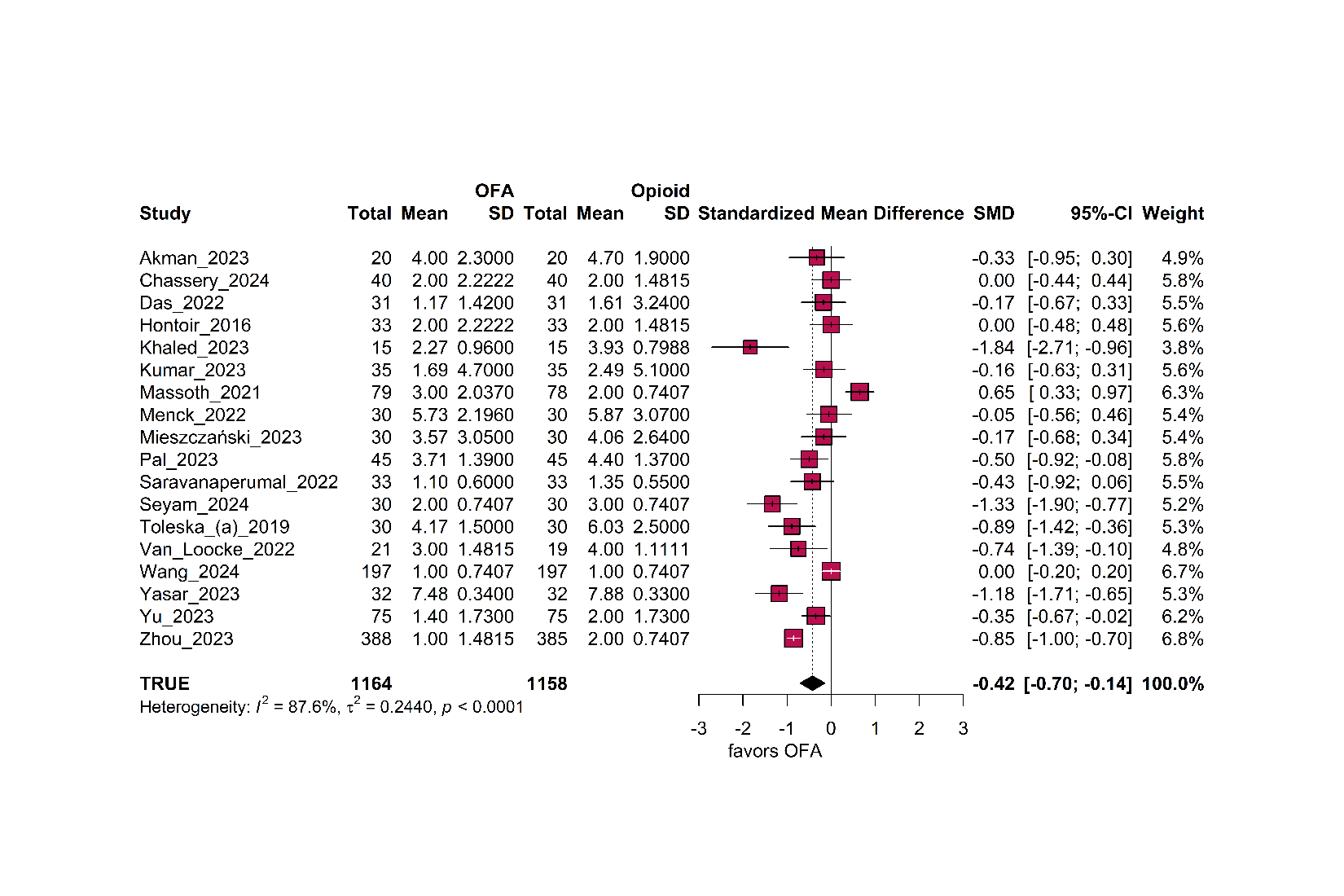


Supplementary Figure 24: Pain intensity at 24 hours – Funnel plot of the pairwise meta-analysis


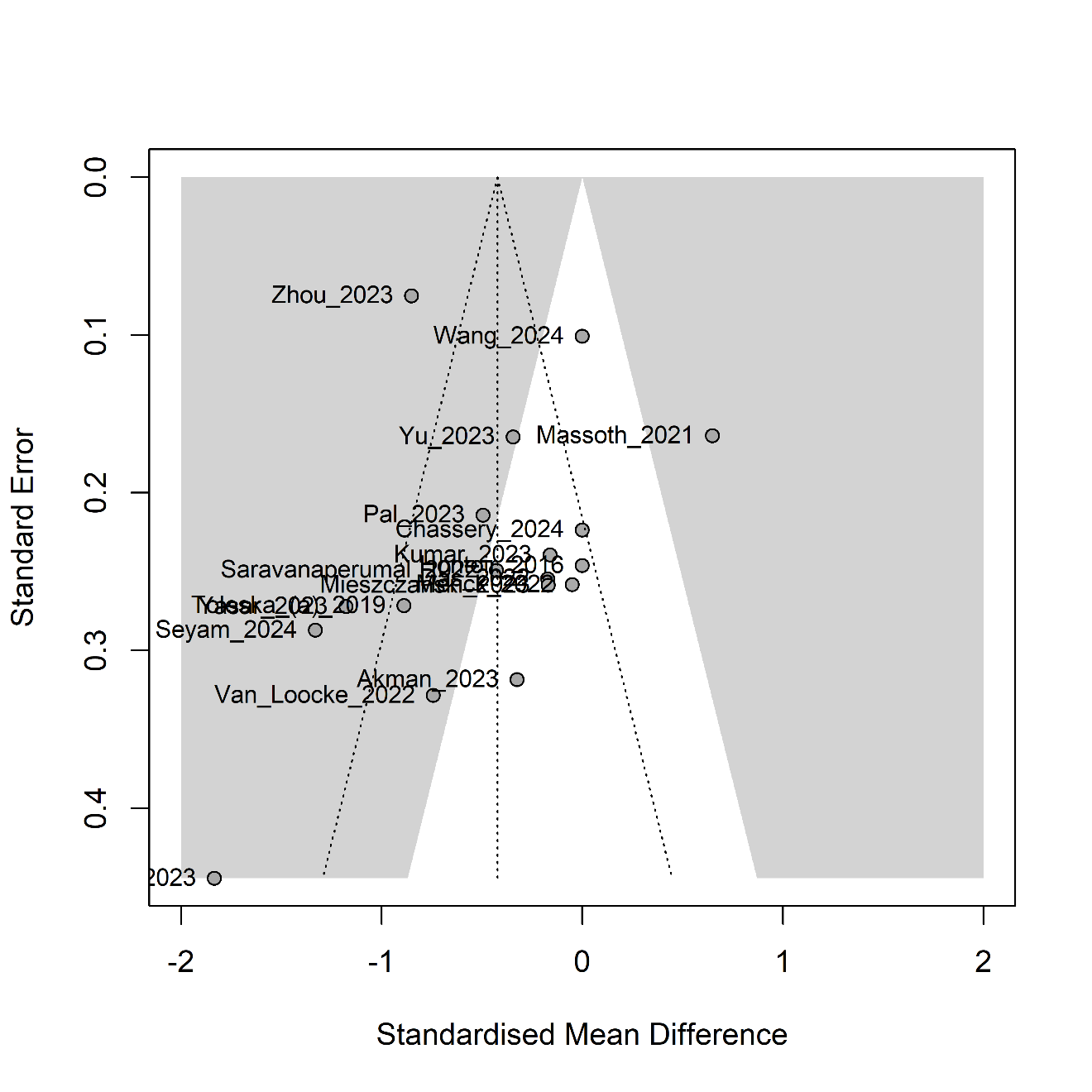


# Postoperative opioid consumption as oral morphine equivalents in milligrams: Supplementary Figures 25-34

## Supplementary Figure 25: Postoperative opioid consumption – Network characteristics


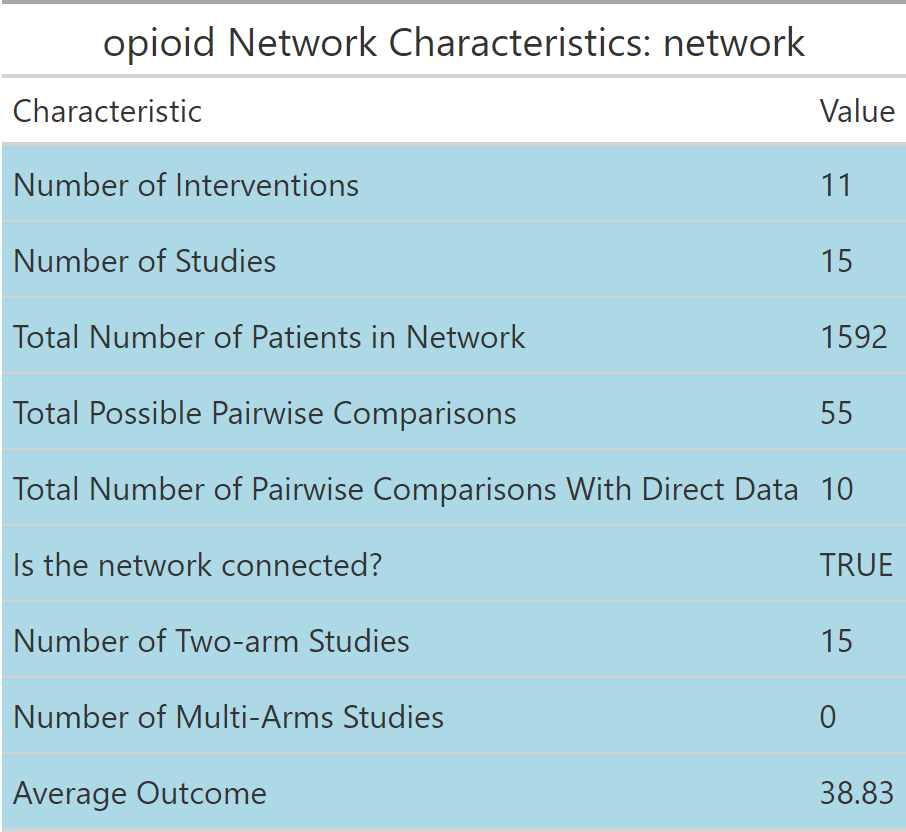


## Supplementary Figure 26: Postoperative opioid consumption – Characteristics of the interventions


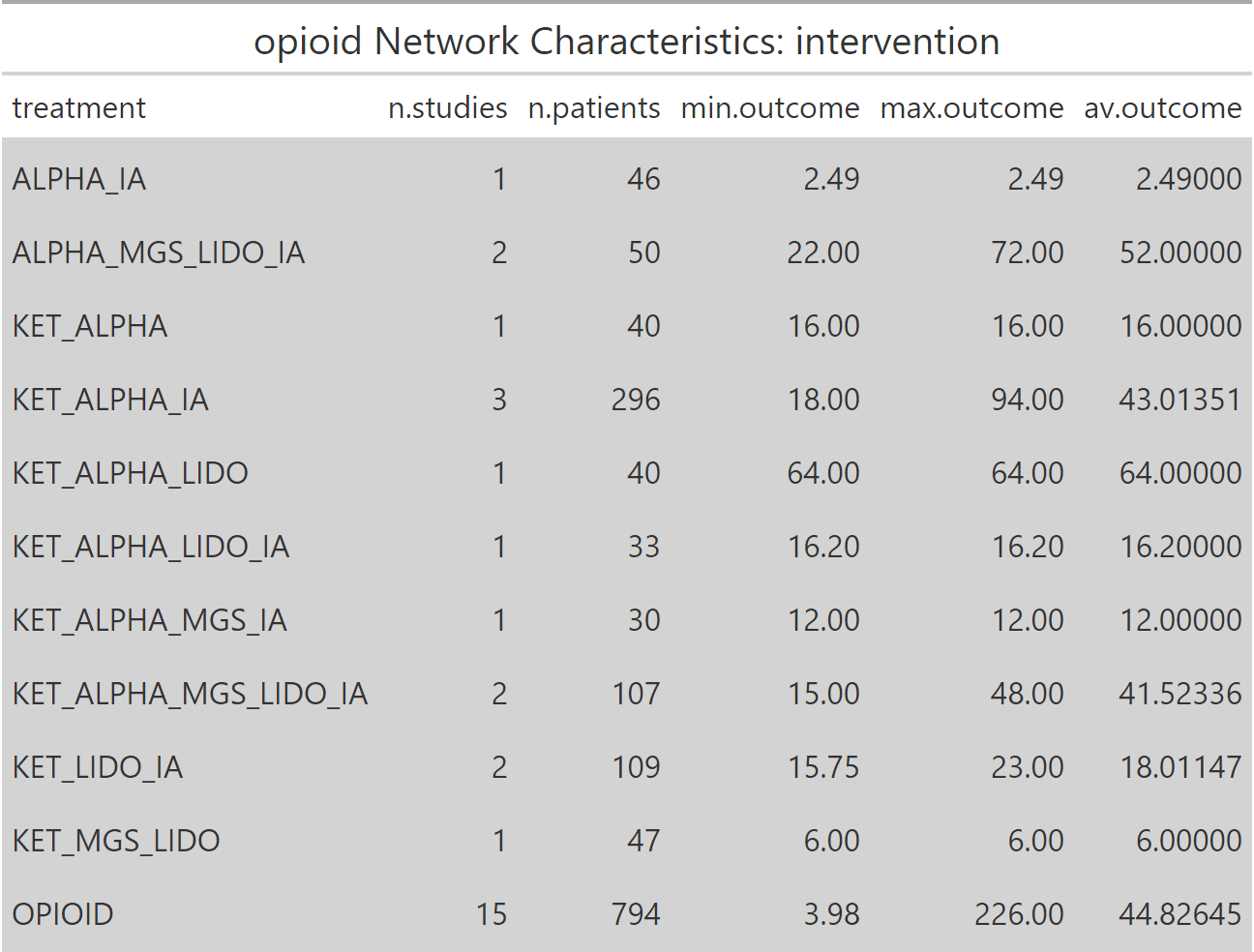


## Supplementary Figure 27: Postoperative opioid consumption – Characteristics of the comparisons


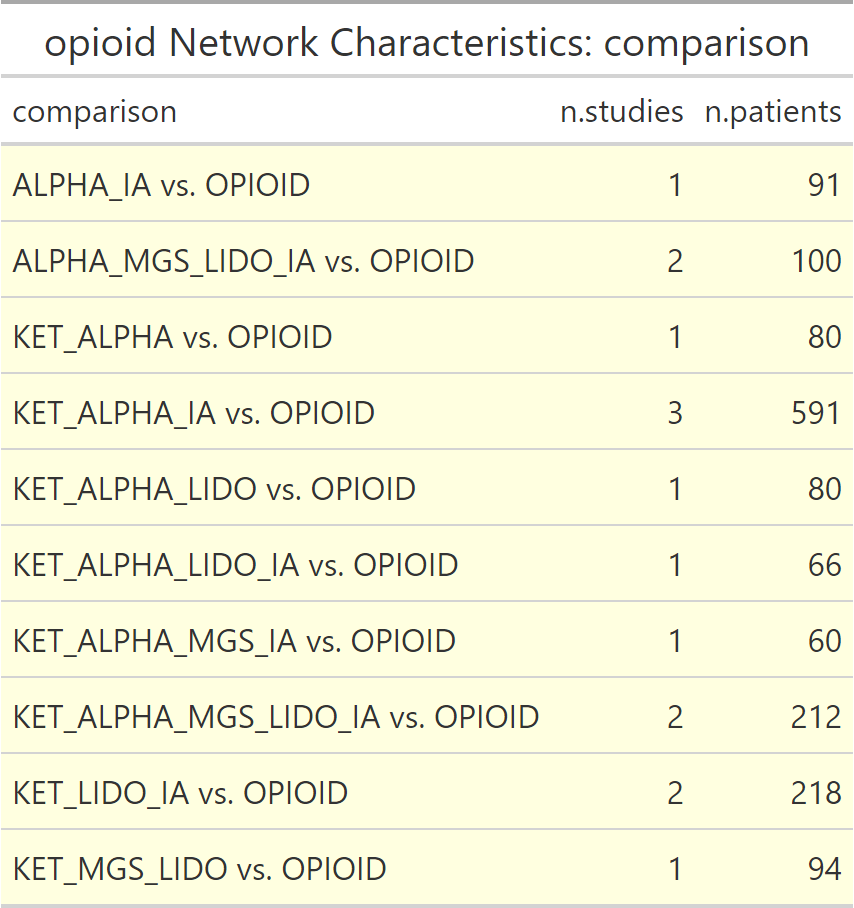


## Supplementary Figure 28: Postoperative opioid consumption – Network plot


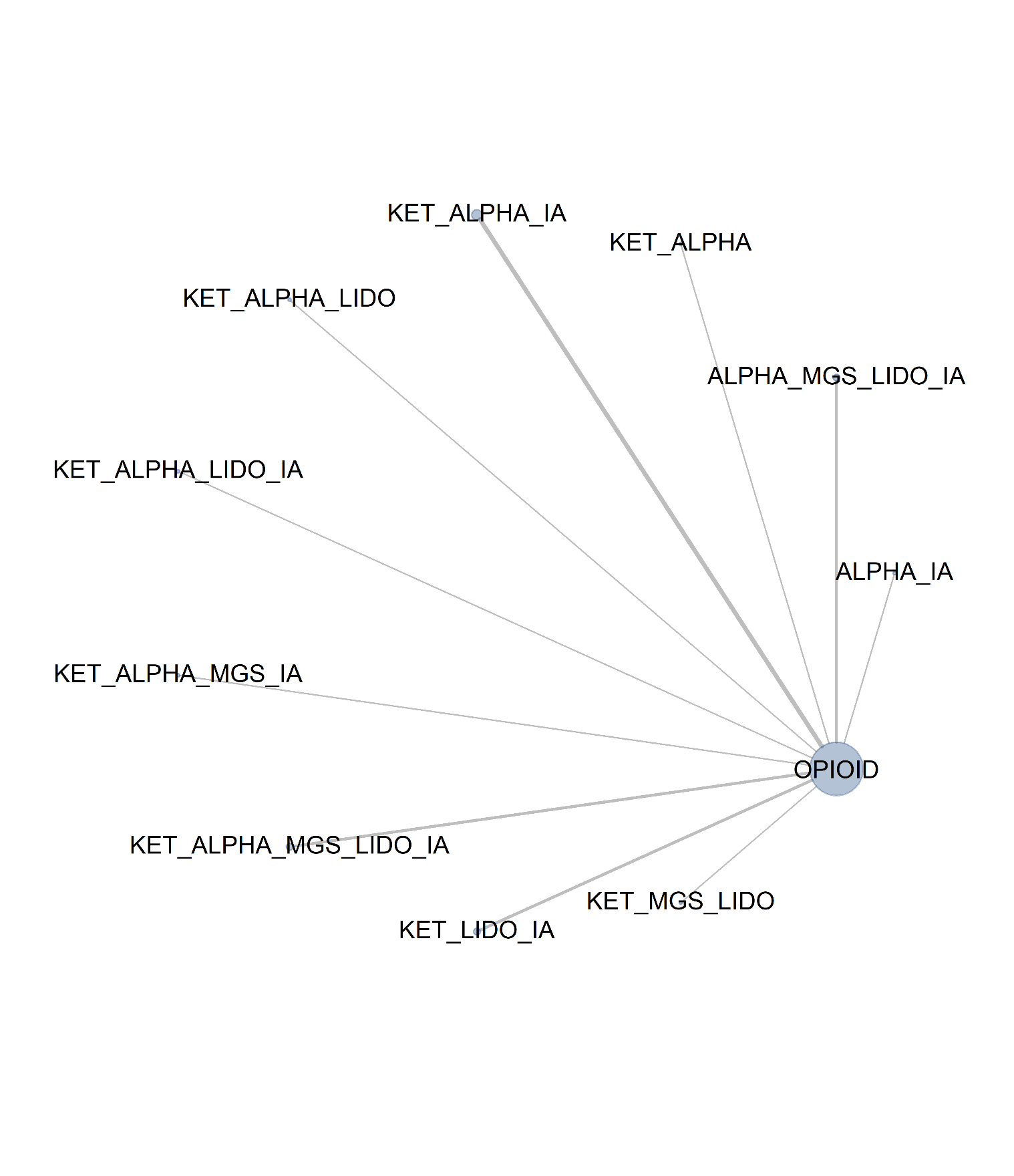


## Supplementary Figure 29: Postoperative opioid consumption – Heatplot


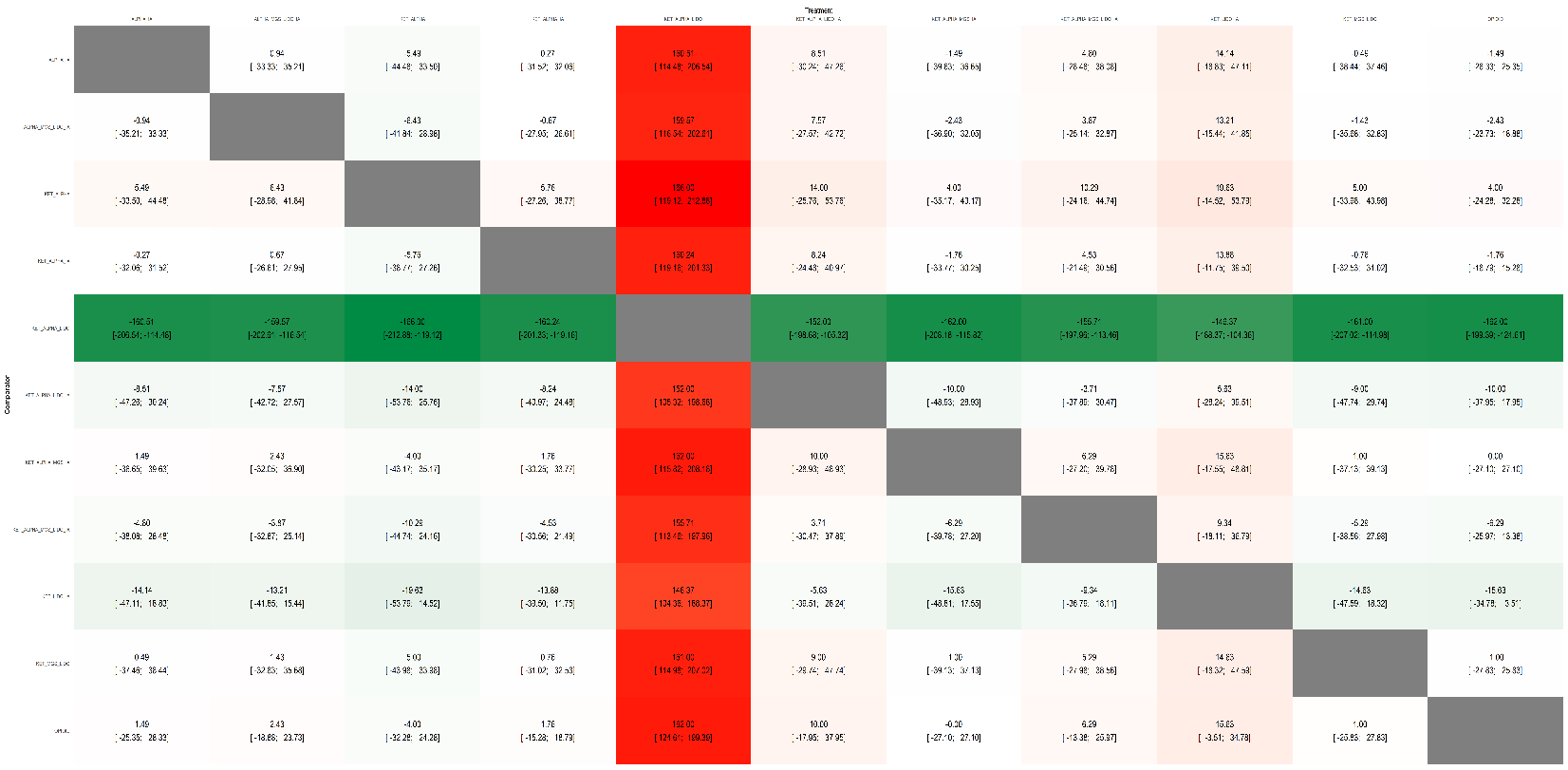


## Supplementary Figure 30: Postoperative opioid consumption – Rankplot


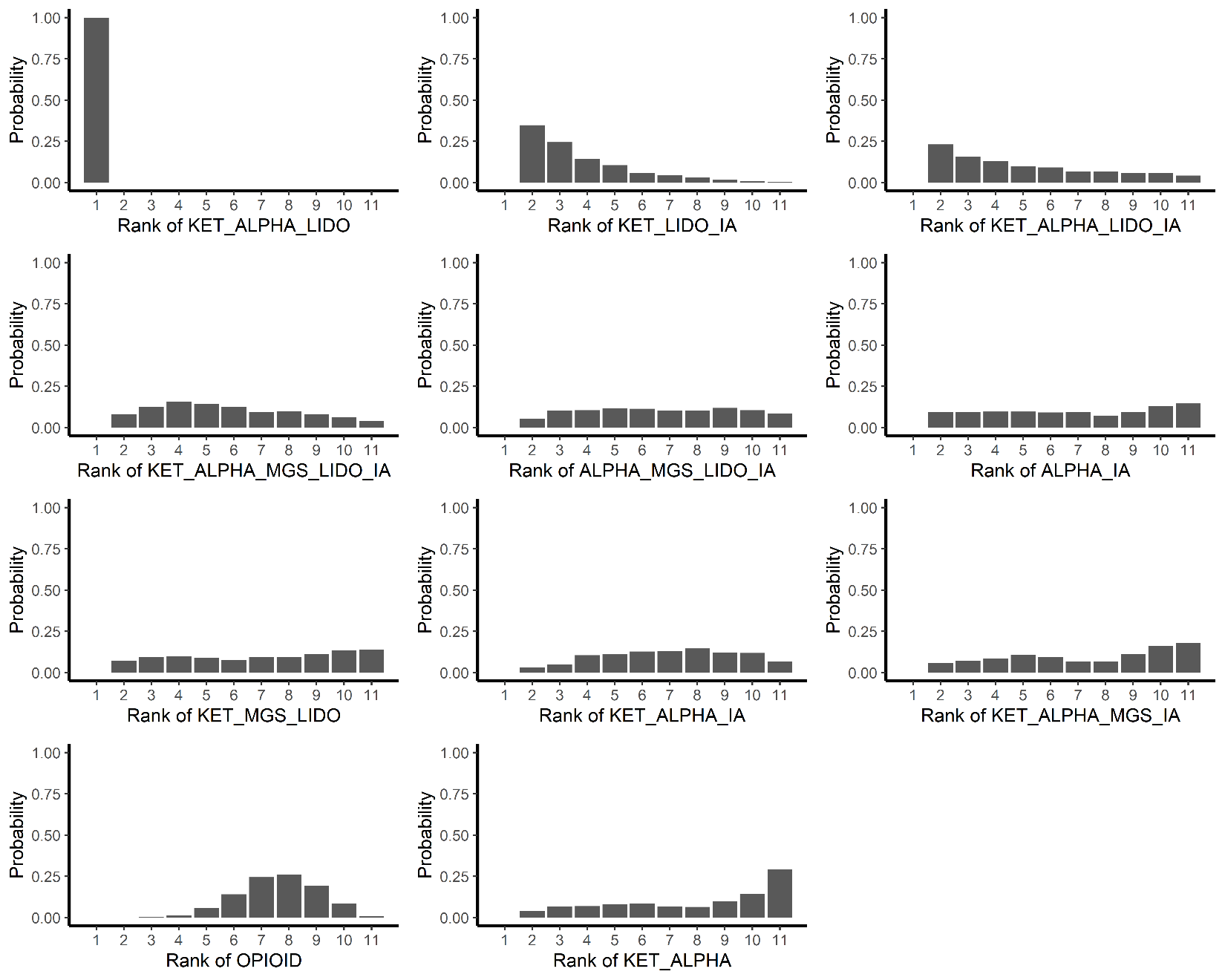


## Supplementary Figure 31: Postoperative opioid consumption – Nodesplit analysis


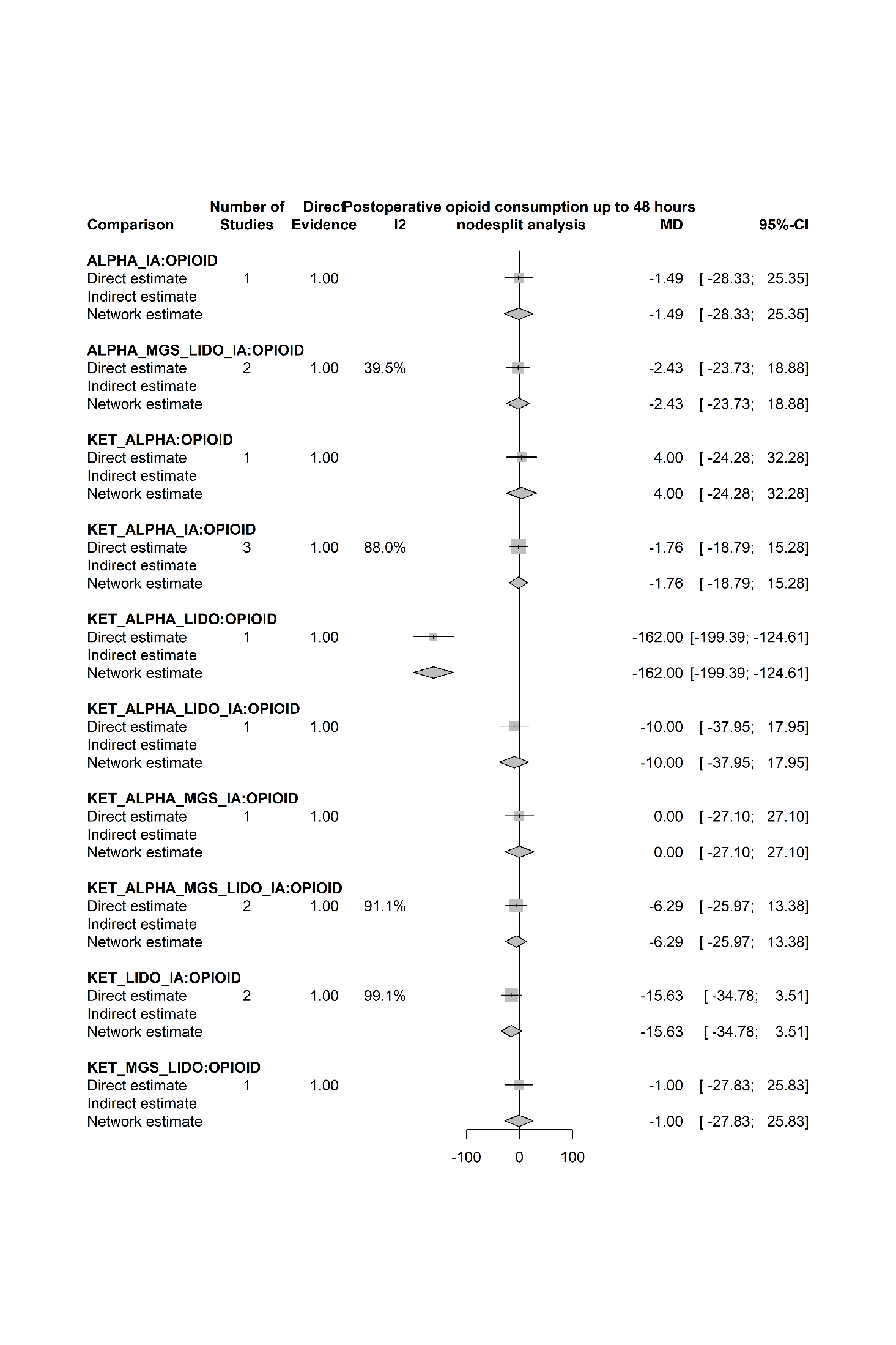


## Supplementary Figure 32: Postoperative opioid consumption – Result of the individual studies


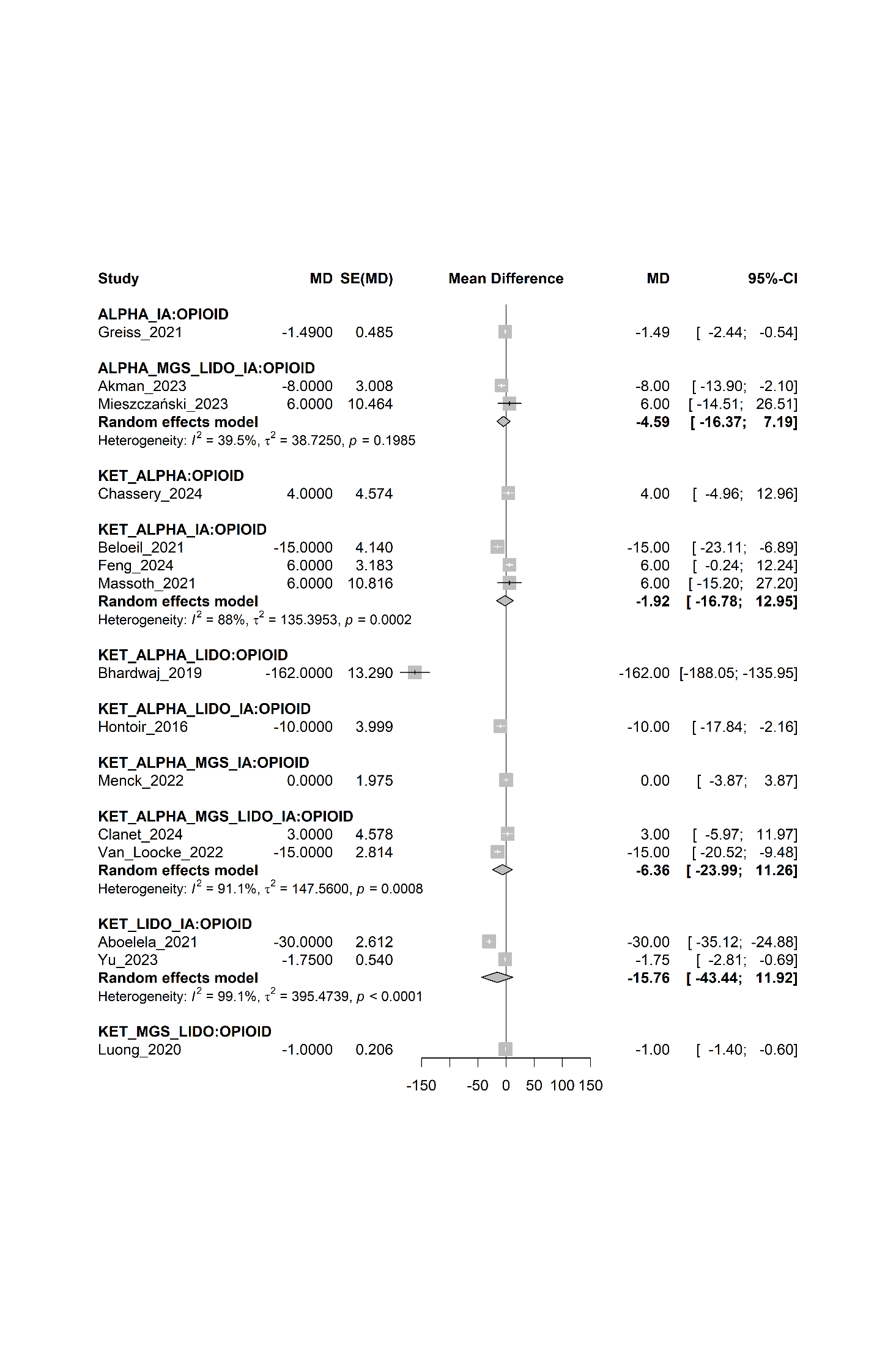


## Supplementary Figure 33: Postoperative opioid consumption – Forest plot of the pairwise meta-analysis


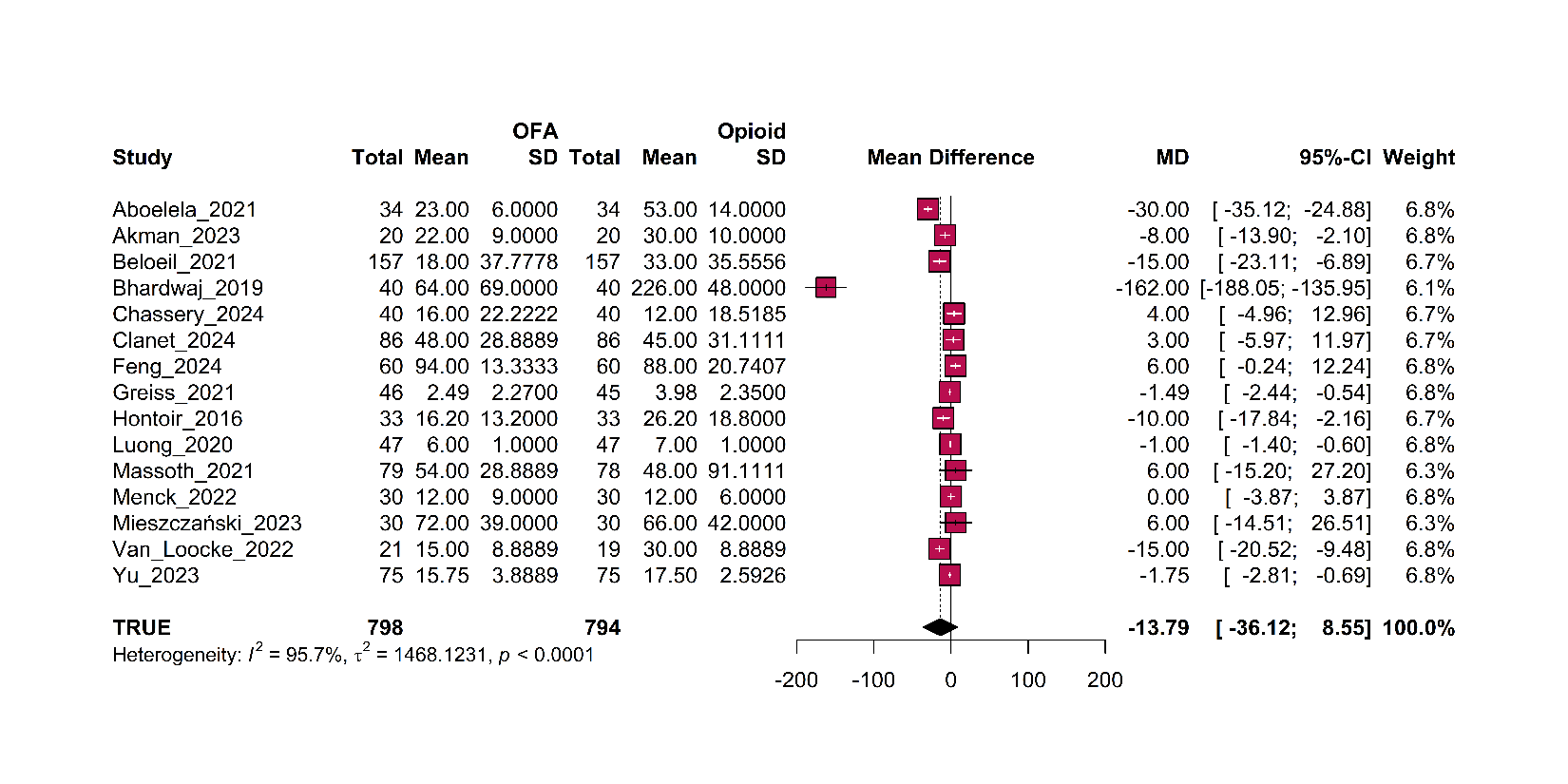


Supplementary Figure 34: Postoperative opioid consumption – Funnel plot of the pairwise meta-analysis


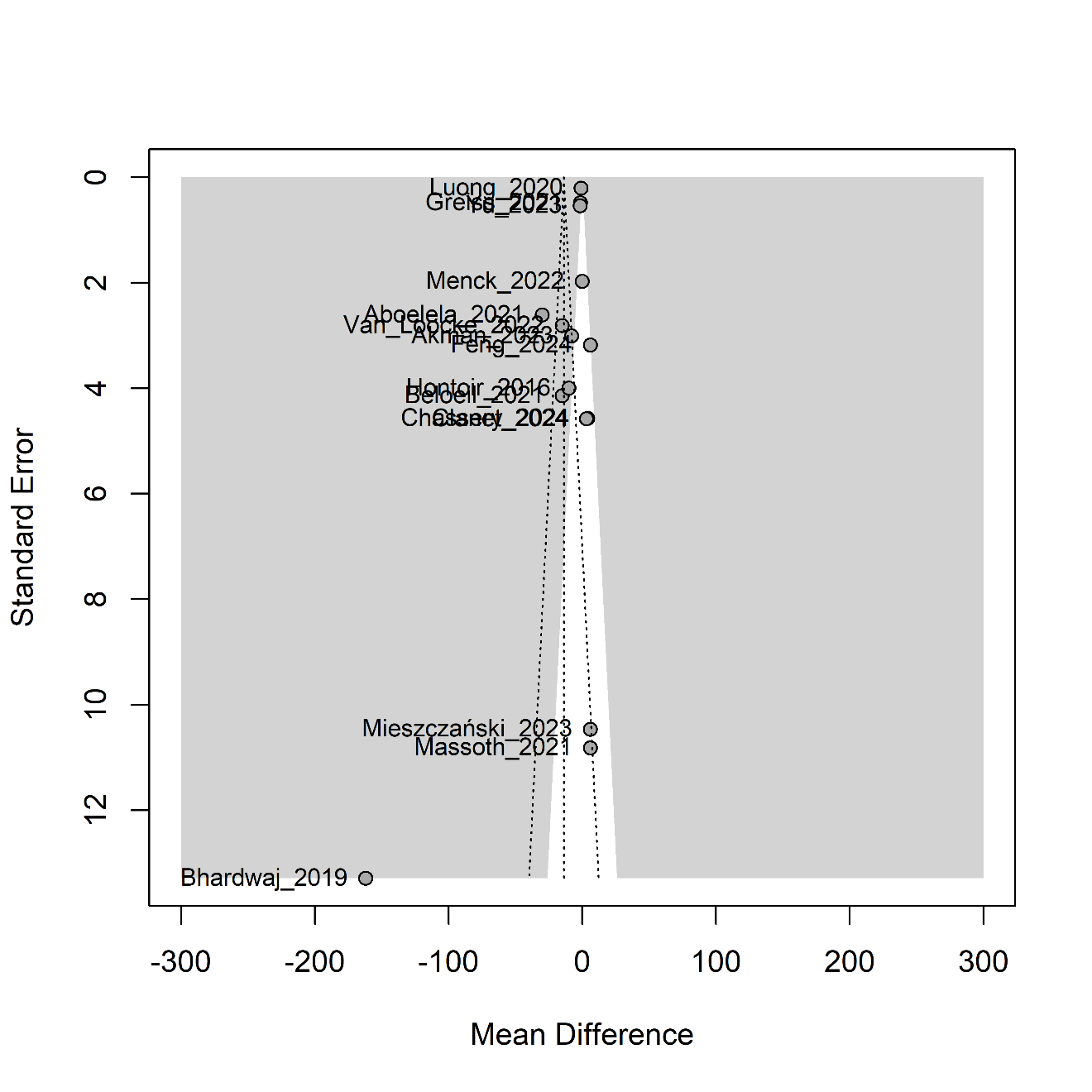


**Number of postoperative rescue analgesia requests: Supplementary Figures 35-44**

Supplementary Figure 35: Number of postoperative rescue analgesia requests – Network characteristics


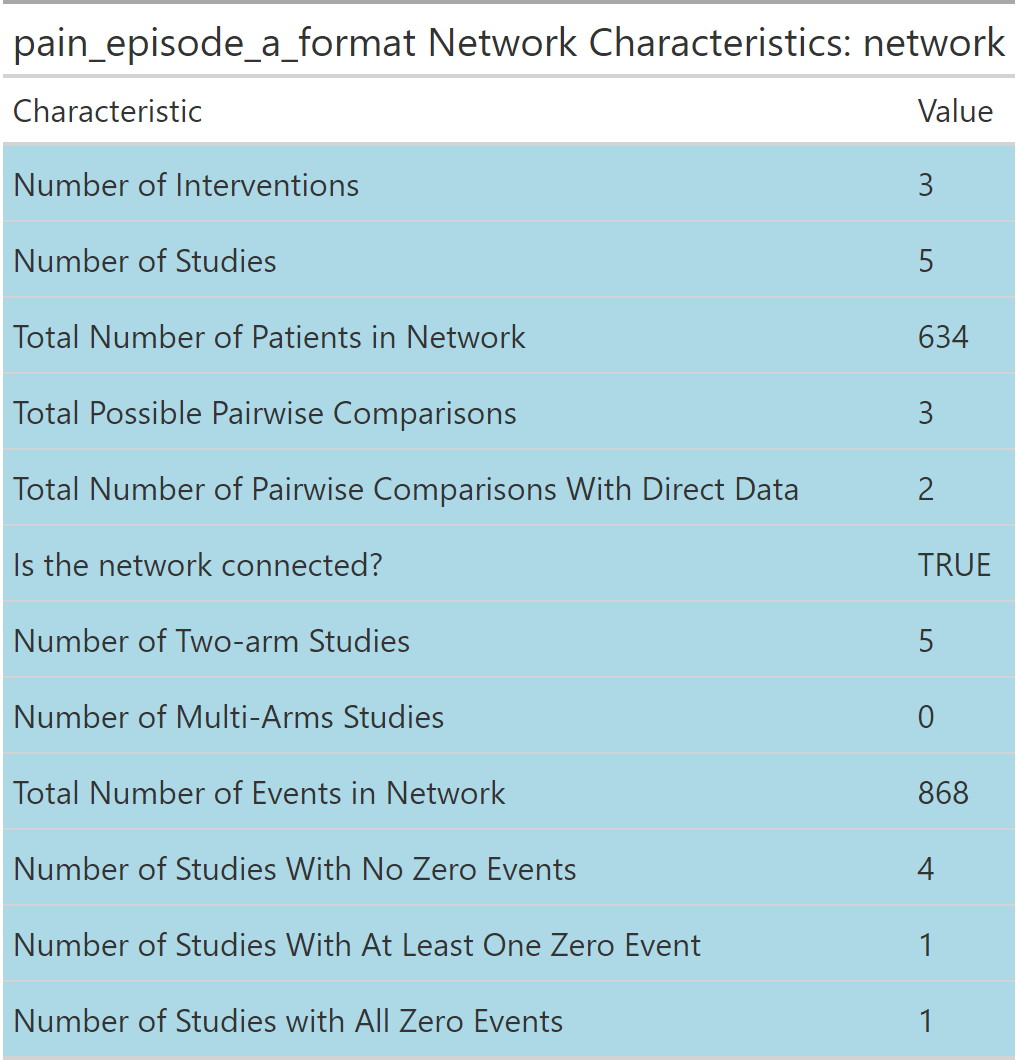


Supplementary Figure 36: Number of postoperative rescue analgesia requests – Characteristics of the interventions


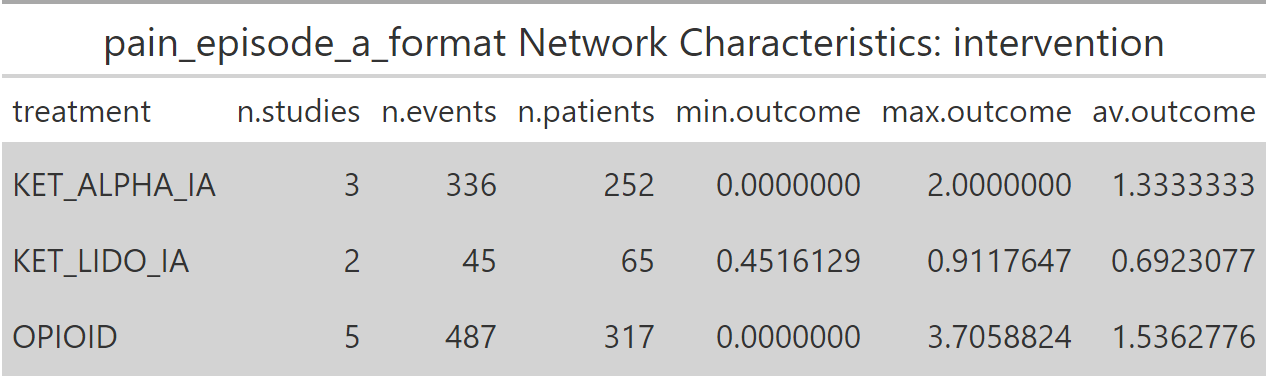


Supplementary Figure 37: Number of postoperative rescue analgesia requests – Characteristics of the comparisons


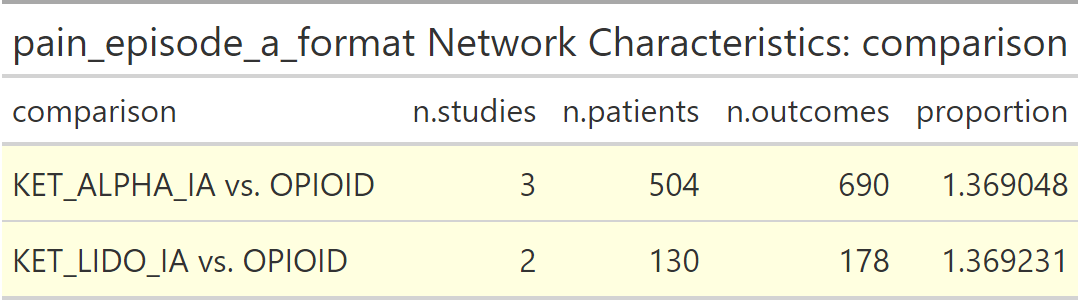


Supplementary Figure 38: Number of postoperative rescue analgesia requests – Network plot


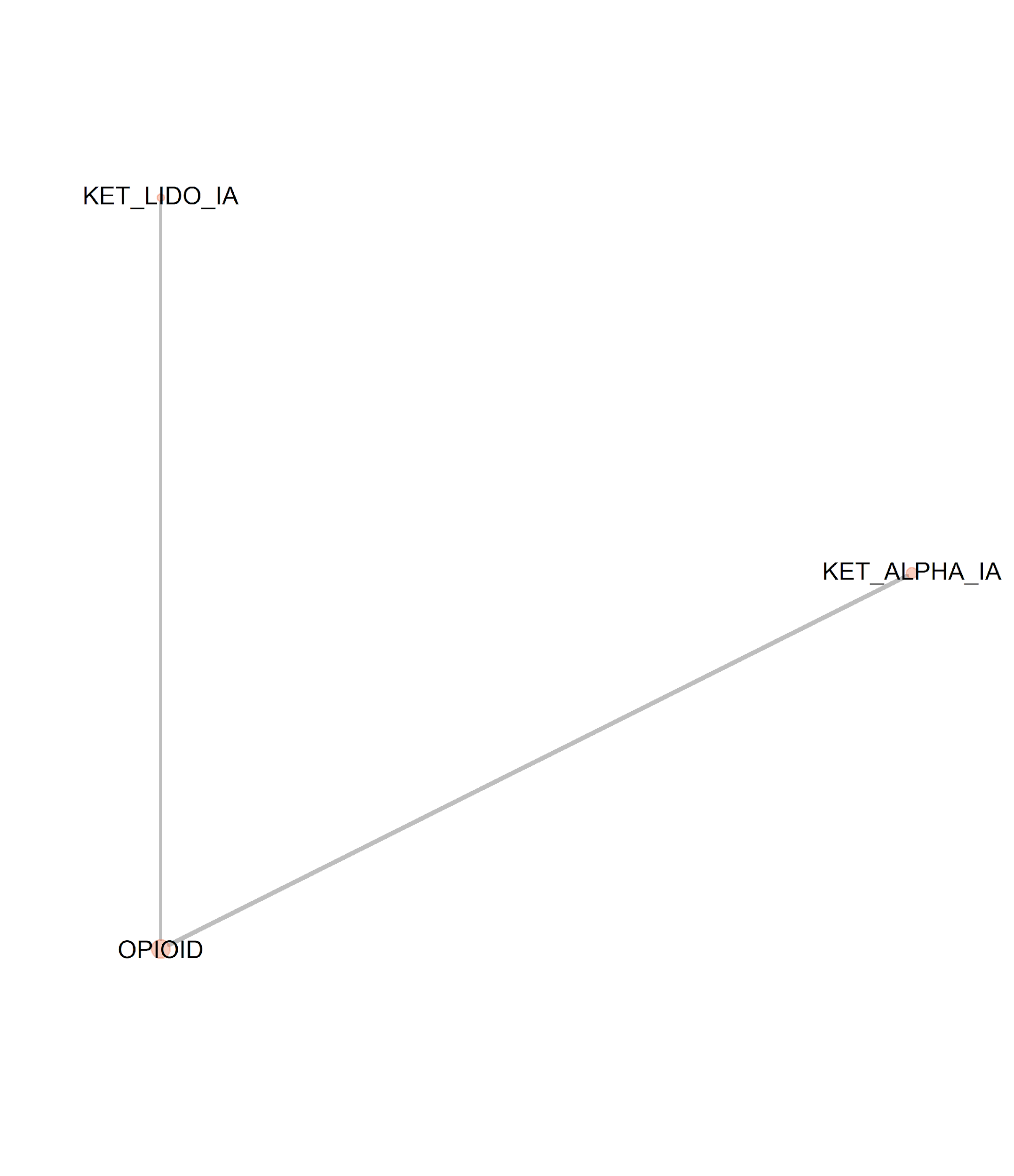


Supplementary Figure 39 Number of postoperative rescue analgesia requests – Heatplot


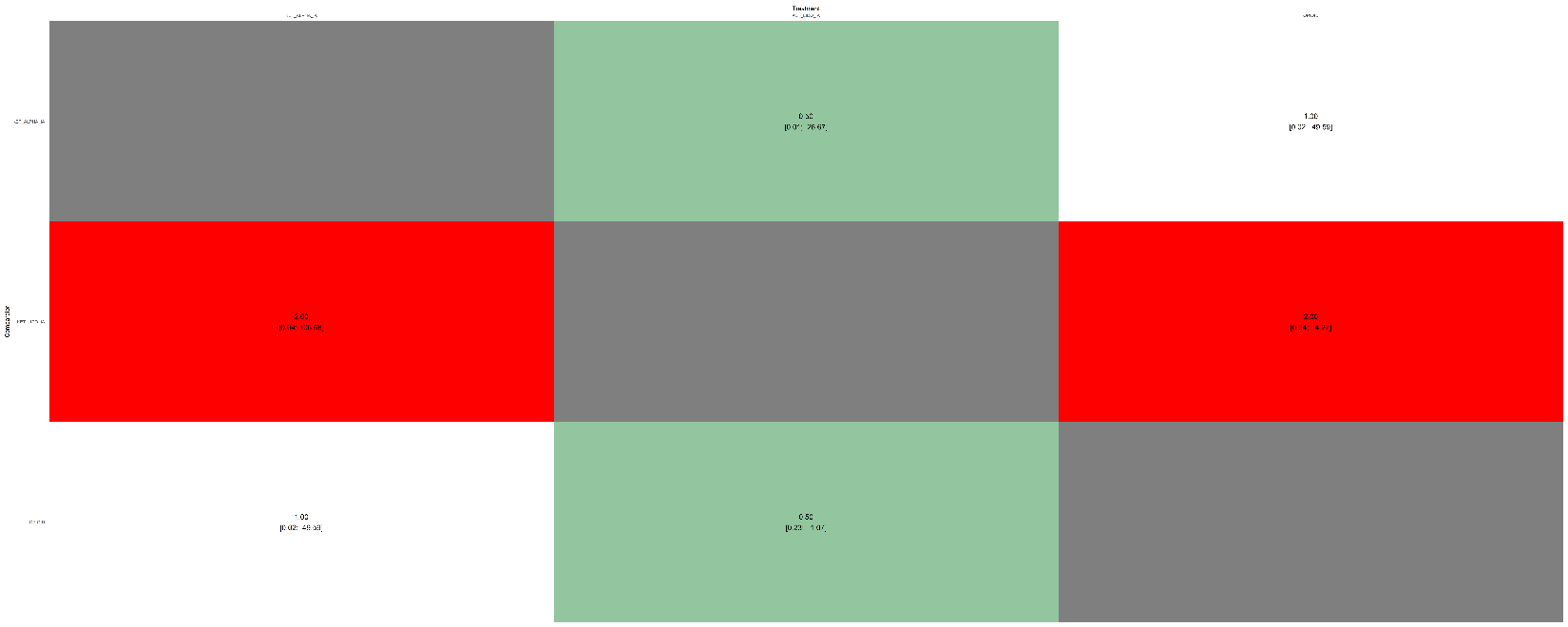


Supplementary Figure 40 Number of postoperative rescue analgesia requests – Rankplot


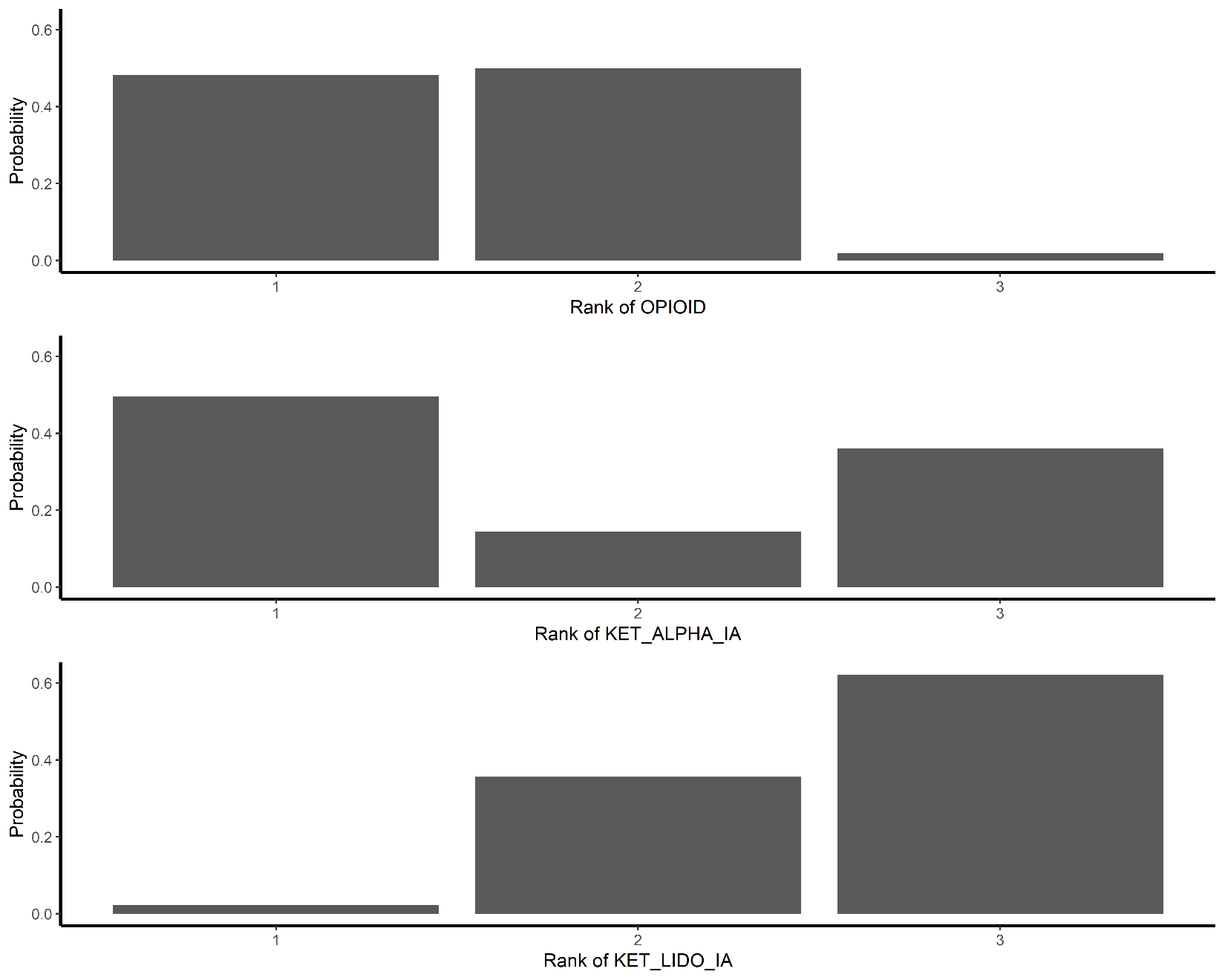


Supplementary Figure 41 Number of postoperative rescue analgesia requests – Nodesplit analysis


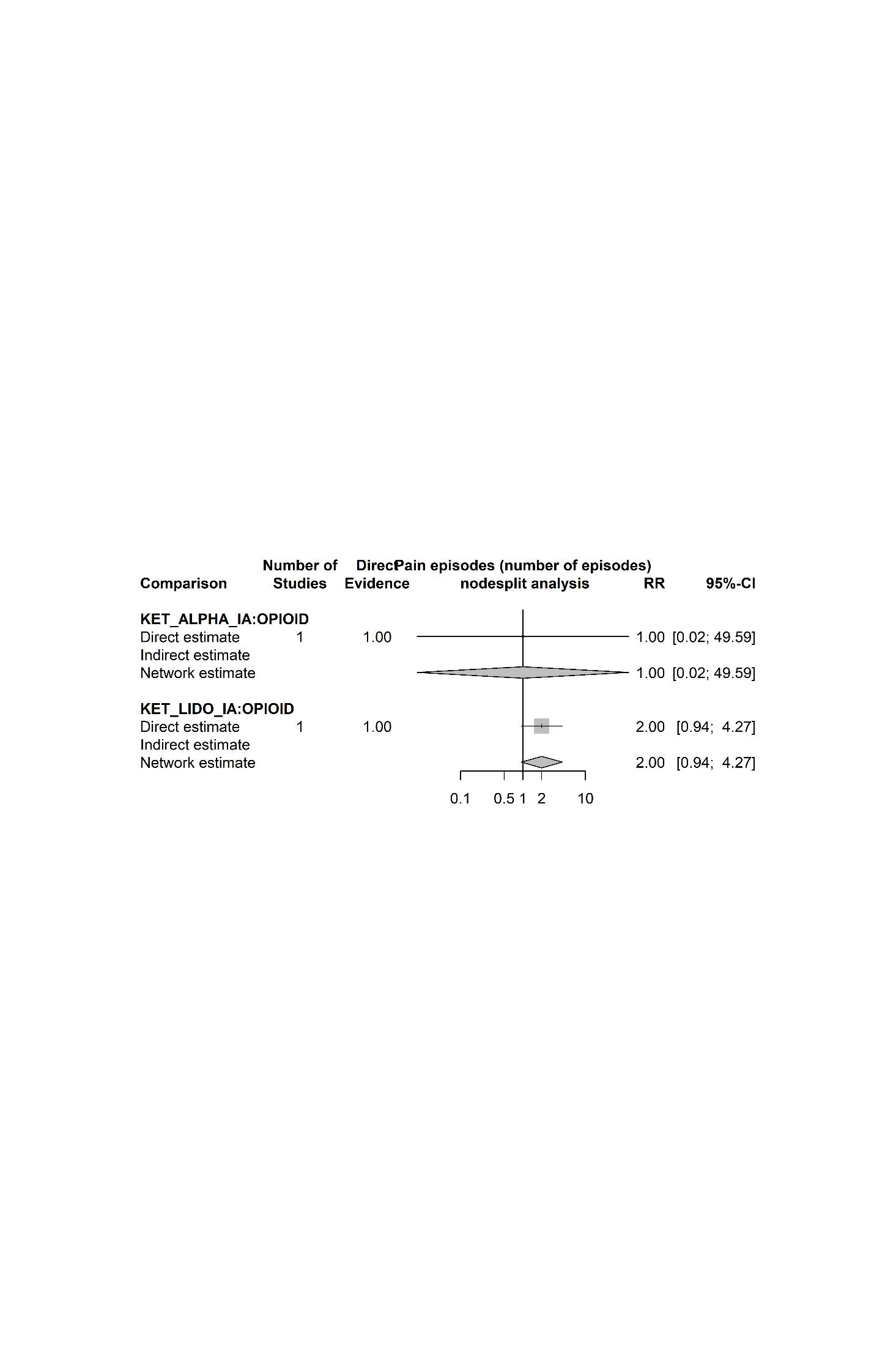


Supplementary Figure 42 Number of postoperative rescue analgesia requests – Result of the individual studies


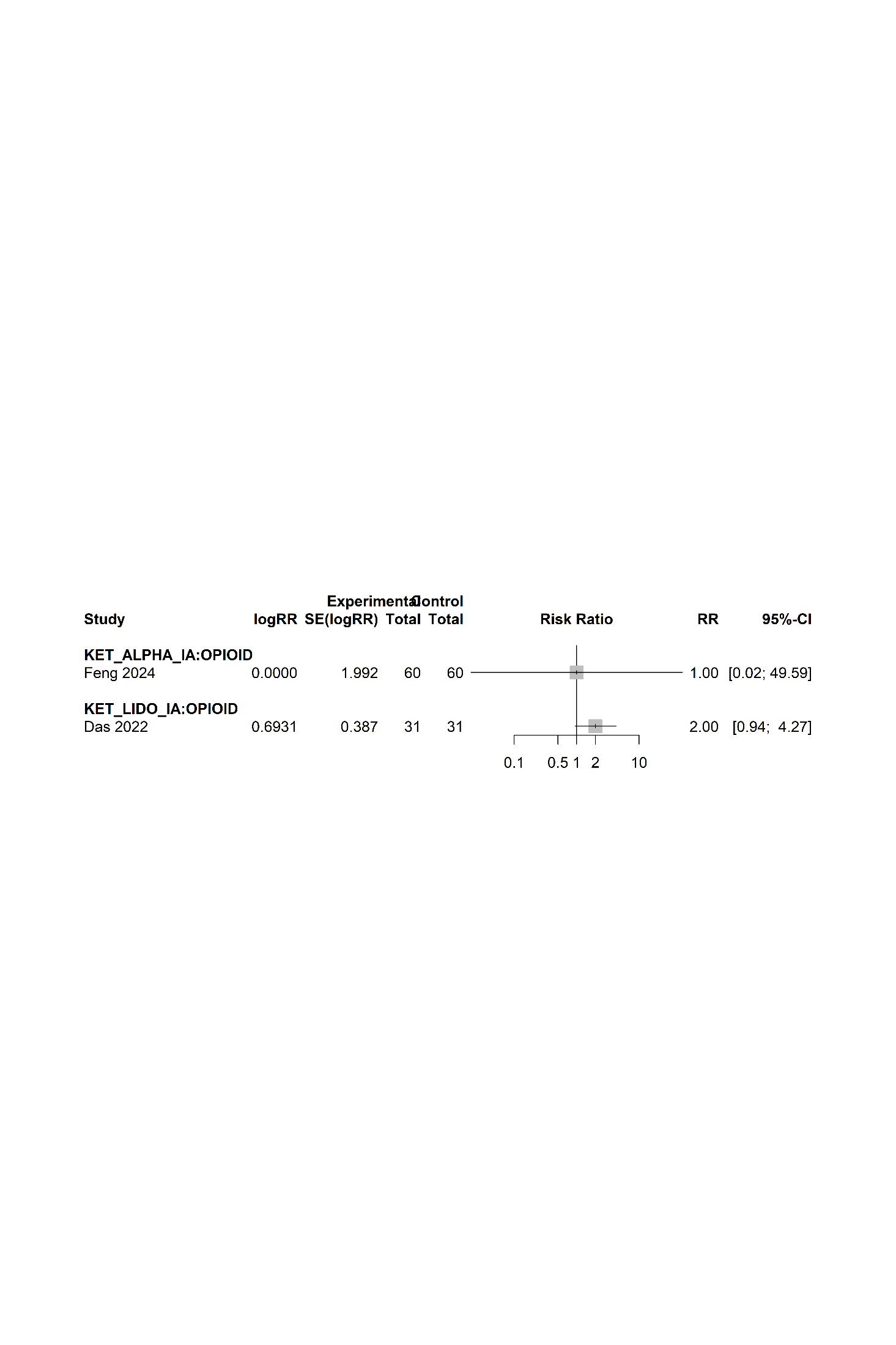


Supplementary Figure 43 Number of postoperative rescue analgesia requests – Forest plot of the pairwise meta-analysis


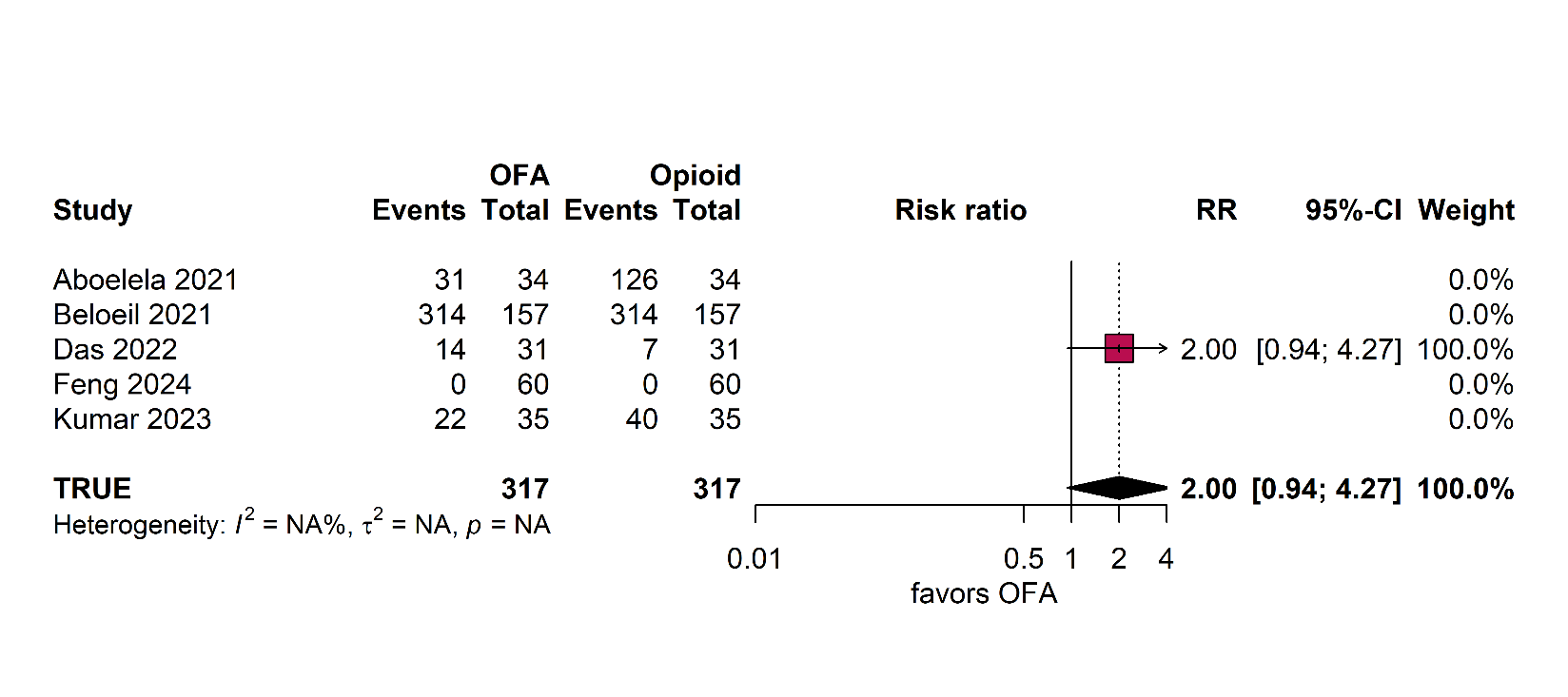


Supplementary Figure 44 Number of postoperative rescue analgesia requests – Funnel plot of the pairwise meta-analysis


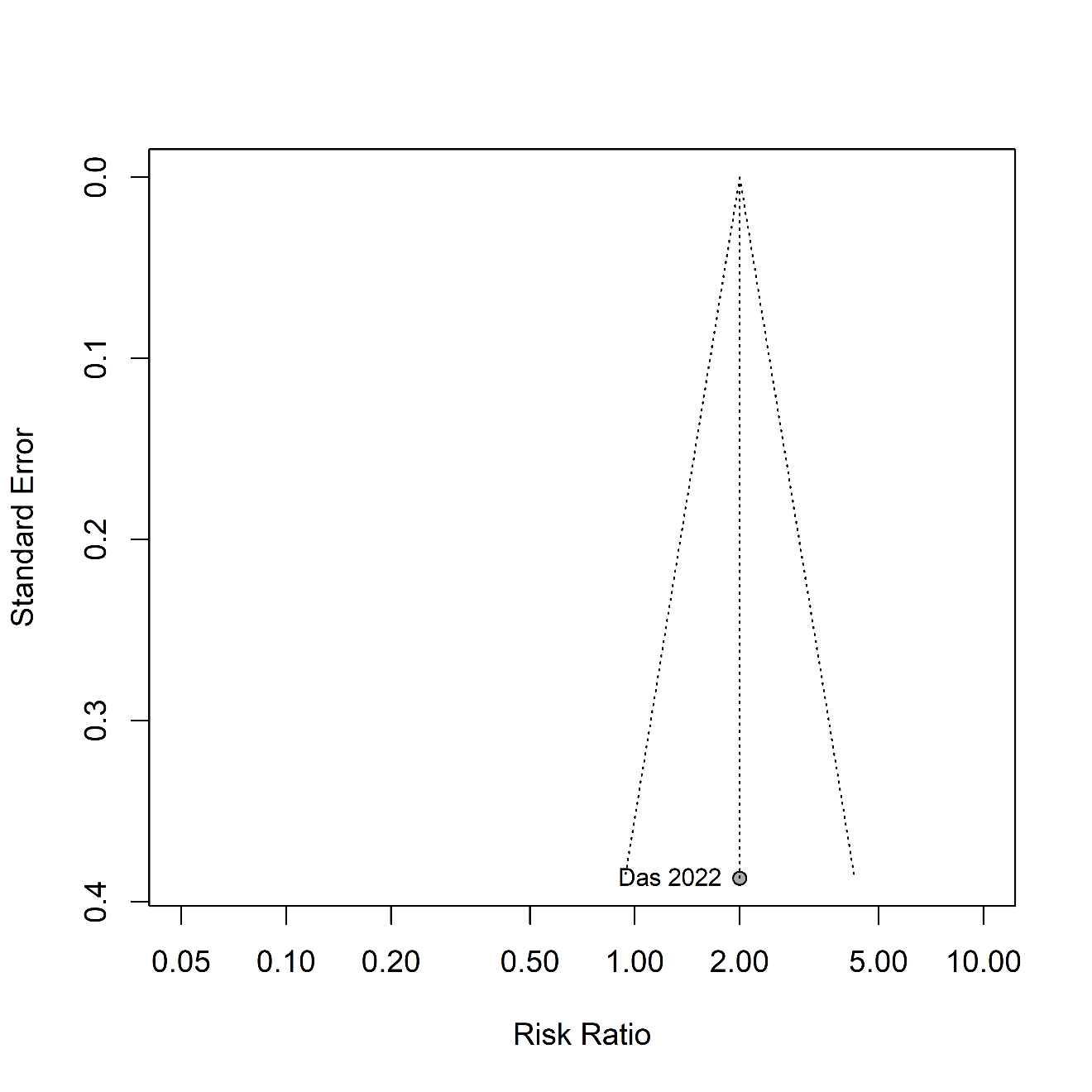


**Incidence of postoperative nausea or vomiting (PONV): Supplementary Figures 45-54**

Supplementary Figure 45: Incidence of postoperative nausea or vomiting (PONV) – Network characteristics


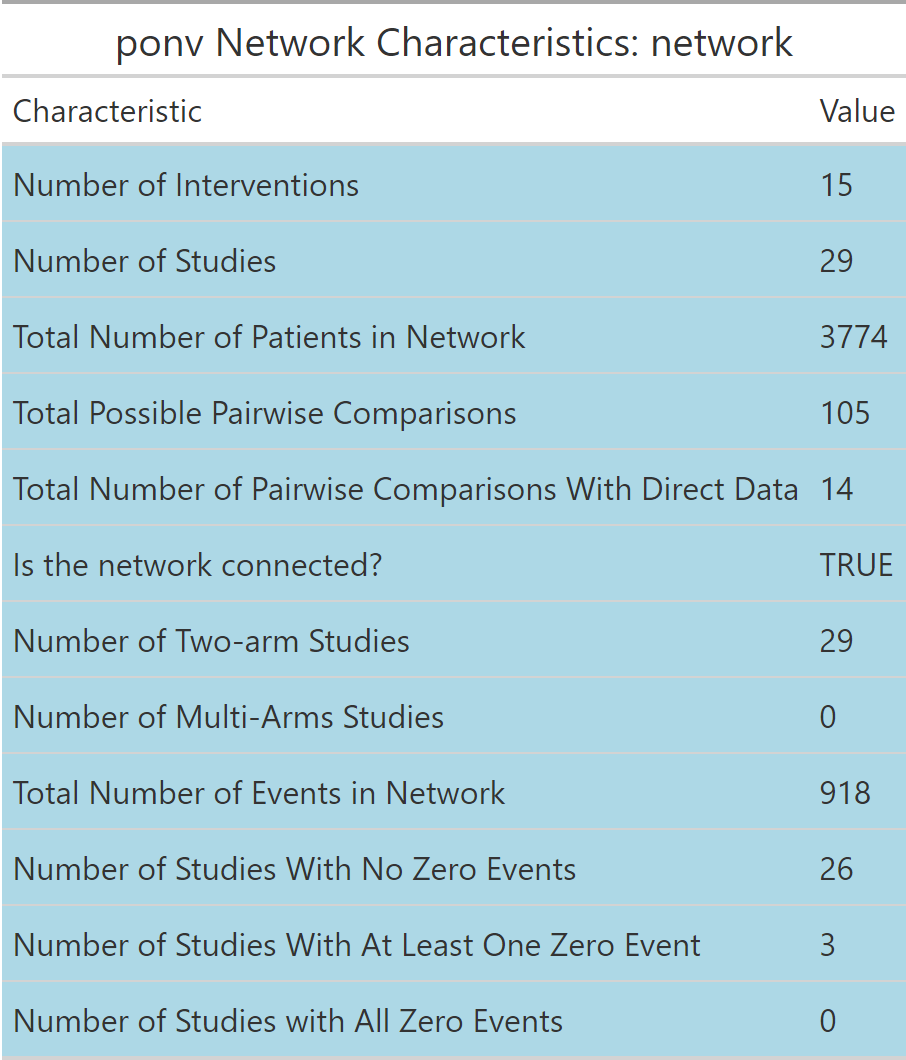


Supplementary Figure 46: Incidence of postoperative nausea or vomiting (PONV) – Characteristics of the interventions


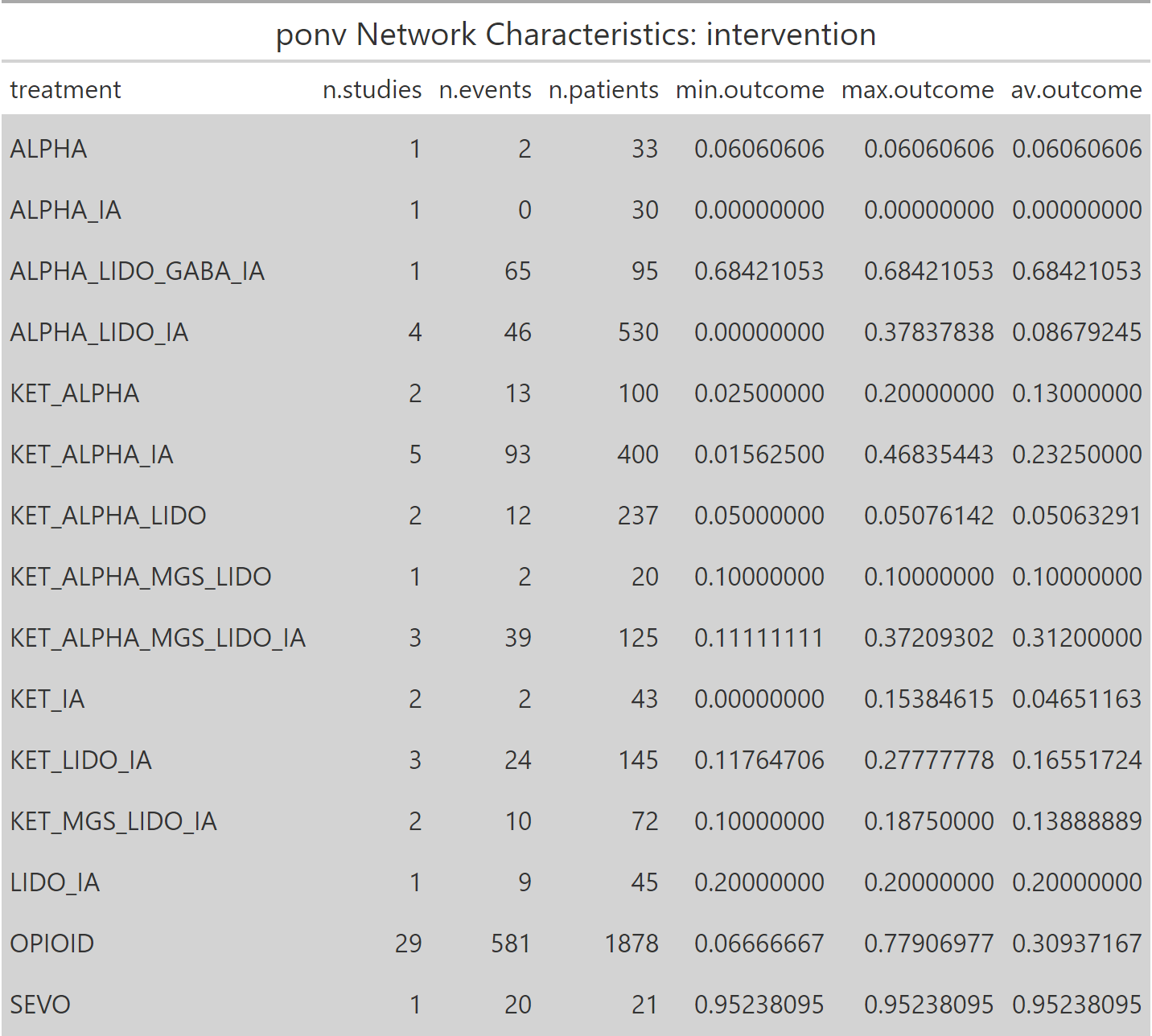


Supplementary Figure 47: Incidence of postoperative nausea or vomiting (PONV) – Characteristics of the comparisons


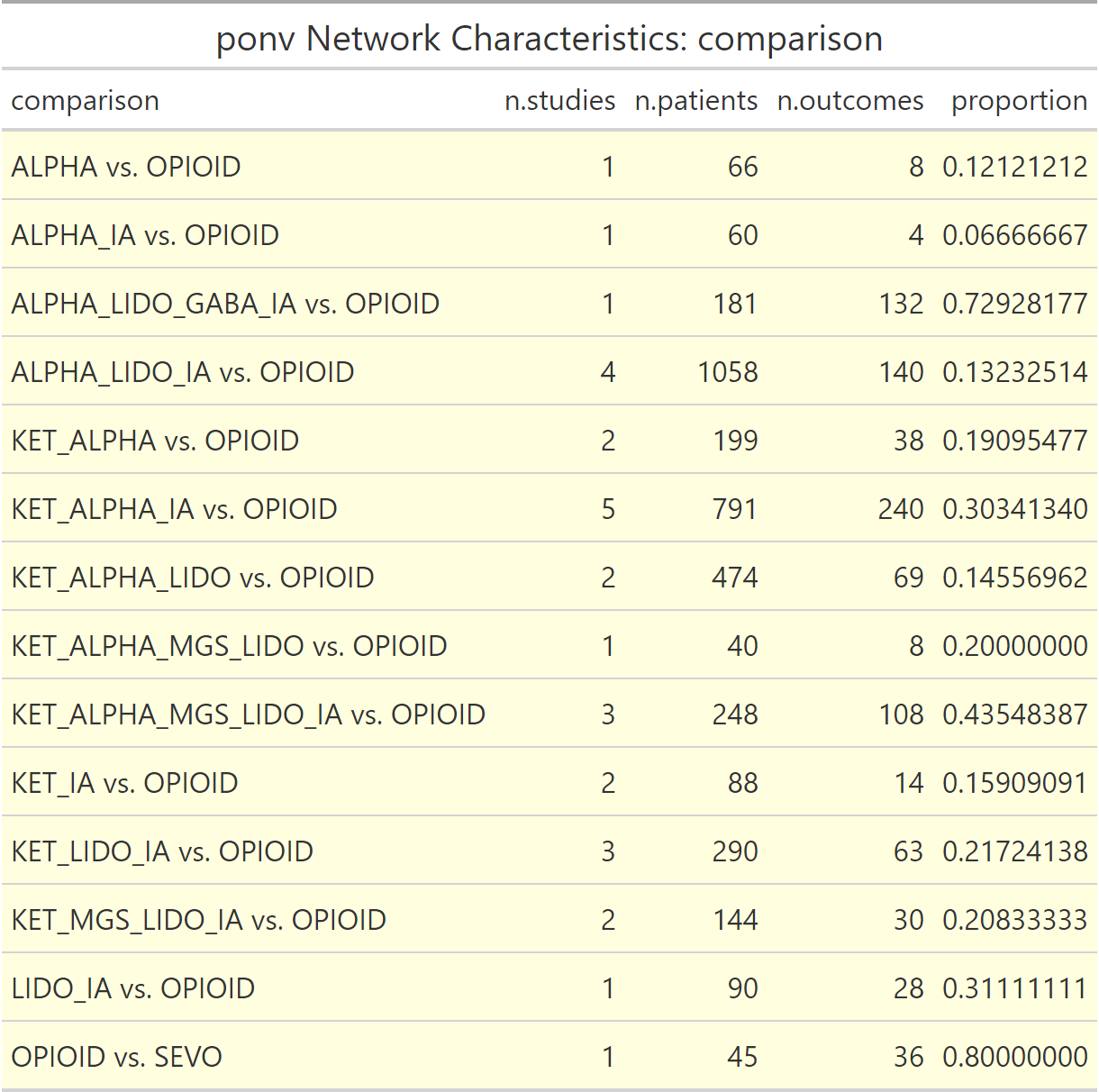


Supplementary Figure 48: Incidence of postoperative nausea or vomiting (PONV) – Network plot


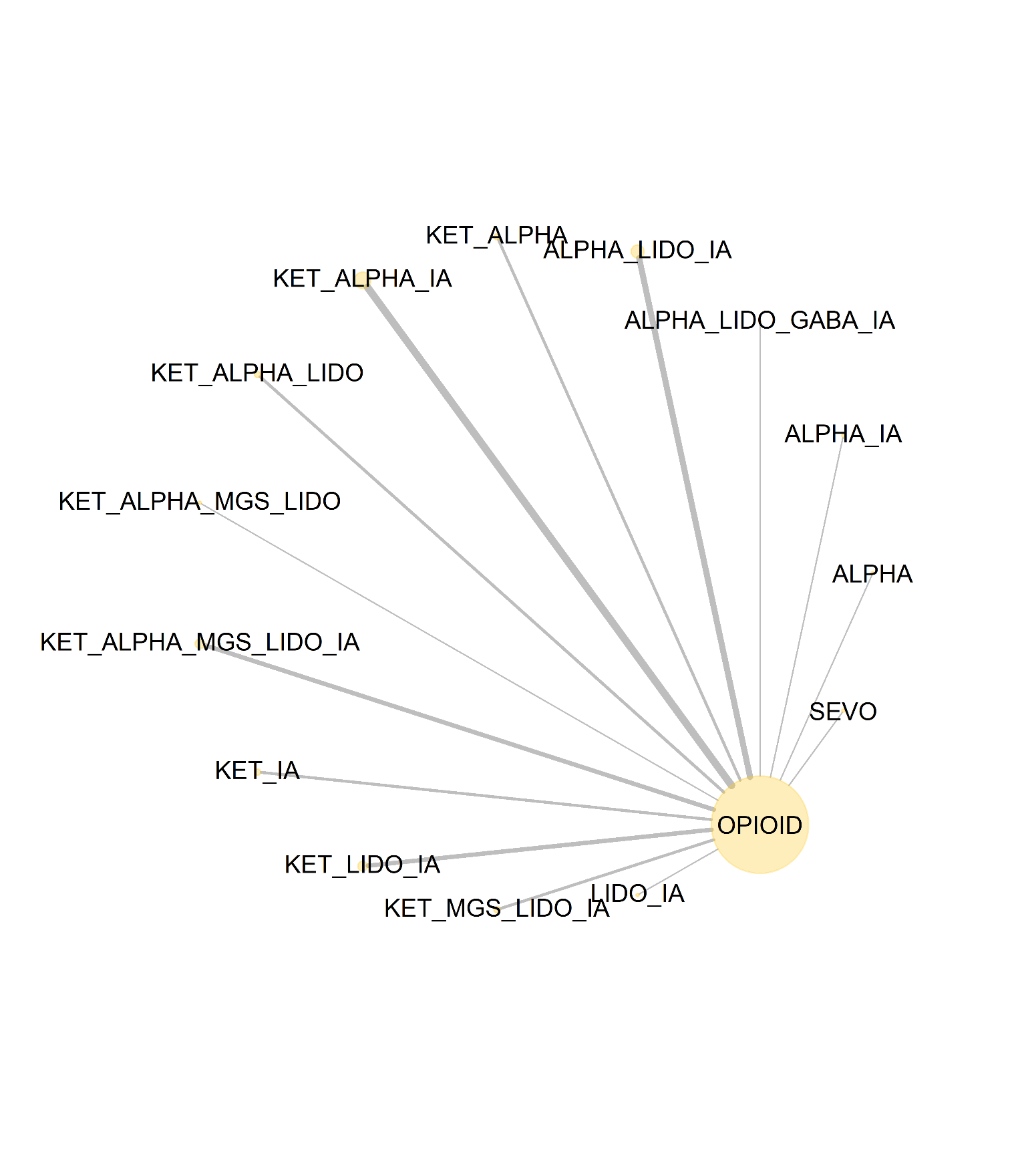


Supplementary Figure 49 Incidence of postoperative nausea or vomiting (PONV) – Heatplot


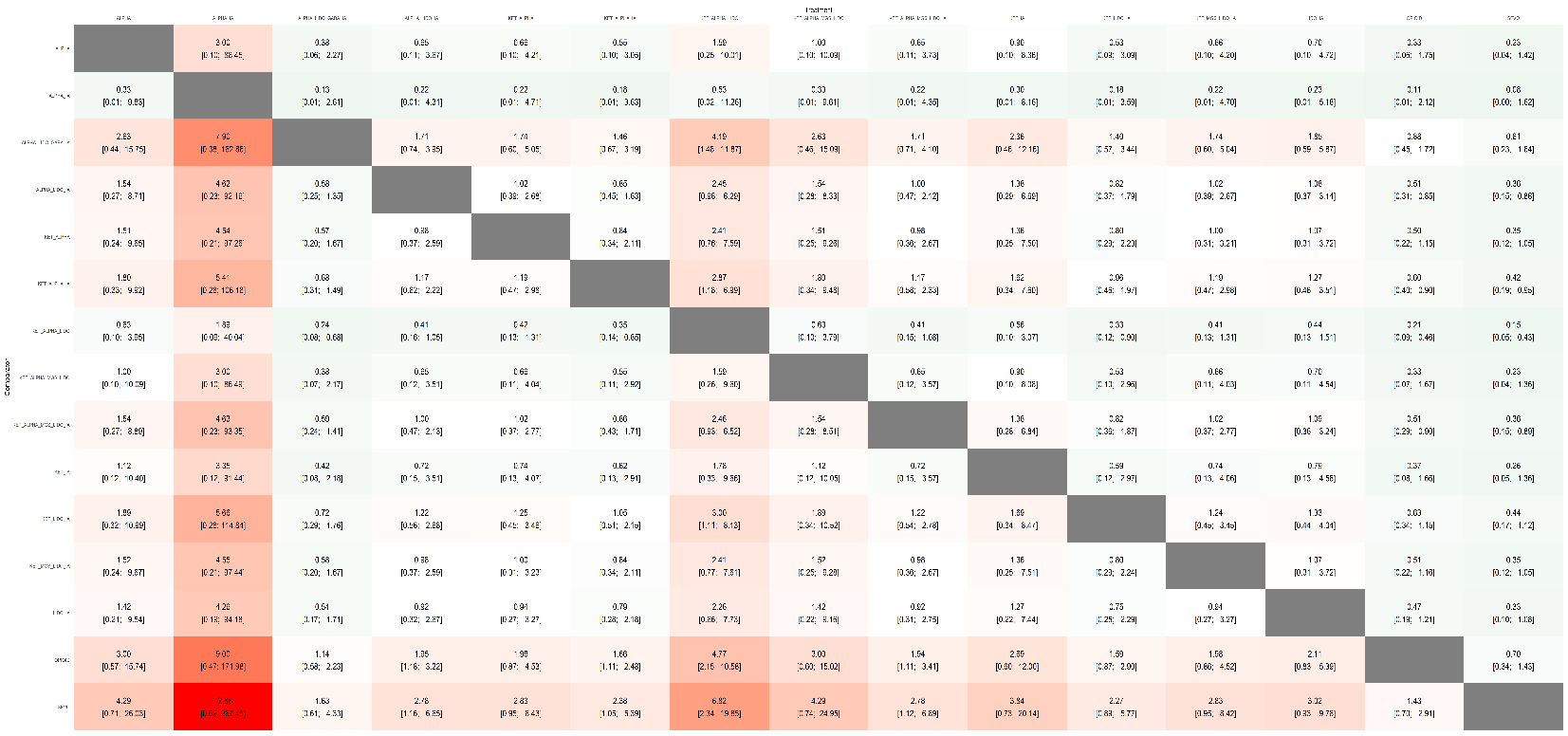


Supplementary Figure 50 Incidence of postoperative nausea or vomiting (PONV) – Rankplot


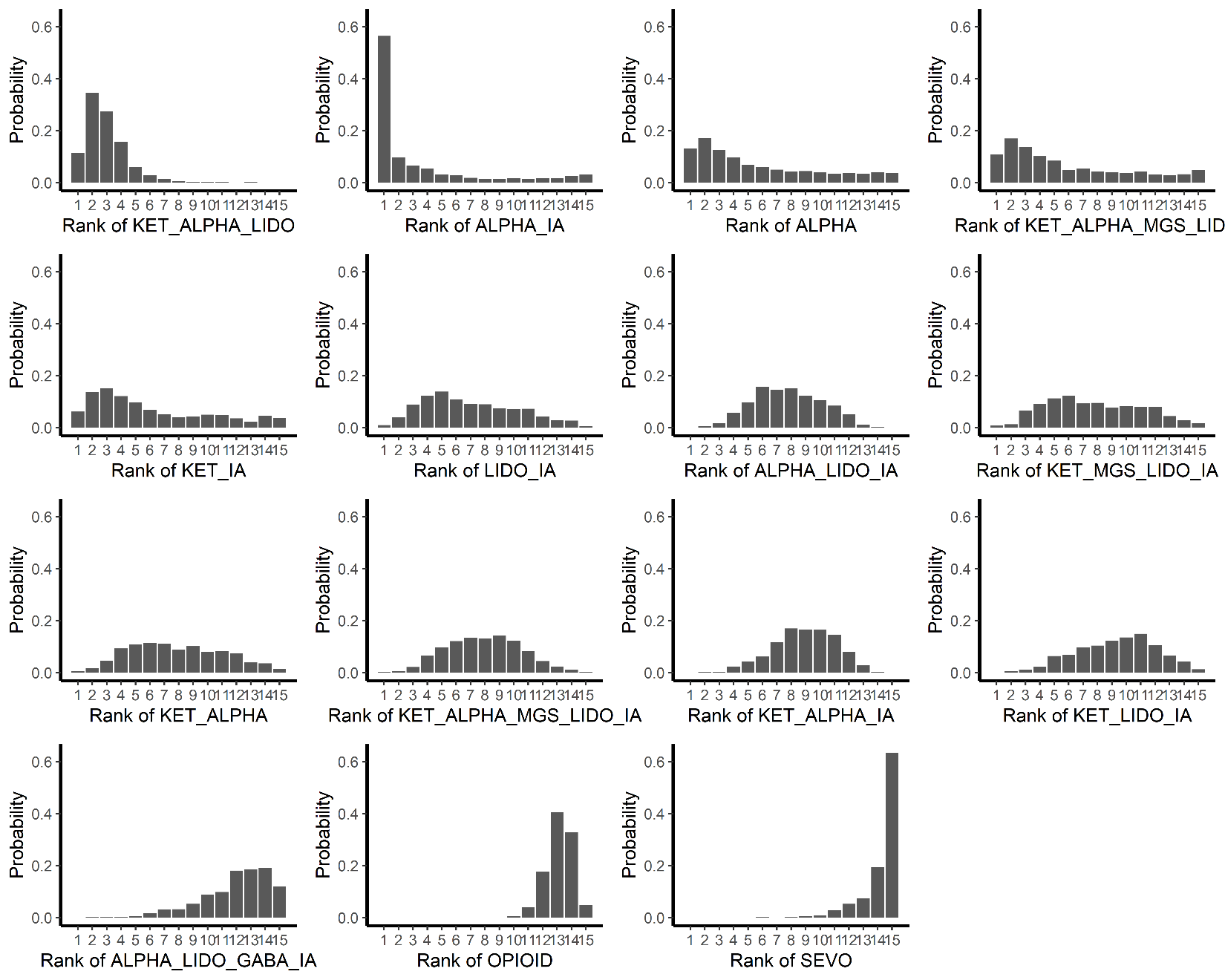


Supplementary Figure 51 Incidence of postoperative nausea or vomiting (PONV) – Nodesplit analysis


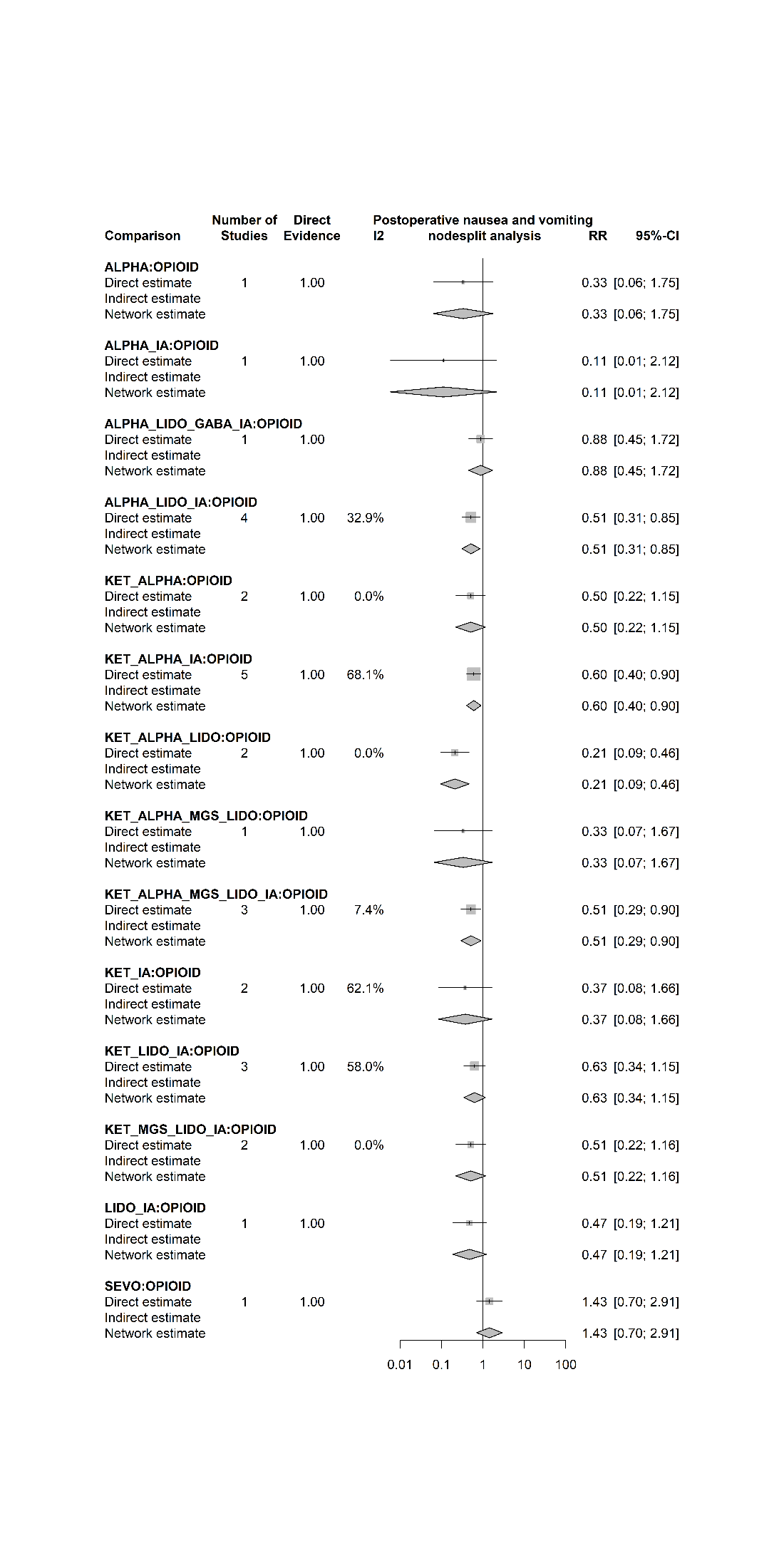


Supplementary Figure 52 Incidence of postoperative nausea or vomiting (PONV) – Results of the individual studies


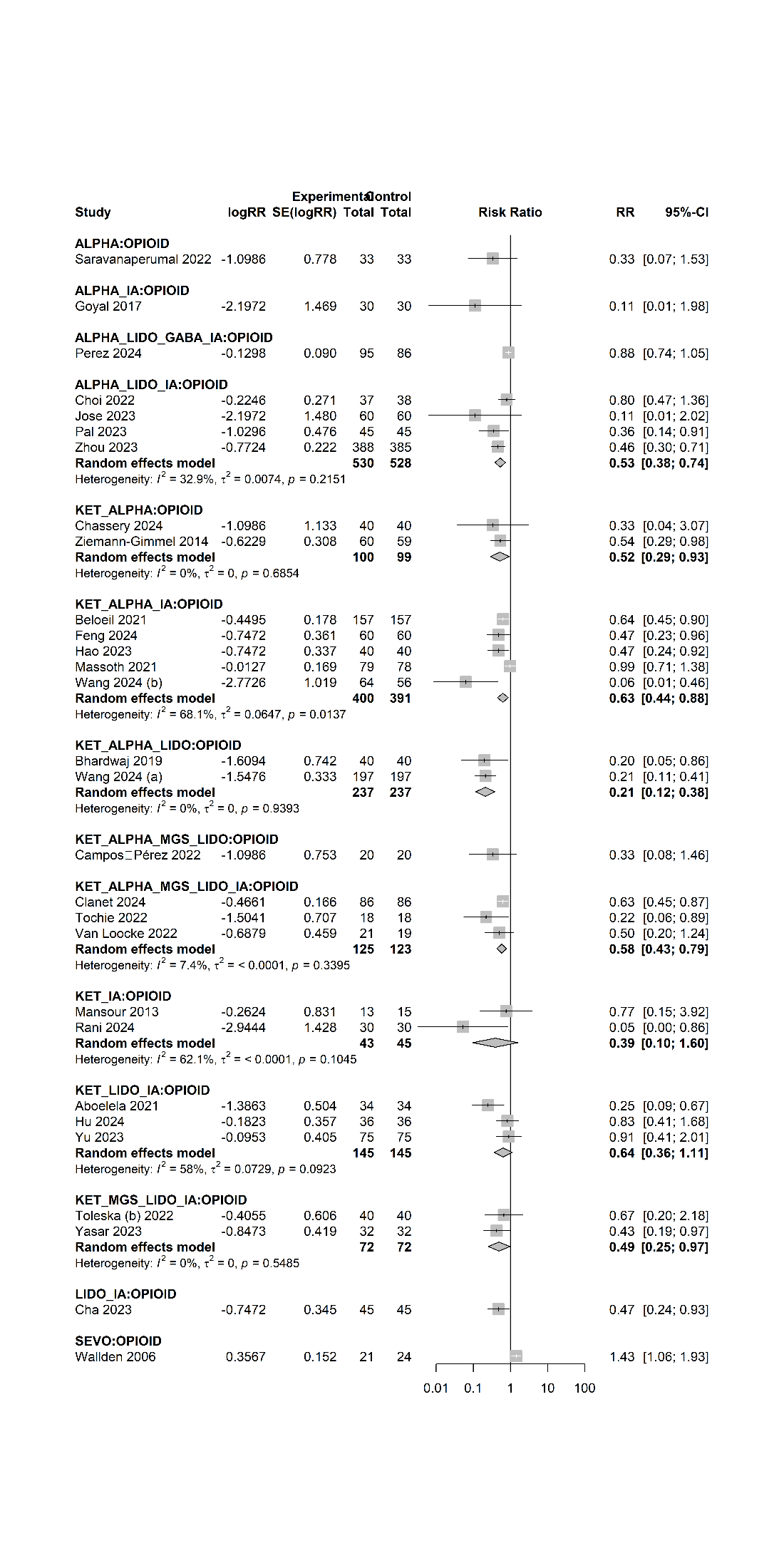


Supplementary Figure 53 Incidence of postoperative nausea or vomiting (PONV) – Forest plot of the pairwise meta-analysis


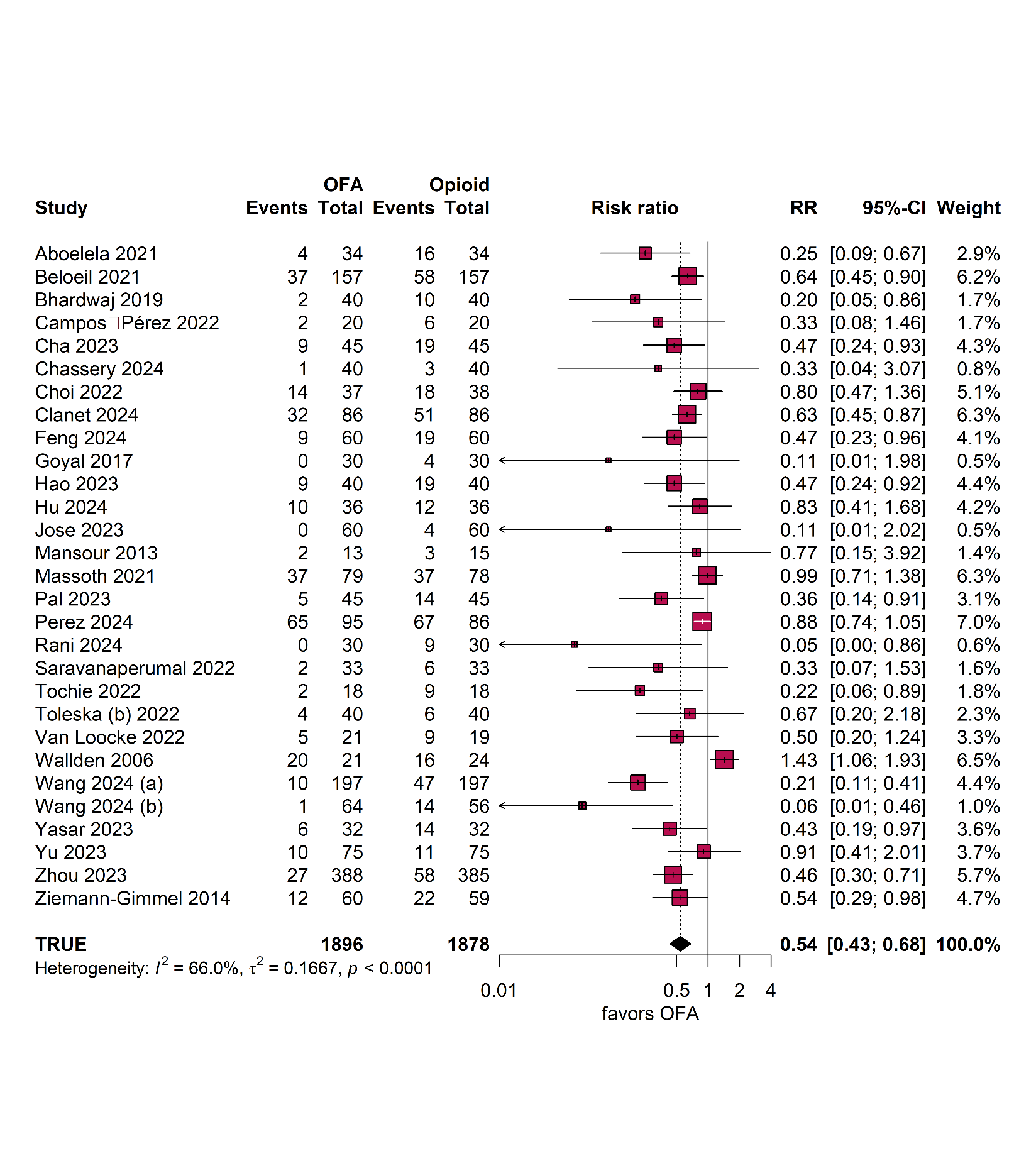


Supplementary Figure 54 Incidence of postoperative nausea or vomiting (PONV) – Funnel plot of the pairwise meta-analysis


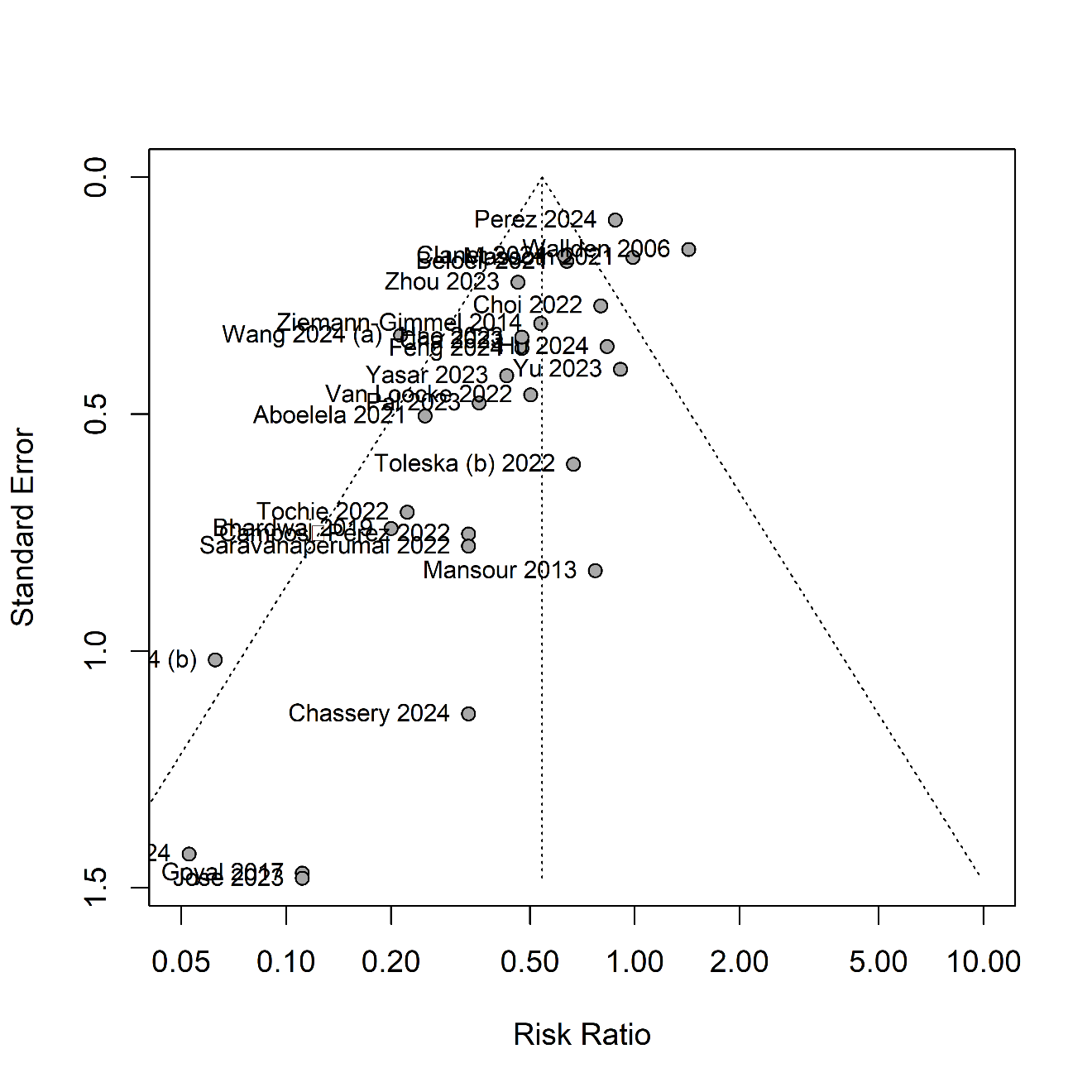


**Duration of hospital stay: Supplementary Figures 55-63**

Supplementary Figure 55: Duration of hospital stay – Network characteristics


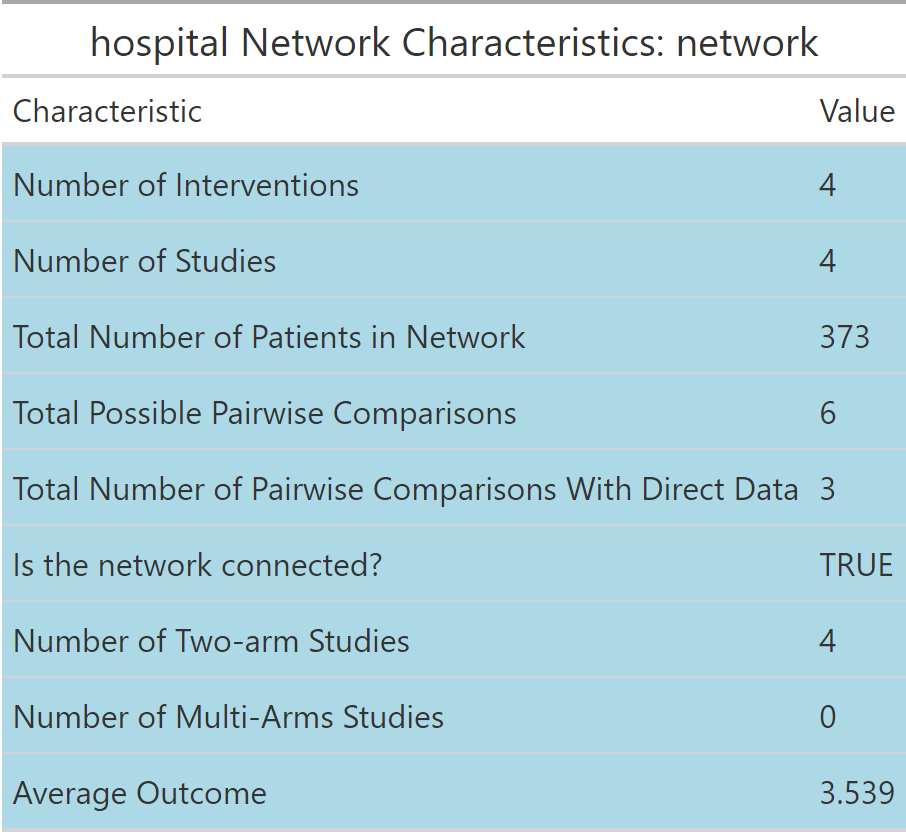


Supplementary Figure 56: Duration of hospital stay – Characteristics of the interventions


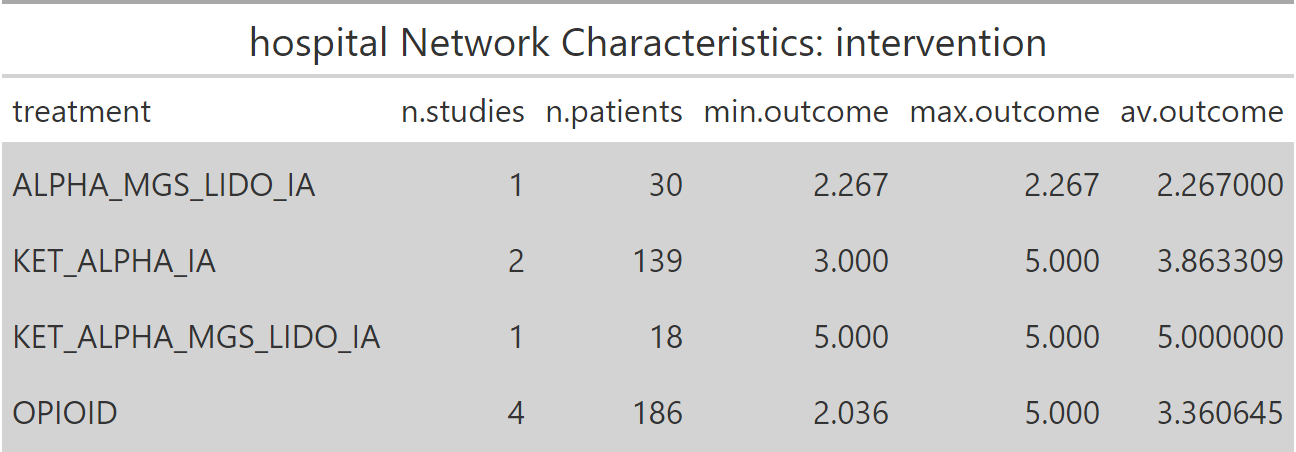


Supplementary Figure 57: Duration of hospital stay – Characteristics of the comparisons


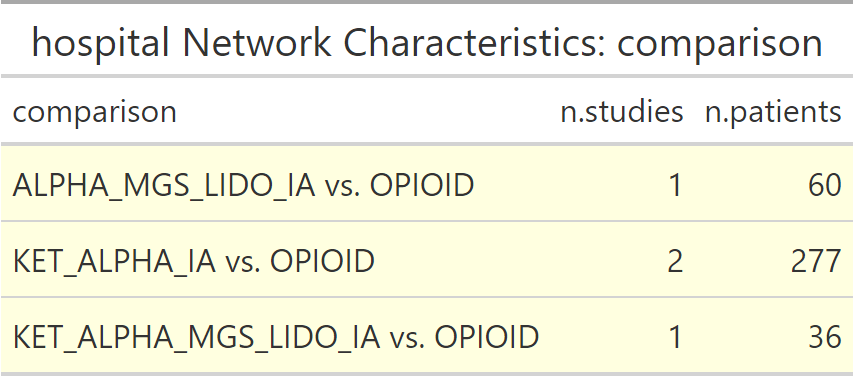


Supplementary Figure 58: Duration of hospital stay – Network plot


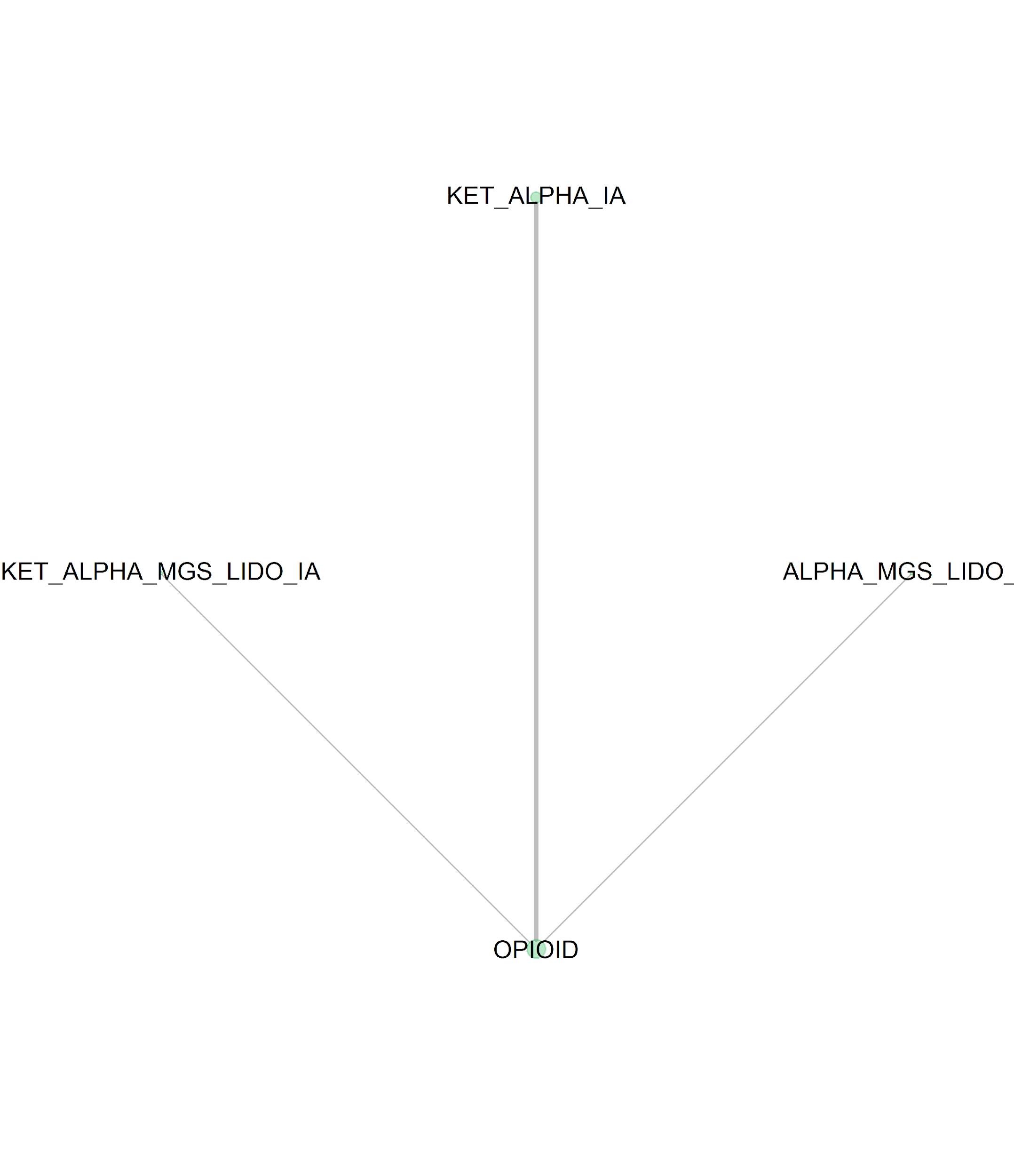


Supplementary Figure 59 Duration of hospital stay – Heatplot


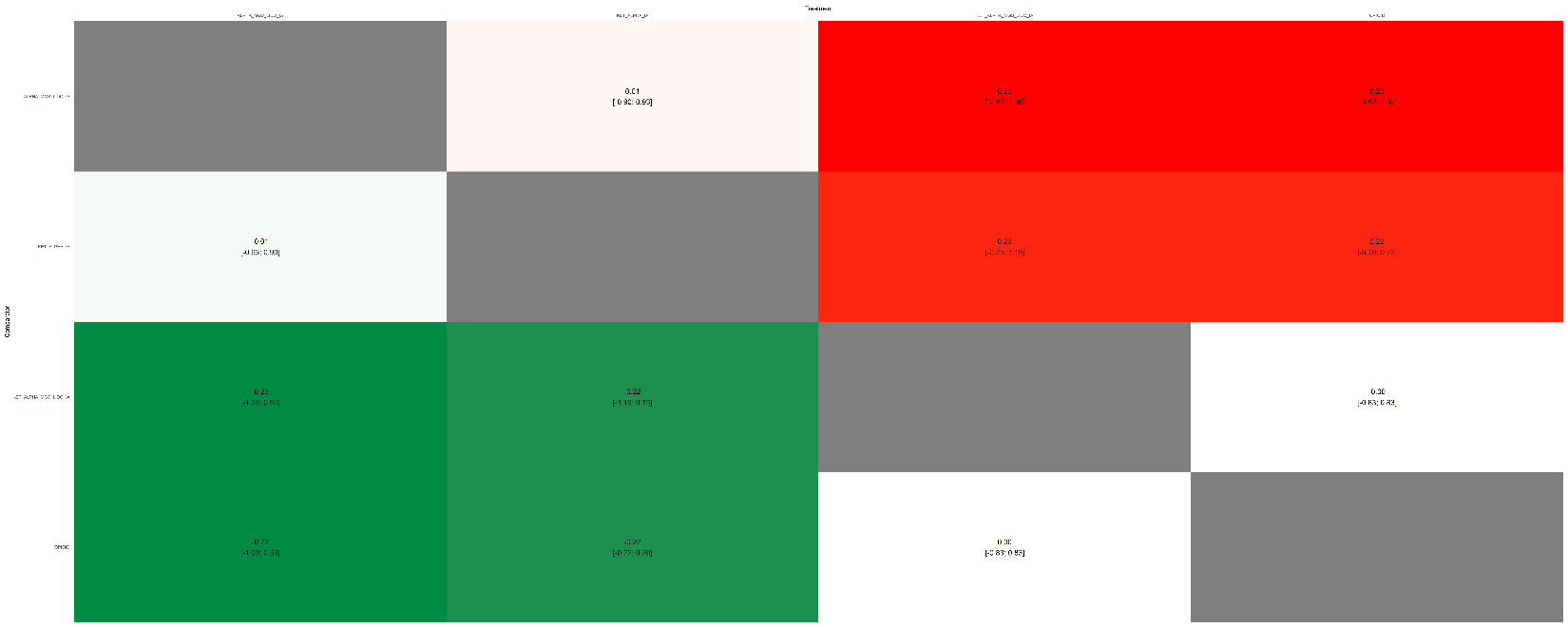


Supplementary Figure 60 Duration of hospital stay – Rankplot


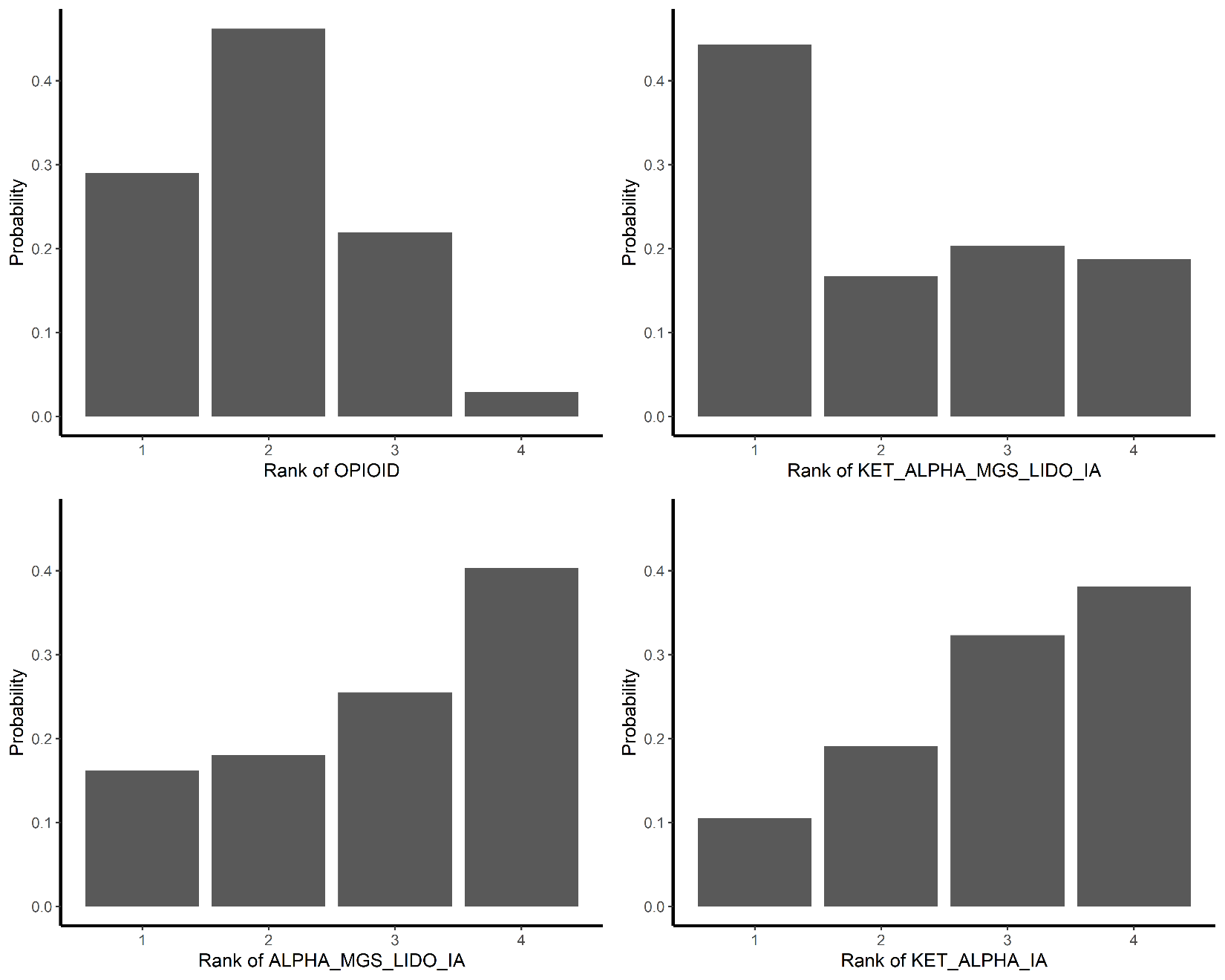


Supplementary Figure 61 Duration of hospital stay – Nodesplit analysis


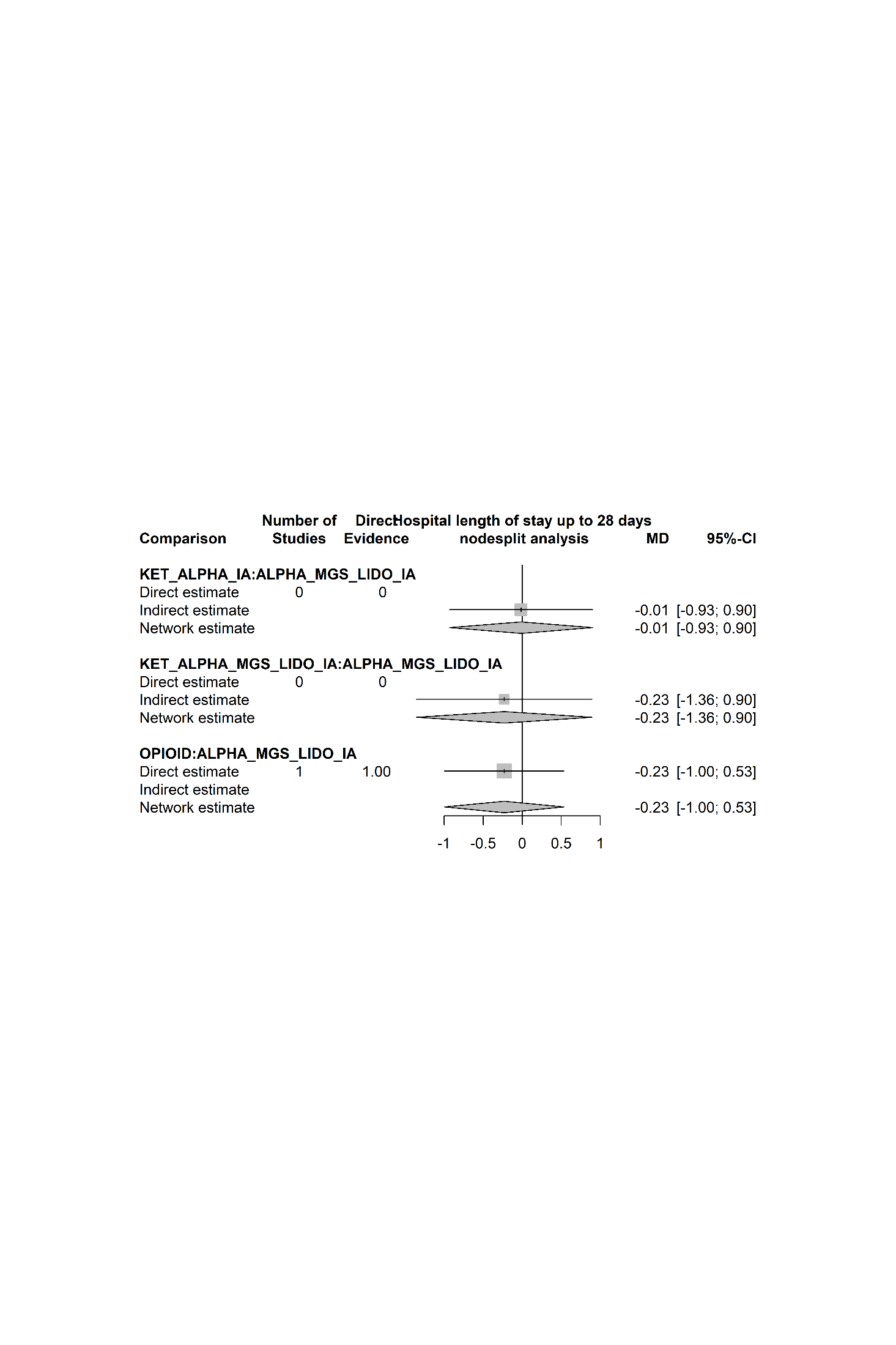


Supplementary Figure 61 Duration of hospital stay – Result of the individual studies


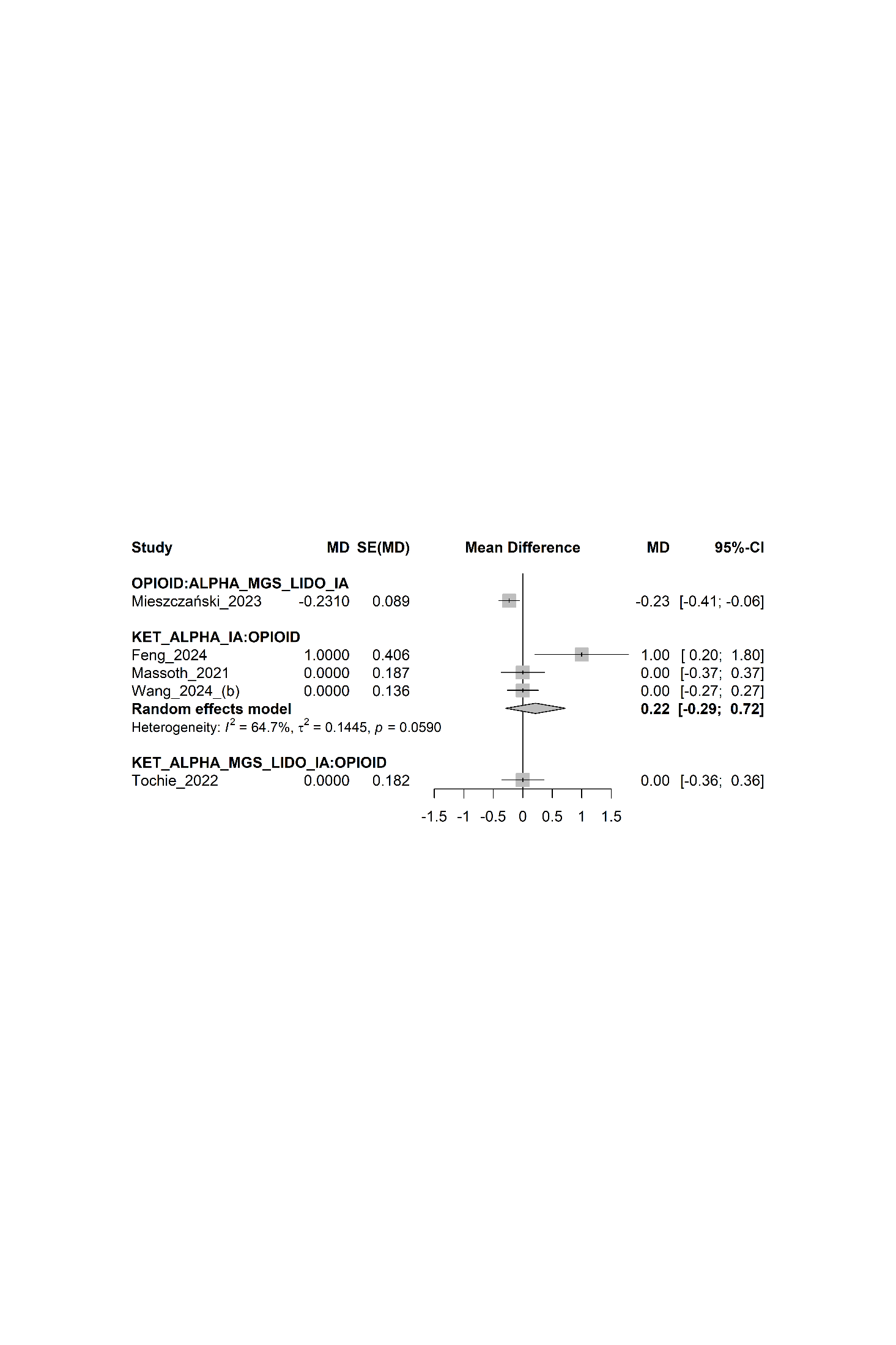


Supplementary Figure 62 Duration of hospital stay – Forest plot of the pairwise meta-analysis


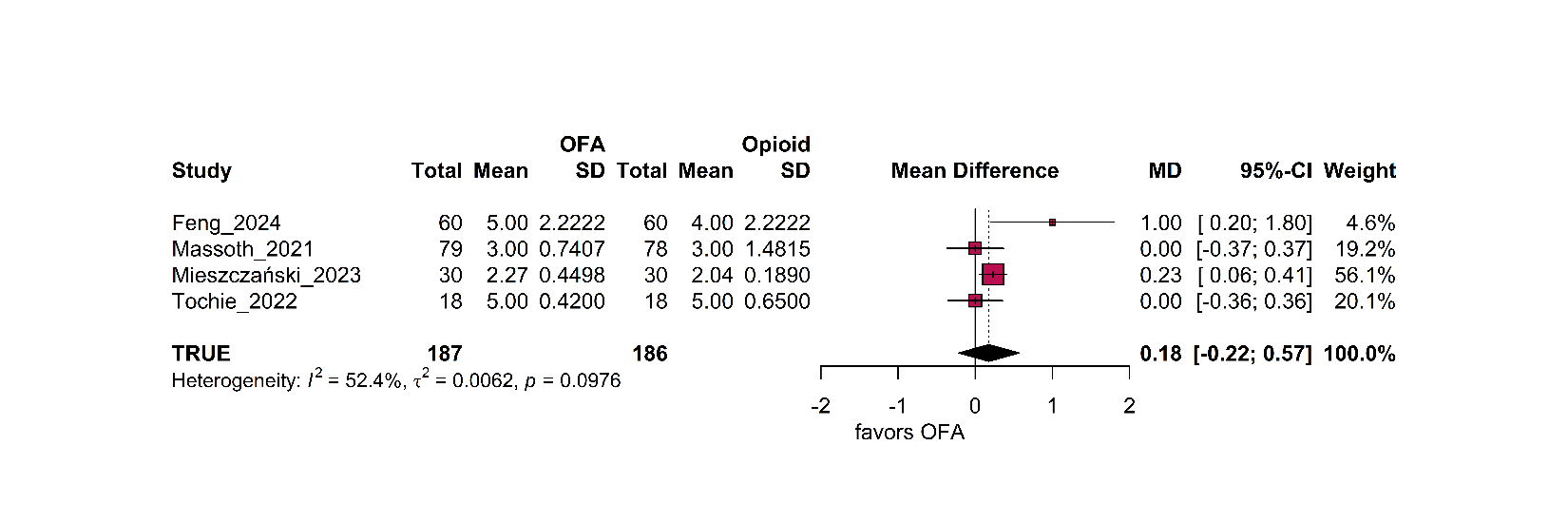


Supplementary Figure 63 Duration of hospital stay – Funnel plot of the pairwise meta-analysis


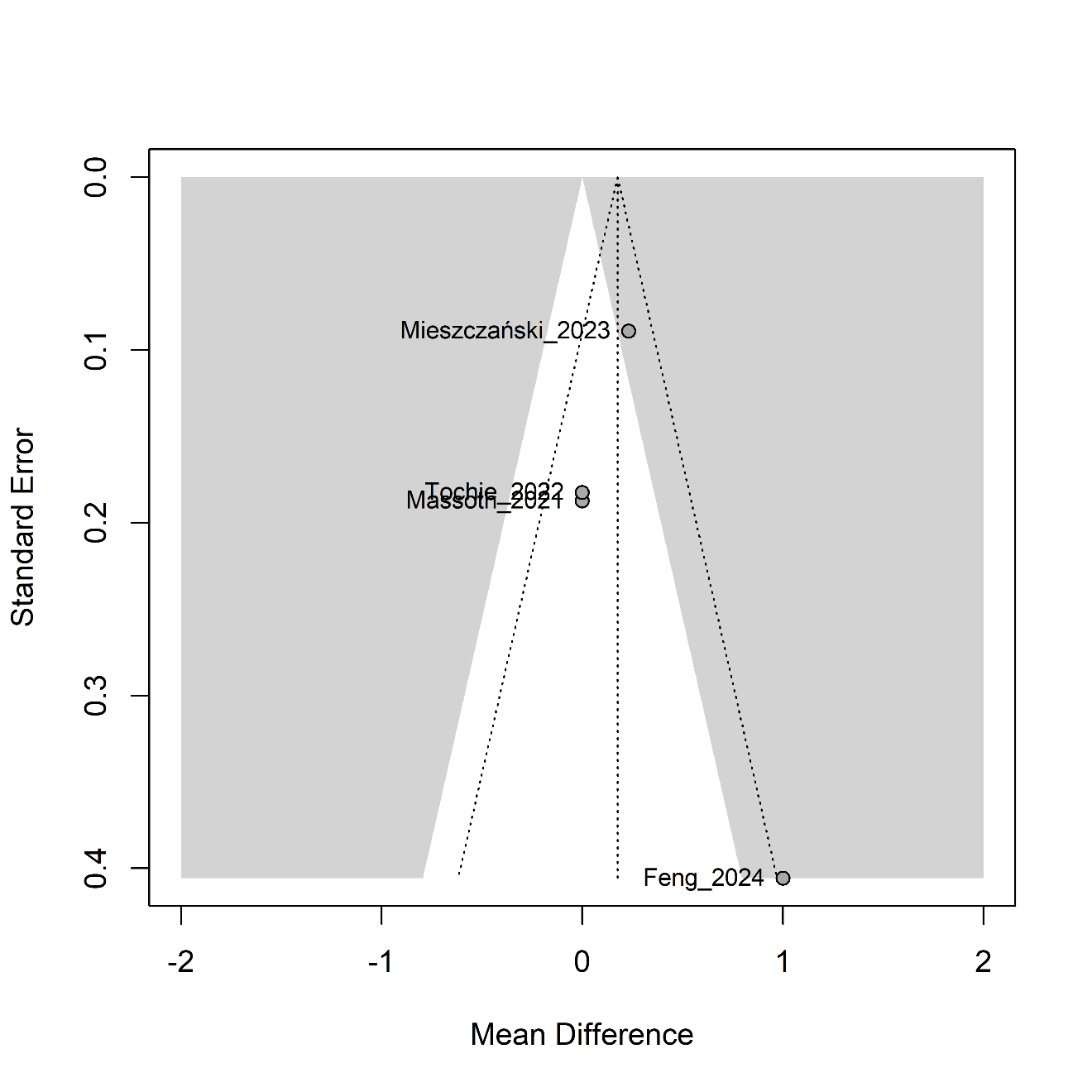


**Adverse events: Supplementary Figures 64-66**

Supplementary Figure 64: Adverse events – Hypotension


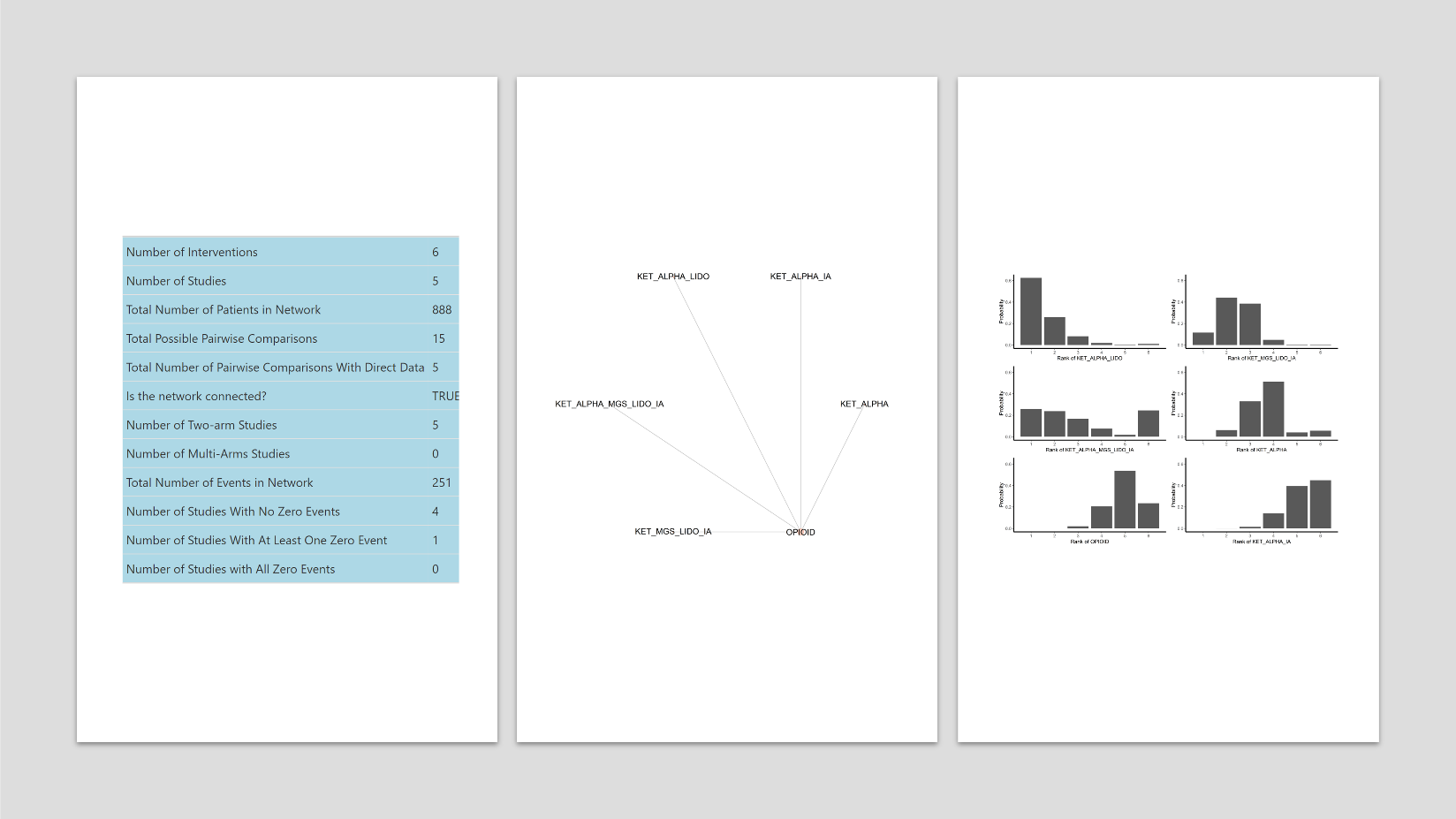


Supplementary Figure 65: Adverse events – Tachycardia


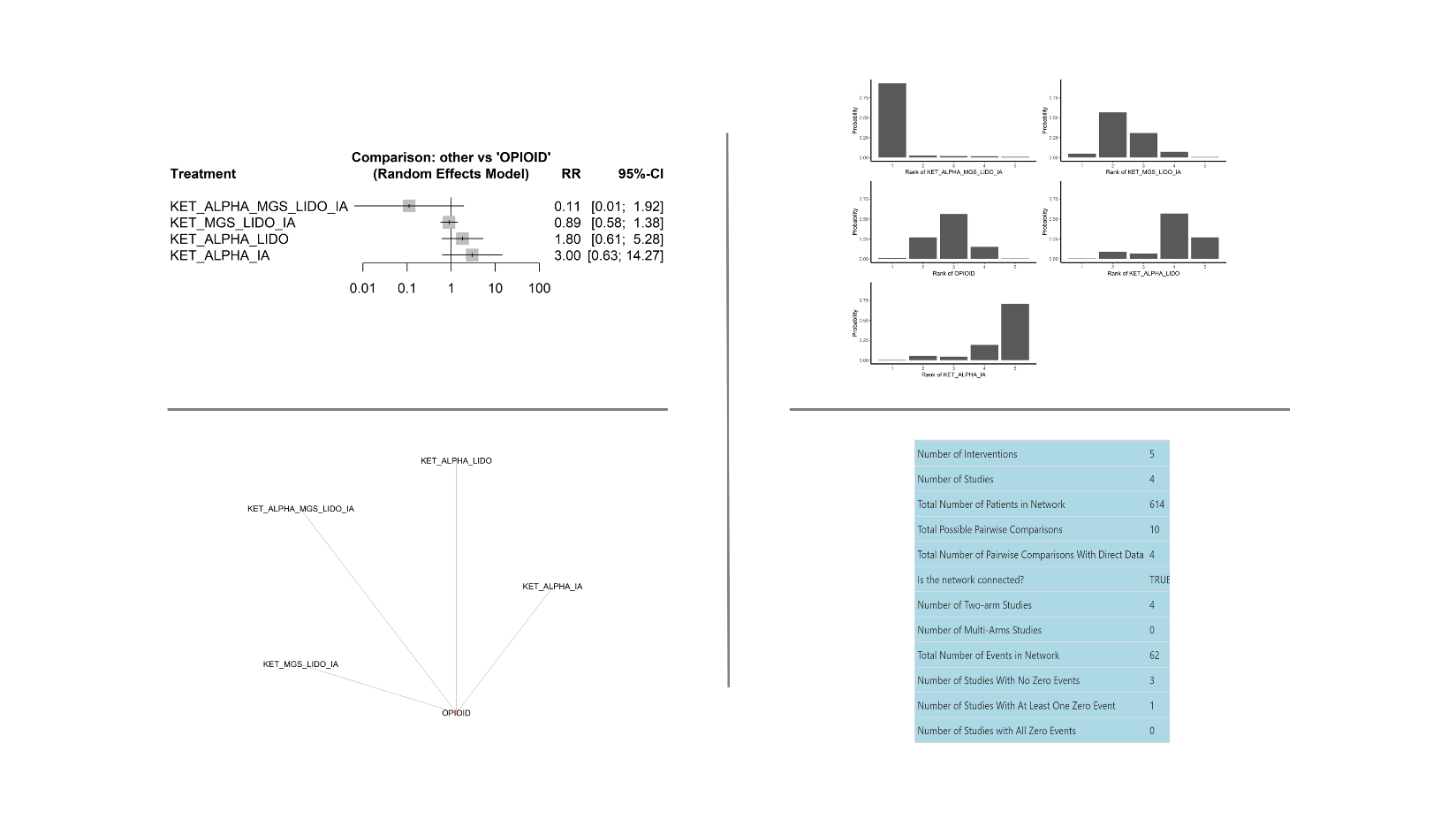


Supplementary Figure 66: Adverse events – Bradycardia


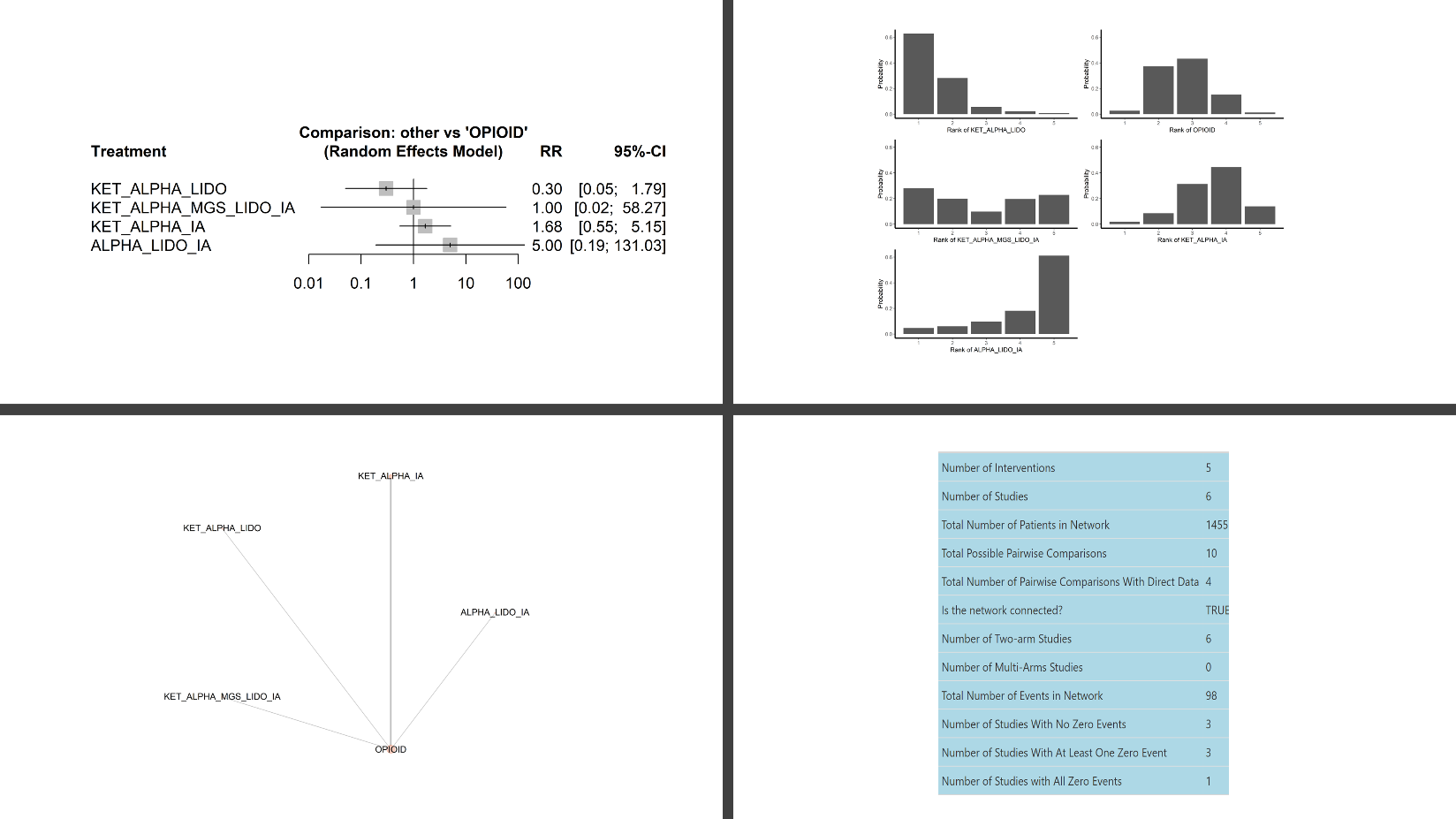


**Search strategy**

## PubMed

((("analgesics opioid"[Pharmacological Action] OR "analgesics, opioid"[MeSH Terms] OR ("analgesics"[All Fields] AND "opioid"[All Fields]) OR "opioid analgesics"[All Fields] OR "opioid"[All Fields] OR "opioids"[All Fields] OR "opioid s"[All Fields]) AND "free"[All Fields] AND ("anaesthesia"[All Fields] OR "anesthesia"[MeSH Terms] OR "anesthesia"[All Fields] OR "anaesthesias"[All Fields] OR "anesthesias"[All Fields])) OR (("analgesics opioid"[Pharmacological Action] OR "analgesics, opioid"[MeSH Terms] OR ("analgesics"[All Fields] AND "opioid"[All Fields]) OR "opioid analgesics"[All Fields] OR "opioid"[All Fields] OR "opioids"[All Fields] OR "opioid s"[All Fields]) AND "free"[All Fields] AND ("anaesthesia"[All Fields] OR "anesthesia"[MeSH Terms] OR "anesthesia"[All Fields] OR "anaesthesias"[All Fields] OR "anesthesias"[All Fields])) OR (("analgesics opioid"[Pharmacological Action] OR "analgesics, opioid"[MeSH Terms] OR ("analgesics"[All Fields] AND "opioid"[All Fields]) OR "opioid analgesics"[All Fields] OR "opioid"[All Fields] OR "opioids"[All Fields] OR "opioid s"[All Fields]) AND ("spare"[All Fields] OR "spared"[All Fields] OR "spares"[All Fields] OR "sparing"[All Fields])) OR (("analgesics opioid"[Pharmacological Action] OR "analgesics, opioid"[MeSH Terms] OR ("analgesics"[All Fields] AND "opioid"[All Fields]) OR "opioid analgesics"[All Fields] OR "opioid"[All Fields] OR "opioids"[All Fields] OR "opioid s"[All Fields]) AND "free"[All Fields])) AND ("clinical trials as topic"[MeSH Terms] OR ("clinical"[All Fields] AND "trials"[All Fields] AND "topic"[All Fields]) OR "clinical trials as topic"[All Fields] OR "trial"[All Fields] OR "trial s"[All Fields] OR "trialed"[All Fields] OR "trialing"[All Fields] OR "trials"[All Fields]) AND ("clinical trials as topic"[MeSH Terms] OR ("clinical"[All Fields] AND "trials"[All Fields] AND "topic"[All Fields]) OR "clinical trials as topic"[All Fields] OR "trial"[All Fields] OR "trial s"[All Fields] OR "trialed"[All Fields] OR "trialing"[All Fields] OR "trials"[All Fields])

## The Cochrane Library for clinical trials in CENTRAL

(opioid):ti,ab,kw AND (free):ti,ab,kw AND (anesthesia):ti,ab,kw (Word variations have been searched) in Trials

## Embase via Elsevier

((("analgesics opioid"[Pharmacological Action] OR "analgesics, opioid"[MeSH Terms] OR ("analgesics"[All Fields] AND "opioid"[All Fields]) OR "opioid analgesics"[All Fields] OR "opioid"[All Fields] OR "opioids"[All Fields] OR "opioid s"[All Fields]) AND "free"[All Fields] AND ("anaesthesia"[All Fields] OR "anesthesia"[MeSH Terms] OR "anesthesia"[All Fields] OR "anaesthesias"[All Fields] OR "anesthesias"[All Fields])) OR (("analgesics opioid"[Pharmacological Action] OR "analgesics, opioid"[MeSH Terms] OR ("analgesics"[All Fields] AND "opioid"[All Fields]) OR "opioid analgesics"[All Fields] OR "opioid"[All Fields] OR "opioids"[All Fields] OR "opioid s"[All Fields]) AND "free"[All Fields] AND ("anaesthesia"[All Fields] OR "anesthesia"[MeSH Terms] OR "anesthesia"[All Fields] OR "anaesthesias"[All Fields] OR "anesthesias"[All Fields])) OR (("analgesics opioid"[Pharmacological Action] OR "analgesics, opioid"[MeSH Terms] OR ("analgesics"[All Fields] AND "opioid"[All Fields]) OR "opioid analgesics"[All Fields] OR "opioid"[All Fields] OR "opioids"[All Fields] OR "opioid s"[All Fields]) AND ("spare"[All Fields] OR "spared"[All Fields] OR "spares"[All Fields] OR "sparing"[All Fields])) OR (("analgesics opioid"[Pharmacological Action] OR "analgesics, opioid"[MeSH Terms] OR ("analgesics"[All Fields] AND "opioid"[All Fields]) OR "opioid analgesics"[All Fields] OR "opioid"[All Fields] OR "opioids"[All Fields] OR "opioid s"[All Fields]) AND "free"[All Fields])) AND ("postoperative period"[MeSH Terms] OR ("postoperative"[All Fields] AND "period"[All Fields]) OR "postoperative period"[All Fields] OR ("post"[All Fields] AND "operative"[All Fields]) OR "post operative"[All Fields] OR ("surgery"[MeSH Subheading] OR "surgery"[All Fields] OR "surgical procedures, operative"[MeSH Terms] OR ("surgical"[All Fields] AND "procedures"[All Fields] AND "operative"[All Fields]) OR "operative surgical procedures"[All Fields] OR "general surgery"[MeSH Terms] OR ("general"[All Fields] AND "surgery"[All Fields]) OR "general surgery"[All Fields] OR "surgery s"[All Fields] OR "surgerys"[All Fields] OR "surgeries"[All Fields])) AND ("pain"[MeSH Terms] OR "pain"[All Fields])

## CINAHL (Cumulative Index to Nursing and Allied Health Literature) via EBSCO

((TI "Opioid-free anesthesia" OR AB "Opioid-free anesthesia") OR (TI "Non-opioid anesthesia" OR AB "Non-opioid anesthesia") OR (TI "Opioid-sparing anesthesia" OR AB "Opioid-sparing anesthesia") OR (TI "Opioid-sparing strategies" OR AB "Opioid-sparing strategies") OR (TI "Total opioid-free general anesthesia" OR AB "Total opioid-free general anesthesia") OR (TI "Opioid-free analgesia" OR AB "Opioid-free analgesia") OR (TI "Opioid eradication" OR AB "Opioid eradication") OR (TI "Opioid-free postoperative analgesia" OR AB "Opioid-free postoperative analgesia") OR (TI "Opioid-free anaesthesia" OR AB "Opioid-free anaesthesia"))

AND ((MH "clinical trials as topic+") OR (clinical AND trials AND topic) OR "clinical trials as topic" OR trial OR "trial s" OR trialed OR trialing OR trials)
